# Spillovers and legacies of land management on temperate woodland biodiversity

**DOI:** 10.1038/s41559-025-02688-6

**Published:** 2025-04-23

**Authors:** Tom Bradfer-Lawrence, Andrew D. M. Dobson, Tom Finch, Elisa Fuentes-Montemayor, Nick Hanley, Jason Matthiopoulos, Mary Nthambi, Katherine Simpson, Kevin Watts, Robin C. Whytock, Kirsty J. Park

**Affiliations:** 1 Biological and Environmental Sciences, University of Stirling, Stirling, UK; 2 Centre for Conservation Science, RSPB, Edinburgh, UK; 3 School of Biodiversity One Health and Veterinary Medicine, University of Glasgow, Glasgow, UK; 4 Forest Research, Alice Holt Lodge, Farnham, UK; 5 Okala Ltd, London, UK

**Keywords:** Community ecology, Conservation biology, Restoration ecology, Biodiversity

## Abstract

Species distributions are a product of both current spatial configuration of habitats and legacies of historical land use. Here we explore current and historical drivers of species distributions, considering combined effects of spatial spillovers and temporal legacies, both within and between habitat types. We fit Bayesian hierarchical occupancy models to data on 373 species from four taxa (ground beetles, birds, vascular plants and small terrestrial mammals) from a chronosequence of 134 woodlands (10 to >250 years old) in temperate agricultural landscapes in the UK. Both spillovers and legacies affect species richness and community composition and, critically, these effects interact. Real-world combinations of spillovers and legacies result in different biodiversity responses compared with the individual factors in isolation. Woodland patches in landscapes with more old woodland and lower amounts of historical woodland loss tend to host more bird and plant but fewer beetle species. Failing to account for these drivers (in particular, legacy effects) gives a distorted view of habitat suitability. In consequence, the same management actions may result in unexpectedly different outcomes depending on the spatial and historical context within the landscape. A better understanding of spillovers and legacy effects on species distributions is required to design biodiversity-friendly, cost-effective land management.

## Abstract (web summary)

Data on beetle, bird, plant and mammal occupancy of woodland sites in UK agricultural landscapes demonstrate how contemporary spatial spillovers and legacies of past land use interact to affect species richness and community composition.

## Main

Species distributions are the product of both spatial spillovers from the surrounding contemporary landscape^1–3^ and temporal legacies from historical land cover and management^4–8^. Thus, a species’ presence could reflect favourable landscape configuration or historical context, rather than contemporary, patch-scale conditions^9–12^. Ignoring temporal influences and their possible interactions with spatial drivers limits our understanding of species distributions and ecosystems^13–17^.

**Fig. 1:**
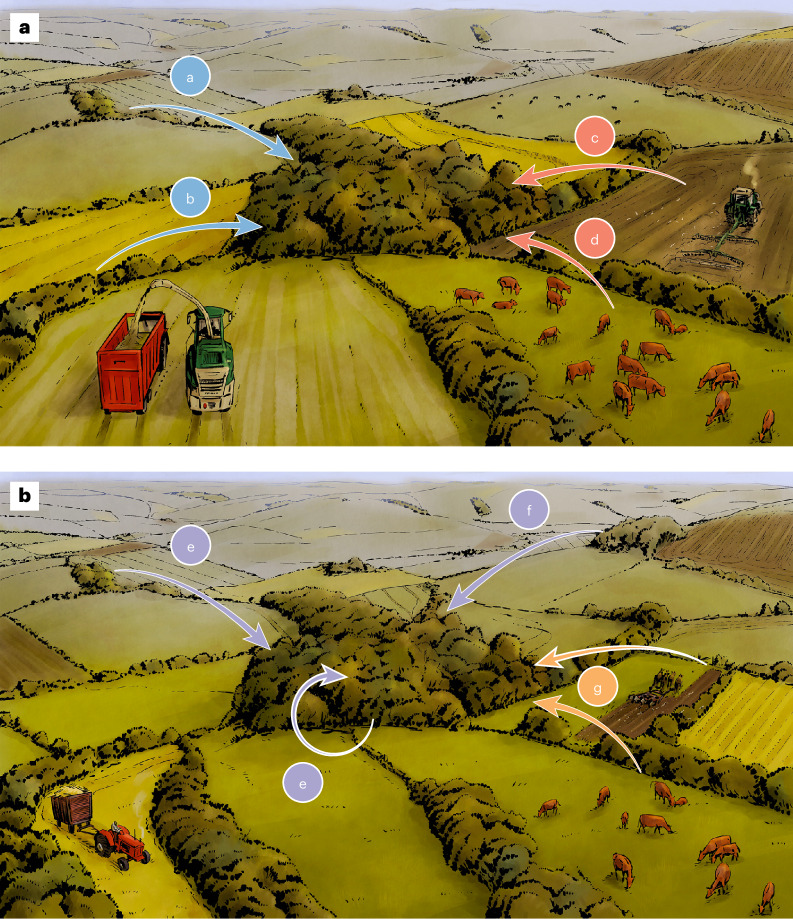
Spatial spillovers and temporal legacies. **a**, A twenty-first century agricultural landscape. Colonization and extinction dynamics of species in the central woodland patch will be influenced by spatial spillovers from the landscape context. These can be within-habitat type (that is, woodland to woodland, in blue), including between patches (a) and via linear features such as hedgerows (b). Between-habitat-type spillovers (in pink) refer to the influence of other habitats, in this case farmland, including measures of intensity such as amount of arable agriculture (c) and livestock density (d). **b**, The same landscape in the mid-twentieth century. Historical land-use patterns that dictated biodiversity of the central woodland patch at the time continue to do so in the present. These temporal legacies can be within-habitat type (in purple), including woodland age (that of both the central patch and surrounding patches) (e) and the loss of neighbouring woodlands (f), or between-habitat type (in orange), such as livestock density and cropping patterns (g). Illustrations by Marco Lawrence.

To gain greater insight into current species’ distributions, here we investigate both spillovers and legacies in combination (Fig. 1). At the landscape scale, spillovers and legacies can occur within a habitat type, that is, in a network of patches of a single habitat type, or between habitat types, that is, from patches of one habitat type to patches of another^18,19^. Legacy effects vary over time in a single patch (for example, as vegetation structure develops) or occur among multiple patches (for example, by providing source populations for colonization) (Fig. 1).

Unrecognized or unquantified drivers of species’ distributions could have major implications, as the same management actions might have radically dissimilar (even opposing) effects in different locations^20^. Interventions targeting spatial spillovers such as increasing connectivity among patches of the same habitat type^21^ may be appropriate for short-ranging taxa that need contiguous habitat to disperse^17,22^, while wide-ranging species may prefer lower-intensity management over large areas. However, intervention efficacy will be mediated by temporal legacies. For example, if historical land clearance has diminished the seed bank, dispersal-limited plants could be delayed or prevented from colonizing newly created habitat patches^23^, even if these are well connected^4^. Critically, the relative importance of spillovers and legacies will probably vary among taxa with different ecological traits^24^.

Although individual spillover and legacy effects are well recognized, effective synthesis is often prohibited by a lack of multitaxa occurrence data from a common set of sites with known landscape history^5,25^. Here, we used data from a chronosequence of woodlands in temperate, lowland agricultural landscapes in the UK. Planted woodlands were 10–160 years old^26^, with additional ‘ancient’ woodlands over 250 years old^27^. We examined the effects of spillovers, both within and between habitats, and temporal legacies on the occupancy of four contrasting taxa (beetles, birds, plants and mammals; Supplementary Table 1). The planted woodlands were created on former agricultural land, and hence, their biodiversity dynamics may principally reflect colonization from the surrounding landscapes rather than extinction debt processes typically associated with the fragmentation of larger blocks of habitat^28^. We sought to assess overall effects of woodland creation on biodiversity, and study sites often lacked high numbers of specialist species owing to limited colonization of planted woodlands. We therefore included all species irrespective of habitat affinity, rather than limiting our analyses to only woodland specialists. We considered predictors individually and in combination, to explore species-level responses and the consequences for community-level richness and composition in woodland patches.

## Results

We used Bayesian joint species occupancy models^29,30^ to explore spatial spillovers and temporal legacies, using a common model structure across the four taxonomic groups. These models incorporated an extensive suite of site- and landscape-scale predictors, covering both contemporary spatial and historical legacy patterns. Using posterior estimates from the models we simulated assemblages of each taxonomic group in response to different landscape configurations. These used real-world values drawn from the ranges of the original predictor variables, thus propagating full uncertainty from the posterior. We converted these probabilities to presence–absence using Bernoulli trials and examined both species richness and assemblage composition responses.

### Within-habitat-type spillovers

We expected larger woodland patches to support more species^1^ but anticipated that this would be mediated by habitat composition and configuration in the broader landscape. Greater amounts of woodland, and hedgerows and other trees outside woodlands, are likely to increase matrix permeability^31,32^, leading to greater dispersal, colonization and, thus, species richness.

**Fig. 2:**
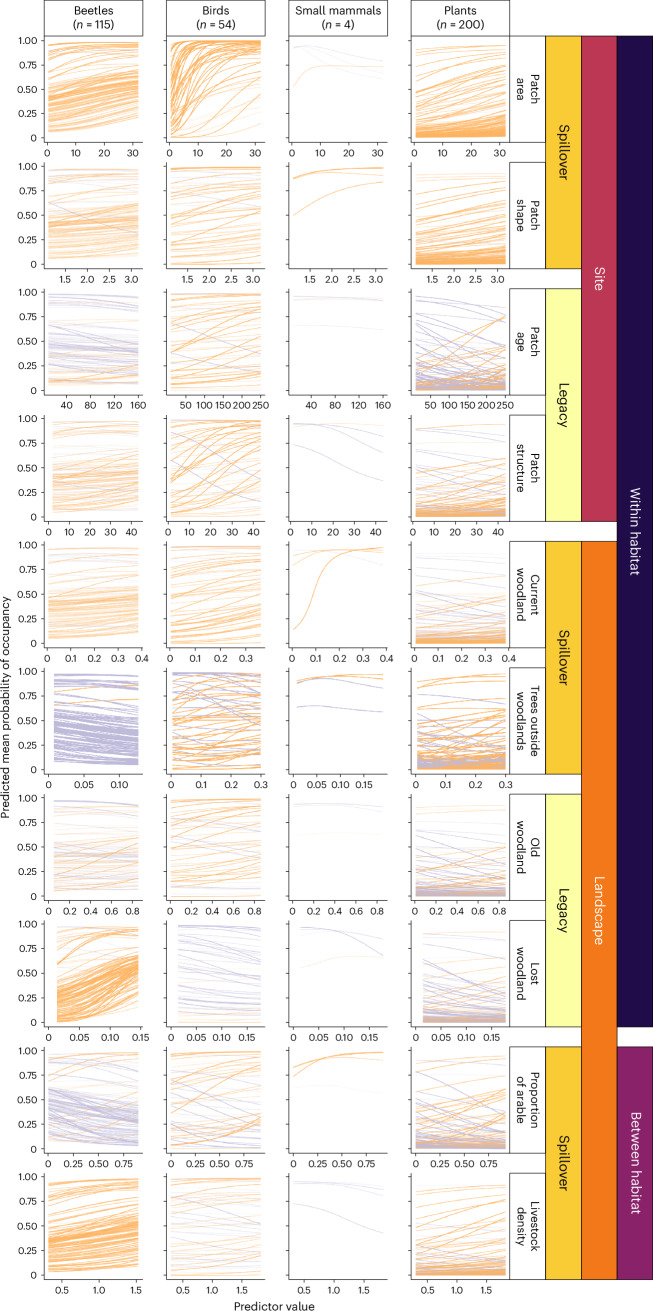
Predicted changes in individual species’ probability of occupancy in response to spatial spillovers and temporal legacies. Marginal response curves were generated using the posterior means for each species’ intercept and the relevant slope term, with all other predictors held at their mean. The *x* axes cover only the range of values in the original datasets and, hence, vary among taxa. The orange lines show positive responses, purple lines show negative responses, and transparency reflects the probability of direction^88^. The darker the lines, the greater the proportion of the posterior that is positive (or negative) and, hence, the greater the support. The darkest lines show species with a probability of direction of 100%, and the palest 50%. Source data

**Extended Data Fig. 1:**
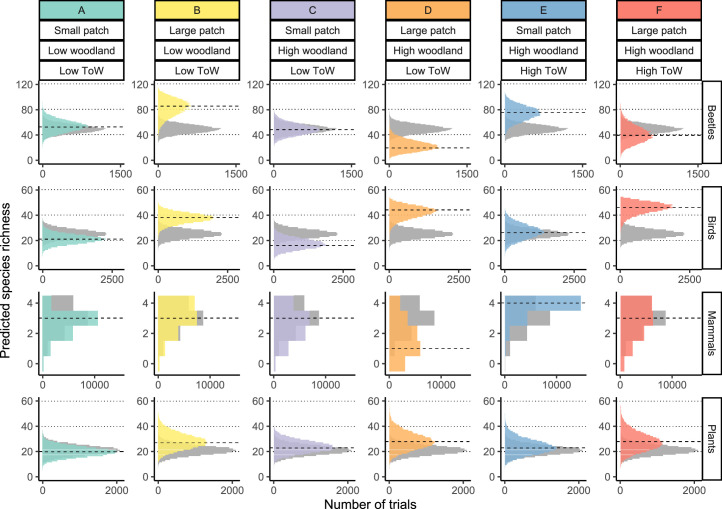
Assemblage species richness in response to within-habitat-type spillovers. Combinations of three variables: woodland site area (0.5 ha vs 15 ha); proportion of woodland cover in the landscape (0.01 vs 0.3); proportion of trees outside woodlands (ToW) in the landscape (0.01 vs 0.15). Dashed lines show modal values, grey histograms are assemblages from ‘background’ landscapes. Source data

As expected, the probability of occupancy increased for most species with greater woodland patch area (Fig. 2). This was particularly marked for birds, which had much higher species richness in larger sites irrespective of landscape context (Extended Data Fig. 1). Conversely, three of the four mammal species showed a slight negative response to increasing patch area. There was also a positive effect of less compact woodlands on occupancy probability for all taxonomic groups (Fig. 2). Beyond the scale of the focal patch, higher levels of current woodland in the landscape and trees outside woodlands led to increased probability of occupancy of a woodland patch for most species (Fig. 2). However, this translated into only slightly greater species richness in simulated assemblages (Extended Data Fig. 1). Beetles showed contrasting responses to landscape-scale tree cover, with a moderate positive effect of current woodland in the landscape but negative responses to trees outside woodlands when examined individually (Fig. 2). Simulated beetle assemblages in large sites were more species rich in landscapes with low levels of woodland but species poor in landscapes with high levels of woodland (Extended Data Fig. 1). Plant assemblages had fractionally greater species richness in larger sites and in landscapes with higher levels of current woodland (Extended Data Fig. 1).

**Extended Data Fig. 2:**
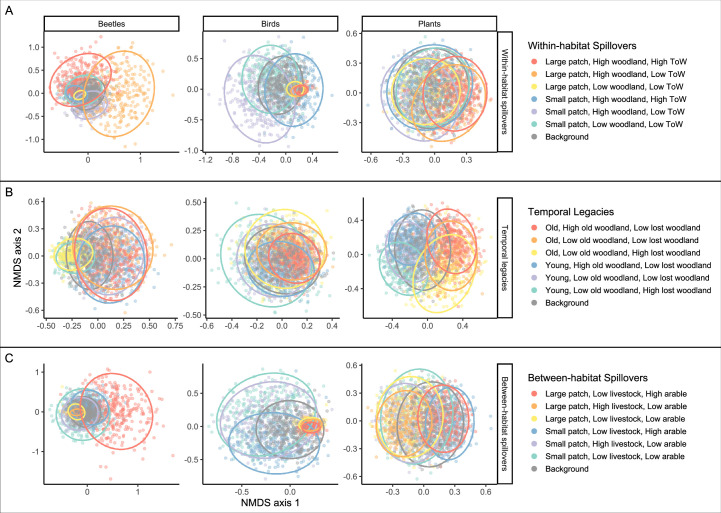
Assemblage compositional responses to spatial spillovers and temporal legacies. Ordination plots using Non-metric Multidimensional Scaling (NMDS) for **A**: Within-habitat spillovers, **B**: Temporal legacies, and **C**: Between-habitat spillovers. Each dot represents a simulated assemblage. Each panel shows a representative, random 10% of 20,000 assemblages generated from model posterior samples, with 89% ellipses. Slope parameters were fixed at one of two levels as per Extended Data Figs. 1, 3 and 4. ‘ToW’ stands for ‘Trees outside Woodlands’. Source data

Species assemblages simulated from model posterior estimates showed clear compositional patterns for birds; all large sites were tightly clustered in ordination space regardless of landscape context because most species occupied all sites (Extended Data Fig. 2a). Overlapping ellipses show that smaller sites hosted subsets of the entire assemblage irrespective of landscape context. The trend in beetle assemblages was less consistent. In landscapes with low levels of current woodland and trees outside woodland, assemblages at larger sites were more tightly clustered, suggesting little turnover (Extended Data Fig. 2a). Conversely, in landscapes with higher levels of woodland, simulated assemblages for larger sites were species poor and so were more dispersed than those for smaller sites (Extended Data Fig. 2a). Plant assemblages were impervious to patch area, but higher values on the first ordination axis were associated with more woodland and trees outside woodland in the landscape (Extended Data Fig. 2a).

### Legacies

Woodland patch age and woodland dynamics in the surrounding landscape (the amount of woodland that has persisted or been lost over the past century) reflect ecological continuity^17,33^. These potentially slow down extinction debts or provide source populations for colonization credits once a new habitat patch is sufficiently developed^13,24^. In consequence, we expected that older woodland patches in more stable landscapes (that is, those with higher amounts of old woodland and lower levels of woodland loss) should have greater species richness and a different community composition compared with younger woodland patches^34^.

**Extended Data Fig. 3:**
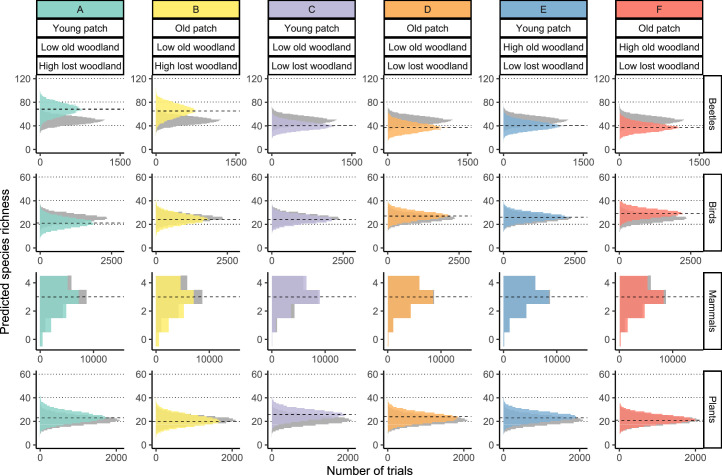
Assemblage species richness in response to within-habitat-type temporal legacies. Combinations of three variables: woodland site age (10 vs 250 years); proportion of old woodland in the landscape (0.01 vs 0.85); proportion of woodland lost from the landscape (0.01 vs 0.15). Dashed lines show modal values, grey histograms are assemblages from ‘background’ landscapes. Source data

There was substantial within-taxa variability in probability of occupancy with increasing site age (Fig. 2). The effect of age per se on the species richness of simulated assemblages was therefore weak in comparison with the effects of within-habitat-type spillovers (Extended Data Fig. 3). However, effects of woodland age are mediated by vegetation structure, and older sites typically contain greater structural heterogeneity^27,35^. More heterogeneous woodland structure (that is, greater variation in tree diameter at breast height (DBH) was associated with an increased probability of occupancy for most species except mammals (Fig. 2). Landscape-scale legacy effects were smaller than site-scale effects. Only birds showed clear taxon-level increases in occupancy probability with higher levels of old woodland and lower amounts of historical woodland loss (Fig. 2). Beetles had a consistent positive response to historical woodland loss, and this was reflected in simulated species richness (Extended Data Fig. 3).

Legacies had some effect on simulated beetle assemblage composition, with tighter clustering of sites in landscapes with high levels of woodland loss, irrespective of site age (Extended Data Fig. 2b). Although site age did not strongly affect simulated bird and plant species richness, there were clear patterns in both bird and plant assemblage composition. Old sites were associated with greater values on the second ordination axis for birds and the first ordination axis for plants (Extended Data Fig. 2b). There was also a moderate effect of higher levels of old woodland and lower levels of lost woodland on axis 1 for birds and axis 2 for plants (Extended Data Fig. 2b).

### Between-habitat-type spillovers

Agricultural intensity in the surrounding landscape can have strong effects on species distributions^36–39^. We anticipated that woodland patches in more intensively farmed landscapes (that is, higher density of grazing livestock or higher amounts of arable agriculture) would host fewer species and have a different community composition compared with woodland patches in less-intensively farmed landscapes. However, larger woodland patches should buffer external influences, reducing these spillover effects.

**Extended Data Fig. 4:**
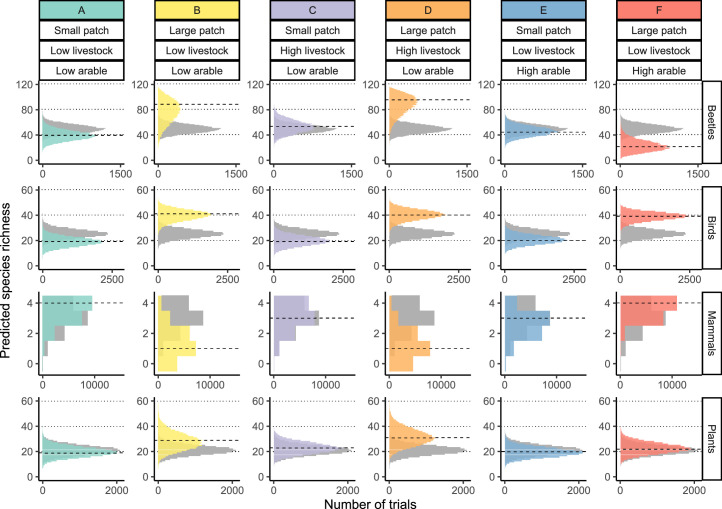
Assemblage species richness in response to between-habitat-type spillovers. Combinations of two variables: livestock density (0.3 vs 1.5 LSU/ha) and proportion of farmland under arable crops (0.01 vs 0.9), both mediated by woodland site area (0.5 ha vs 15 ha). Dashed lines show modal values, grey histograms are assemblages from ‘background’ landscapes. Source data

There was considerable intrataxon variability in the response of occupancy probability to agricultural intensity (Fig. 2). These between-habitat-type spillovers were mediated by woodland site area, with larger sites buffering the effects of intensive agriculture on species richness in most cases (Extended Data Fig. 4). Beetle occupancy probability was the exception, with the negative effect of greater arable intensity sufficient to counter the positive effect of site area (Fig. 2 and Extended Data Fig. 4), and this was associated with greater variability in assemblage composition (Extended Data Fig. 2c). Bird species richness was largely unaffected by either arable agriculture or livestock density because of the strong effect of woodland site area (Extended Data Fig. 4). The first ordination axis for birds was driven by woodland site area, and livestock density did not influence composition; however, increased arable agriculture created a distinct assemblage (Extended Data Fig. 2c). There were fewer mammal species in large woodlands, except in landscapes with a high proportion of arable agriculture (Extended Data Fig. 4). Plant species richness was largely unaffected by livestock density, but a high proportion of arable agriculture suppressed the positive effect of large woodland sites (Extended Data Fig. 4). This was reflected in higher values on the first ordination axis, suggesting that plant assemblages in arable landscapes are distinct from others and less influenced by woodland site area in this context (Extended Data Fig. 2c).

### Combined spillovers and legacies

We examined the combined effects of current landscape (amount of woodland and trees outside woodlands), historical stability (proportions of old woodland and lost woodland) and agricultural intensity (livestock density and arable agriculture) on biodiversity in a hypothetical woodland patch of mean size (3.5 ha). We predicted that the three types of drivers would interact, so that the specific spatiotemporal context would mediate the biodiversity responses in individual woodland patches, although we expected the precise effects to vary among taxa.

**Fig. 3:**
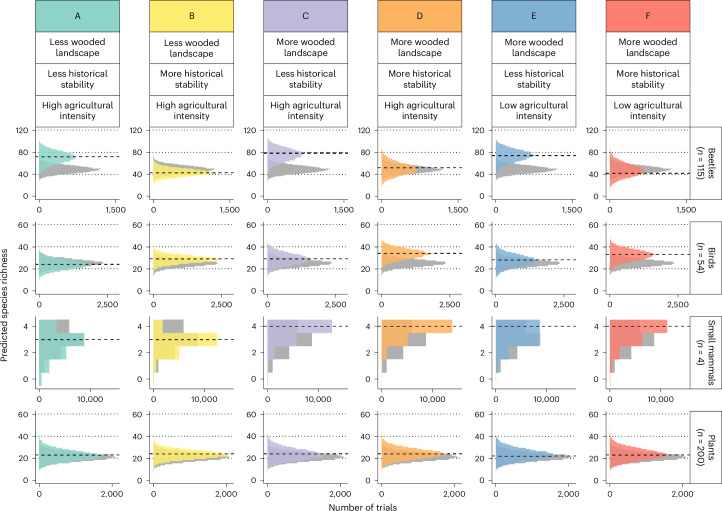
Effects of spillovers and legacies on species richness. Assemblage species richness in a 3.5 ha woodland in response to combinations of within-habitat-type spillovers (proportion of woodland cover in the landscape, 0.01 versus 0.3); temporal legacies (proportion of old woodland in the landscape, 0.01 versus 0.85, and proportion of woodland lost from the landscape, 0.01 versus 0.15); and between-habitat-type spillovers (proportion of farmland under arable crops, 0.01 versus 0.9). The dashed lines show modal values, grey histograms are assemblages from ‘background’ landscapes, and dotted lines are graphical only to aid comparison among panels. Source data

The combined spatiotemporal context influenced species richness and community composition, with some drivers countering the effect of others. Beetle assemblages were more species rich in landscapes with less historical stability (Fig. 3). Birds had higher species richness in more wooded landscapes with greater historical stability, but the effect of agricultural intensity was minimal. There were more small mammal species in more wooded landscapes. Plant species richness remained similar across spatiotemporal contexts.

**Fig. 4:**
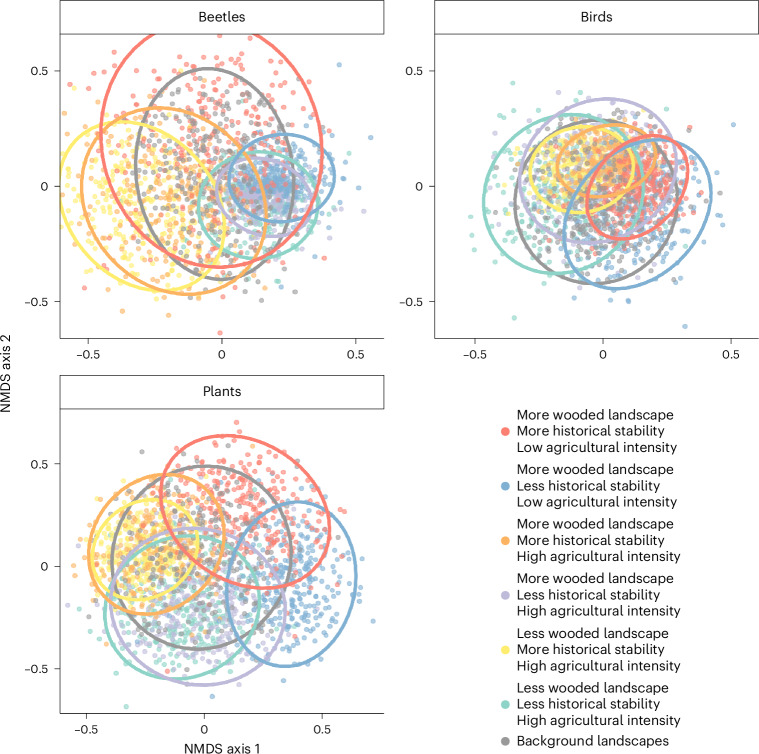
Effects of spillovers and legacies on assemblage composition. Ordination plots using NMDS, with each dot representing a simulated assemblage. Each panel shows a representative, random 10% of 20,000 assemblages generated from model posterior samples, with 89% ellipses. Slope parameters were fixed at one of two levels as per Fig. 3; site area was fixed at the mean value of 3.5 ha; all other linear predictors were included with a random value drawn from the marginal distribution of the original data. ‘Background’ landscape assemblages generated using random values for all predictors except site area. NMDS stress values range from 0.15 to 0.3. Source data

The composition of simulated beetle assemblages was particularly variable in sites with greater historical stability (Fig. 4) with a clear but smaller effect of levels of woodland in the landscape and agricultural intensity. Bird assemblage composition was driven by spillovers and legacies; higher values on ordination axis 1 were associated with more benign spatiotemporal context, that is, more wooded landscapes and lower agricultural intensity. Given that agricultural intensity did not affect avian species richness, this implies turnover in the assemblages. Compositional patterns in plant assemblages were similar to those of birds; higher values on ordination axis 1 were associated with more wooded landscapes and less intensive agriculture, and higher values on axis 2 with more historical stability.

## Discussion

We investigated the influence of spatial spillovers and temporal legacies on woodland biodiversity patterns in two temperate, lowland, agricultural landscapes. We explored species-specific responses to site and landscape variables, and the consequences for species richness and assemblage composition in woodland patches. Many spillovers and legacies had relatively weak effects when considered individually. Yet, critically, we demonstrate that the collective spatiotemporal spillovers (reflecting real-world combinations of these drivers) resulted in stronger effects, and often with different patterns compared with the individual spillovers in isolation.

In general, larger woodlands in landscapes with greater levels of woodland cover and trees outside woodlands will support more species, particularly in areas with less intensive arable agriculture. However, the legacies of historical land-use patterns can mediate these beneficial effects. A woodland patch in a landscape with greater historical stability (that is, fewer changes in landscape-scale woodland cover) will host more bird and plant species, and a distinct subset of bird, plant and beetle species typically not found in landscapes with a less stable land-use history. A deeper understanding of the relative importance of—and interactions between— current and historical drivers of species distributions can help inform the design and delivery of future land management actions.

Beetle species richness was higher in large woodlands than small woodlands in landscapes with low levels of woodland cover, implying that these woodlands may act as refugia in intensively farmed landscapes. However, species richness in large woodlands was lower in landscapes with high levels of woodland cover and high historical stability. This may be because species in our woodland sites were largely woodland generalists resilient to landscape-level disturbance^40^. Such generalists may be less prevalent in stable landscapes, but the specialists that are present are unlikely to colonize newer woodlands, hence leading to a reduction in species richness rather than turnover. Species richness was also slightly higher in younger than in older sites. This mirrors research from UK conifer plantations showing declines in carabid species richness and functional diversity as trees mature and canopy cover increases^41,42^. Conversely, lower tree density and maintenance of open rides appears to support greater species richness in woodlands^43^. Effects of woodland patch area varied depending on agricultural context, with higher species richness in large woodlands in landscapes with high livestock density and in smaller woodlands in landscapes with high levels of arable agriculture. In contrast to many other invertebrate groups in the UK, carabids have higher occupancy in agricultural areas dominated by arable cropland^38^.

Birds were more sensitive to spillovers than legacies when these effects were examined individually^14^. Bird assemblages had higher species richness in larger woodland patches, especially in landscapes with more current woodland. Larger woodlands often host more woodland-affiliated species^44,45^, and site factors can outweigh landscape-scale effects^46^. When other drivers were held constant, the legacy effects on simulated species richness were relatively small, suggesting that birds will respond rapidly to changes in landscape conditions because of their relatively high dispersal ability. Patch age, however, did influence assemblage composition, indicating the existence of a subset of species affiliated with older woodlands. Moreover, in the combined analysis, there were distinct increases in species richness and changes in assemblage composition associated with greater historical stability. While agricultural intensity has previously been shown to have marked impacts on farmland-affiliated bird populations^39,47,48^, in our study the size of the focal woodland strongly mediated any other within- or between-habitat-type spillover. We posit two potential, non-mutually exclusive mechanisms: first, larger sites mitigate any negative spillovers such as agrochemical drift from neighbouring land uses^34,49^ and, second, agricultural intensification operates over much larger scales so that consequences are seen at the regional level rather than in individual sites^15,39^.

Small terrestrial mammals are sensitive to within-habitat-type spatial spillovers, being more likely to occupy small, compact woodlands. Between-habitat-type spillovers were also important, with higher occupancy of woodland patches in landscapes with a higher proportion of arable agriculture. This mirrors existing studies that reported that greater landscape-scale agricultural intensity was associated with greater small mammal species richness^20^ and abundance^50^. However, these species still require some less-intensively managed habitats, and creating uncropped field margins and greater landscape-scale habitat diversity have both been associated with increased small mammal occupancy and abundance^51^. Thus, even small woodlands and hedgerows can be important in intensively managed landscapes, ensuring that generalist species can persist^20^.

Plants were most strongly influenced by woodland patch area and proportion of arable agriculture and to a lesser extent by the current woodland in the landscape. The lack of clear species richness patterns in the combined spatiotemporal analysis is probably due to our inclusion of all vascular plant species in our analysis. Both historical stability and agricultural intensity drove turnover in the plant assemblages, probably reflecting replacement of early-successional species with more specialist woodland species. The latter species are often dispersal-limited so that their presence is determined by site area and surrounding woodland cover^17,52^, as well as localized structural and edaphic qualities^35,53,54^.

There was notable inter- and intrataxon variation in responses in our study. These differences are probably determined by ecological traits, including demographic patterns and dispersal abilities^12,55,56^, although we did not test for these here. Some species appeared impervious to changes in the predictors, or even exhibited contrasting responses to related taxa. This may be due to specialist requirements, such that the broad trends discussed here may obscure important patterns in species of conservation concern^15^. We did not examine potentially contrasting responses of generalists and specialists here, but there are many studies that have done so^23,35,40,54^. While generalists’ occupancy may only depend on the presence of a wider habitat network to facilitate dispersal^44^, specialists require the presence of particular features to persist. For instance, the greater spotted woodpecker *Dendrocopos major* relies on deadwood^57^. Colonization and occupancy are therefore dependent on habitat suitability as well as availability^9^, and additional management intervention is often required to encourage particular habitat features needed by specialist species^58^.

Inevitably, there will be winners and losers in response to habitat change. We are unable to assess the net effects of woodland creation on biodiversity in the wider landscape, as our sampling was restricted to the woodland sites themselves, and not the habitats that they might replace. However, woodland creation is often associated with trade-offs^59–61^, and there are legitimate concerns around the impacts that this can have on agriculture-affiliated and open-habitat species. For example, afforestation can severely impact wading birds, through both direct loss of habitat and indirect predation pressures^62^. Minimizing potential trade-offs requires careful spatial planning and in some cases can be mitigated with additional management interventions. For example, declines in agriculture-affiliated granivorous birds have largely been driven by changes in farming practices rather than afforestation, and negative population trends can be countered with targeted conservation actions^63,64^.

Limitations in our results largely arise from data availability. For example, various measures of agricultural intensification—in particular, the use of pesticides and fertilizers—are known to impact wildlife^38,39,65,66^. However, there are no historical records of these inputs at a sufficiently fine spatial resolution that would permit an analysis of between-habitat-type temporal spillovers (Supplementary Information section 3c). Furthermore, we opted for a uniform approach to facilitate cross-taxa comparisons, so inevitably we excluded predictors that could be very important for certain individual taxonomic groups. For instance, the minimal effects of within-habitat-type spillovers on plant assemblages may well stem from our exclusion of relevant factors such as edaphic qualities that drive assemblage composition^53^. We also sought to focus on predictors that were amenable to manipulation by changes to land management, because we wished to relate our findings to the design of agri-environment schemes. We focused on woodland as a key semi-natural habitat that is often the target of conservation and restoration efforts, and one that can be created with relative ease, even if it takes time to mature. Finally, we assessed biodiversity at only a single point in time, yet the landscapes and drivers remain dynamic^8,67^. Shifting assemblages in the wider landscape will influence the species pool and colonization potential^17,24,68^.

Our study areas are dominated (>70%) by agricultural land, typical of lowland landscapes in the UK, and are likely to reflect other temperate regions that have undergone similar historical large-scale forest loss. Given that agriculture is the dominant land use in many temperate regions of the world, land managers must address the current biodiversity crisis by working within agricultural landscapes, balancing interventions against food production and other land-use demands^61,69^. National and international policy commitments relating to biodiversity conservation and climate change mitigation are expected to drive large-scale land-use change in coming decades^70^. These will intersect with rising demands for increased domestic food security, changes in food demand and the trend towards consolidation of individual farms, as well as specific national contexts, such as changing agricultural policy following the UK’s exit from the European Union. Navigating these changes will require flexible strategies and cost-effective interventions, which, in turn, rely on a thorough understanding of the drivers of wildlife distributions. This understanding must encompass both spatial spillovers, such as the impacts of agricultural land management on adjacent habitat, and temporal spillovers, such as the amount of semi-natural habitat lost to agricultural expansion in the last century. As we show here, ignoring how these spillovers interact can lead to erroneous conclusions being drawn on the effects of land-use change on species distributions.

## Methods

### Study sites

Biodiversity data came from 134 woodland sites in the Woodland Creation & Ecological Networks (WrEN) project^26,27^. These sites are situated in lowland agricultural landscapes in two countries of the UK, comprising woodlands planted between 10 and 160 years ago (at the time of surveys), plus ‘ancient’ woodlands at least 250 years old. All planted sites were under agricultural land use for an extensive period before woodland creation. The two study regions cover 7,355 km^2^ (Scotland) and 8,570 km^2^ (England). Individual sites range in size from 0.5 to 31.9 ha. Most sites are at least 3 km (a minimum of 1 km) from each other. Rather than following the development of individual sites, the WrEN project is a natural experiment that uses a space-for-time design to explore the effects of woodland creation. While regional-scale (that is, hundreds to thousands of squared kilometres) land-use patterns are largely fixed, our study areas show high levels of local spatial and temporal variability (for example, in the amount and spatial configuration of woodland, and agricultural intensity of surrounding land, both now and in the past). This allows us to separate the effects of landscape composition (for example, habitat change) from configuration (for example, habitat fragmentation^71,72^) and disentangle spillovers and legacies. Such woodlands make an ideal case study as they are discrete patches surrounded by non-woodland habitats but connected to a wider woodland network in the landscape via features such as hedgerows^73^. Full details of site selection are in refs. ^27,26^.

### Biodiversity data

We collected occupancy data for four taxa covering 373 species, sampling only inside the woodland sites, not the surrounding landscapes. Ground beetles (170 species) were sampled using a network of pitfall traps at 60 sites in 2013 and 2014. Birds (54 species) were surveyed using Common Bird Census methodology^74^ at 125 sites in 2015 and 2017. Small terrestrial mammals (4 species) were live-trapped at 100 sites in 2013 and 2014. Vascular plant assemblages (200 species) were assessed using a comprehensive walkover of 132 sites in 2016. Where data collection covered 2 years, individual sites were surveyed during only 1 year. Full data collection details are presented in Supplementary Information section 1.

### Predictor variables

Full details of predictor variable preparation are in Supplementary Information section 2. Predictors were selected on the basis of those used in previous studies at the same sites (for example, refs. ^24,27,75^) and the wider literature. We examined four site-scale variables. Two spillovers were woodland site area (ha) and shape, both derived from National Forest Inventory mapping^76^ using geographic information systems (GIS). Shape was the woodland patch perimeter divided by the perimeter of a circle with the same area; larger values indicate less-compact sites. Two site-scale temporal legacies were site age and vegetation structure heterogeneity. We used the standard deviation of tree DBH in centimetres as a proxy for structural heterogeneity of vegetation^27^. Structural heterogeneity is initially low in young woodlands and develops over time, but this process results from a combination of site development and management practices rather than chronological age per se. We determined woodland age in years using the Ancient Woodland Inventories^77,78^ or historical Ordnance Survey mapping for planted sites (acknowledging that there may have been a delay between planting and appearance on the map^26^).

We calculated six landscape-scale predictors, defining the landscape as the 3-km radius around each woodland site^75^. Although the specific radius might influence results^79^, in our study regions the landscape variables are strongly correlated between 1 km and 3 km (Supplementary Information section 3). Using a range of historical and contemporary mapping sources, we identified all woodlands greater than 0.5 ha in size at four points in time (1920s, 1950s, 1990s and 2015; Supplementary Information section 2b). From these layers, we calculated three measures of woodland cover: (1) current woodland was the proportion of the surrounding 3-km landscape with woodland in 2015; (2) old woodland was the proportion of the current woodland that had been present at all four points in time and was therefore at least 100 years old; (3) lost woodland was the proportion of the 3-km landscape that had been wooded during at least one of the historical timepoints but was not woodland in 2015. We supplemented the woodland cover predictors with a ‘trees outside woodlands’ dataset for 2015 (ref. ^80^). This provided the proportion of the landscape covered by isolated trees, linear hedgerows and woodlands smaller than 0.5 ha. Current woodland and trees outside woodland were expected to drive within-habitat-type spillovers, and old and lost woodland was expected to influence temporal legacies.

To assess between-habitat-type spillovers, we generated two measures of agricultural intensity for the same 3-km-radius landscapes. Using LCM2015 data^81^, we calculated the proportion of agricultural land that was used for arable farming. We used AgCensus data^82^ derived from the UK’s annual June Agricultural Census to calculate the density of grazing livestock (that is, cattle and sheep), expressed as mean livestock units per hectare of grassland. We considered pesticide usage as an additional measure of agricultural intensity (influencing between-habitat-type spillovers) but excluded it from further analyses as it was very highly correlated with proportion of arable land (Supplementary Information section 4).

We explored potential multicollinearity among predictors using variance inflation factors with the ‘usdm’ R package (v2.1.7)^83^. All variance inflation factors were <2.5, suggesting that collinearity was not liable to cause unstable and uncertain parameter estimates. All predictor variables were centred and scaled before analyses.

### Modelling

To estimate the response of species occupancy to spatial spillovers and temporal legacies, we used Bayesian spatial factor joint-species models using a common structure across the four taxonomic groups^29,30^. Survey design for the three animal taxa included repeated visits, allowing us to implement a hierarchical element to estimate detection probability. Detection (1) or non-detection (0) of the *i*th species at the *j*th site on the *k*th visit is an output of combined detection and occupancy processes, so that

$${Y}_{i,\,j,k} \sim {{\mathrm{Binomial}}}({P}_{i,\,j,k}{,\varPsi }_{i,\,j}),$$

where *Y* is distributed (~) according to *P*_*i,j,k*_, the probability that the species will be detected, and *Ψ*_*i,j*_, the true occupancy status (that is, 0 or 1, assumed to remain constant across all visits). For the animal taxa, the detection process was modelled as

$$\begin{array}{rcl}{{\mathrm{logit}}}({P}_{i,\,j,k}) & = & a{0}_{i}+a{1}_{i}\times {\mathrm{ordinal}}\,{\mathrm{day}}_{j,k}+a{2}_{i}\times {\mathrm{ordinal}}\,{\mathrm{day}}_{j,k}^{2}\\ & & +a{3}_{i}\times {\mathrm{year}}_{j,k}+a{4}_{i}\times {{\mathrm{variable}}}{4}_{j,k}\end{array},$$

where ‘ordinal day’ indicates the day of the year, included as both linear and quadratic terms to account for potential temporal effects on detectability over the course of the surveys, and ‘year’ is the year of the survey. The fourth, taxon-specific variable was included only in the beetle and bird models: for beetles, this was the number of pitfall traps per site; for birds, it was surveyor identity (Supplementary Information section 1). The plant data came from a single exhaustive survey, so we assumed perfect detection and modelled occupancy as

$${Y}_{i,\,j} \sim {{\mathrm{Binomial}}}({\varPsi }_{i,\,j}).$$

True occupancy for all four taxa was modelled as

$$\begin{array}{l}{{\mathrm{logit}}}({{\varPsi }}_{i}({s}_{j}))=b{0}_{i}+b{1}_{i}\times {\mathrm{age}}_{j}+b{2}_{i}\times {\mathrm{area}}_{j}+b{3}_{i}\times {\mathrm{shape}}_{j}+b{4}_{i}\\\qquad\qquad\qquad\times\,{\mathrm{structure}}_{j}+b{5}_{i}\times {\mathrm{current}}\,{\mathrm{woodland}}_{j}+b{6}_{i}\\\qquad\qquad\qquad\times\,{\mathrm{old}}\,{\mathrm{woodland}}_{j}+\,b{7}_{i}\times {\mathrm{lost}}\,{\mathrm{woodland}}_{j}+b{8}_{i}\\\qquad\qquad\qquad\times\,{\mathrm{trees}}\,{\mathrm{outside}}\,{\mathrm{woodlands}}_{j}+b{9}_{i}\times\, {\mathrm{arable}}_{j}+b{10}_{i}\\\qquad\qquad\qquad\times\,{\mathrm{livestock}}_{j}+b{11}_{i}\times {\mathrm{area}}_{j}\times {\mathrm{shape}}_{j}+b{12}_{i}\times\, {\mathrm{area}}_{j}\\\qquad\qquad\qquad\times\,{\mathrm{current}}\,{\mathrm{woodland}}_{j}+b{13}_{i}\times {\mathrm{area}}_{j}\times {\mathrm{arable}}_{j}+b{14}_{i}\\\qquad\qquad\qquad\times\, {\mathrm{shape}}_{j}\times {\mathrm{arable}}_{j}+b{15}_{i}\times {\mathrm{age}}_{j}\times {\mathrm{current}}\,{\mathrm{woodland}}_{j}\\\qquad\qquad\qquad\,+\,b{16}_{i}\times\, {\mathrm{trees}}\,{\mathrm{outside}}\,{\mathrm{woodlands}}_{j}\times {\mathrm{current}}\,{\mathrm{woodland}}_{j}\\\qquad\qquad\qquad\,+\,b{17}_{i}\times {\mathrm{country}}_{j}+{w}_{i}({s}_{j})\end{array}.$$

There are four site-scale variables: woodland site age, area, shape and structural heterogeneity; the next six variables are landscape-scale: the proportion of landscape that is currently woodland, the proportion of current woodland that is old, the proportion of landscape that is lost woodland, the proportion of trees outside woodlands, the proportion of agriculture that is arable, and livestock density. These ten variables represent either within-habitat-type spillovers, temporal legacies or between-habitat-type spillovers. The next six model terms were interactions, the final factor identified the country (that is, England or Scotland), and ${s}_{j}$ indicates the coordinates of the *j*th site, with ${w}_{i}({s}_{j})$ being the output of a zero-mean spatial Gaussian process^29^.

Modelling was conducted via R software^84^ using the package ‘spOccupancy’ (v0.7.6)^29^, using the spatial factor multispecies framework for the animal taxa (‘sfMsPGOcc’ function), and a spatial factor joint species distribution model for plants (‘sfJSDM’ function^30^). Therefore, all models accounted for potential spatial autocorrelation and correlated residuals among species, with the animal models also accounting for imperfect detection processes. In all cases, we used the minimally informative spOccupancy defaults for taxon-level hyperpriors for both detection and occupancy processes. Species-specific intercepts (that is, ${a0}_{i}$ and ${b0}_{i}$) were drawn from a normal distribution with mean of 0 and standard deviation of 2.7, and variance parameters used an inverse-gamma prior, with both shape and scale of 0.1 (ref. ^29^). Across four chains, we used 50,000 Markov chain Monte Carlo (MCMC) iterations for burn-in and generated 20,000 posterior samples at a thinning rate of 10. We confirmed chain convergence by visually assessing mixing in trace plots, ensuring reasonable effective sample sizes and checking all $\hat{R}$ values were <1.1 (ref. ^85^). We conducted posterior predictive checks to verify the goodness of fit for all models (Supplementary Information section 5). Plots showing species-level effects are presented in Supplementary Information section 6.

### Exploring spillover effects

We initially explored the marginal response of species-level occupancy to individual site- and landscape-scale variables, while holding all other predictors at their mean. We used model posterior predictions to simulate assemblages of each taxon for different combinations of spillover effects, exploring assemblage-level species richness and composition. For within-habitat-type spillovers, we considered woodland site area, the proportion of current woodland and the proportion of trees outside woodlands in the landscape. For temporal legacies we focused on woodland site age, the proportion of old woodland and the proportion of lost woodland in the landscape. For between-habitat-type spillover effects, we examined the agricultural intensity variables, the proportion of arable agriculture and livestock density, and their interactions with woodland site area as we assumed that this would mediate any effects. Finally, we consider all three drivers in combination, examining the effects of contemporary context (current woodland in the landscape and trees outside woodlands), historical landscape stability (proportions of both old woodland and lost woodland) and agricultural intensity (the proportion of arable agriculture and livestock density) on biodiversity in a hypothetical 3.5-ha woodland patch (the mean patch size in our dataset).

We used posterior estimates from the models to simulate assemblages of each taxon in response to the different landscape configurations in each of the three drivers. We generated 20,000 occupancy probabilities for each species using the species-level intercepts and coefficients from each posterior sample multiplied by systematic combinations of high and low values of each of the three variables in each driver. High and low values were selected on the basis of the ranges of the original predictor variables. To incorporate potential additional uncertainty, the remaining linear predictors were also included, with assigned values drawn from the marginal distributions in the original data. We converted these probabilities to presence–absence using Bernoulli trials. We compared the simulated spillover assemblages against assemblages from a ‘background’ landscape generated in the same way but using random values from the marginal distributions of all variables, to show how much the focal variables of each driver influenced richness and composition.

To examine patterns in assemblage composition, we used the ‘vegan’ R package (v2.5.7)^86^ to perform non-metric multidimensional scaling (NMDS) with three axes on a representative 10% of the simulated assemblages. We could only do this for beetles, birds and plants, as there were too few mammal species to use this approach.

### Ethics

All biodiversity surveys were conducted with approval from the University of Stirling’s ethics committee.

### Reporting summary

Further information on research design is available in the Nature Portfolio Reporting Summary linked to this article.

## Supplementary information

Supplementary Information Additional data collection details, methodology, supplementary analyses and supporting plots.

Reporting Summary

## Source data

Source Data Fig. 2 Statistical source data.

Source Data Fig. 3 Statistical source data.

Source Data Fig. 4 Statistical source data.

Source Data Extended Data Fig. 1 Statistical source data.

Source Data Extended Data Fig. 2 Statistical source data.

Source Data Extended Data Fig. 3 Statistical source data.

Source Data Extended Data Fig. 4 Statistical source data.

## Data Availability

All data used in this study are available via Zenodo at 10.5281/zenodo.14946190 (ref. ^87^). Source data are provided with this paper.

## Code availability

The R script for the analyses conducted in this study is available via Zenodo at 10.5281/zenodo.14946190 (ref. ^87^).
