# Supplementary material for: Spillovers and legacies of land management on temperate woodland biodiversity
Source: Nat Ecol Evol. 2025 Apr 23;9(6):1009–20. doi: 10.1038/s41559-025-02688-6 (PMC12148928; doi:10.1038/s41559-025-02688-6)
Supplement: Supplementary file 1 — Additional data collection details, methodology, supplementary analyses and supporting plots. [file 41559_2025_2688_MOESM1_ESM.pdf]

nature ecology & evolution

Supplementary information

https://doi.org/10.1038/s41559-025-02688-6

Spillovers and legacies of land management on temperate woodland biodiversity

In the format provided by the authors and unedited

Supplementary Information for Bradfer-Lawrence et al. (2025) "Spillovers and legacies of land management on temperate woodland biodiversity"

1. Biodiversity data

We used data from four taxa totalling 373 species: ground beetles (115 species), birds (54 species), small terrestrial mammals (4 species) and vascular plants (200 species). A full list of all species is in Table S1. Not all sites were surveyed for all taxa, see Figure S1. Where data collection covered two years, individual sites were only surveyed during a single year.

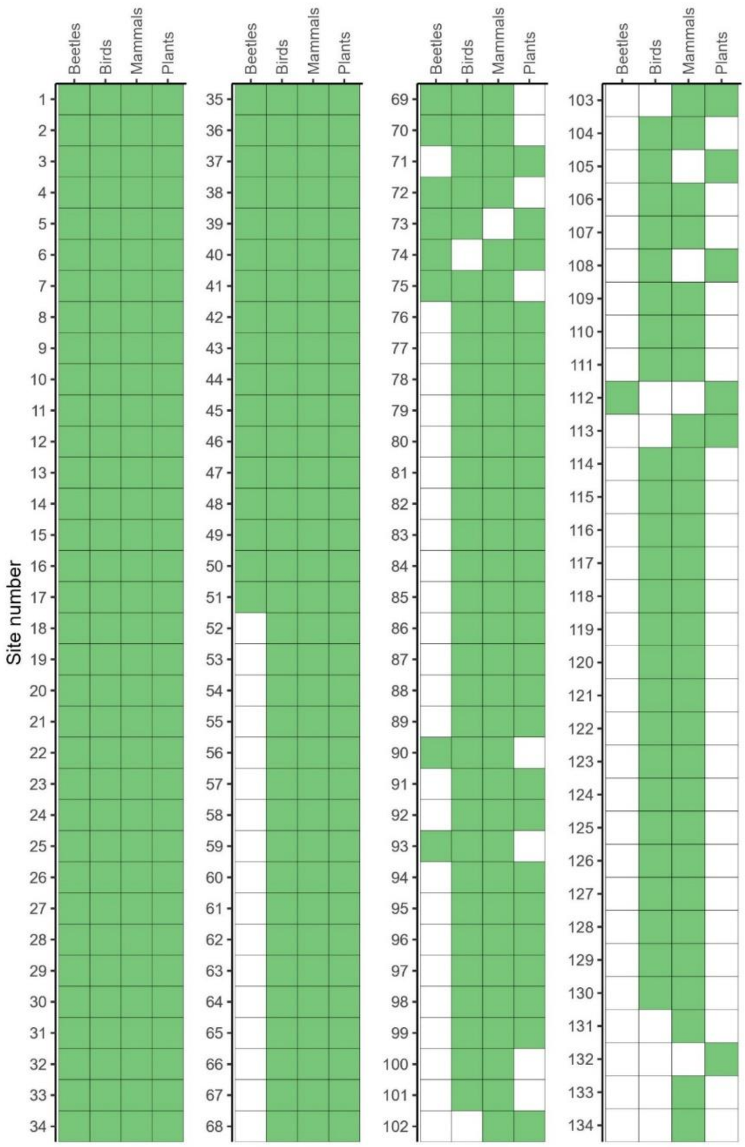

Figure S1. Site-by-survey matrix for 134 study sites, green squares indicate surveys were conducted for the taxon at that site.

1a. Beetles

Beetles (Coleoptera) were sampled using pitfall traps at 20 sites in July and August 2013, and at 40 sites between June and August 2014. These samples were originally intended for a study of edge effects, so traps were placed on a single linear transect running from the woodland edge to centre along the longest axis of each woodland. Number of traps was dictated by the length available for this transect, with between 7 and 15 traps per site. One trap was placed at the woodland edge, and with additional traps located progressively further into the woodland at 2m, 5m, 10m, and then at 25m intervals from 25m to 250m (or the centre of the wood, whichever was reached first).

Pitfall traps were deployed at each site for 10 weeks and checked fortnightly, giving five collections per site (except one site where only four collections could be undertaken). Traps were filled with 50% propylene glycol solution and covered with a metal lid to protect them from rainfall or other disturbances. After collection, invertebrates were stored in 70% ethanol. We excluded larvae and any specimens too damaged to identify. The remaining 27,302 individual beetles were identified to the lowest possible taxonomic level (163 to species, 3 to genus, 4 to family), with the latter two groups treated as morpho-species for this analysis.

Prior to analysis, we amalgamated site-level records across traps within each collection round. We removed singleton species (i.e., 55 species only detected at a single site), leaving 2,846 occupancy records for 115 species and morpho-species for analysis (Table S1, Figure S2). Although larger sites had more traps this was not directly proportional to site area, so we included number of traps as a covariate in the detectability portion of the model.

1b. Birds

Birds were surveyed at a total of 125 sites using Common Bird Census Methodology with a reduced number of surveys. Seventy-nine sites were visited once per month in April, May and June in 2015, and 46 sites were surveyed twice over the same period in 2017. Three experienced bird surveyors participated in the data collection but repeat visits to a site were always made by the same individual surveyor. To account for variations in site geometry, survey effort was standardized to 10 min per hectare per visit, with patches of <1 ha surveyed for a minimum of 10 min per visit. Surveys were conducted between 30 min after sunrise and

11:30. Surveyors walked within 50 m of all parts of each site to increase the probability of detection. All birds seen or heard were recorded, excluding individuals flying over the site or observed outside of the boundary. Further survey details can be found in Whytock et al (2018).

Prior to analysis we excluded seven species associated with aquatic habitats, (e.g., Dipper *Cinclus cinclus*). We assumed these birds were strongly influenced by the presence of water at some sites, confounding any associations with our predictors. We also excluded 11 singleton species, leaving 3,592 occupancy records of 54 species for analysis (Table S1, Figure S2). We included surveyor identity as a detectability covariate to account for potential individual differences in survey efficacy.

1c. Mammals

Small terrestrial mammals were live-trapped at 100 sites between June and August; at 31 sites in 2013 and 69 sites in 2014. We used Ugglan traps #2 (multi-catch wire mesh traps with roof covers; Grahnab, Sweden). At each site, 36 traps were deployed in a 9 x 4 grid spaced 10 m apart, with the grid as far as possible from the woodland edges. Traps were deployed for four continuous nights at each site and checked every morning. Traps were baited with food and bedding material was provided. Captured individuals were identified to species, temporarily marked by fur clipping to identify recaptures, and immediately released at the site of capture. Further survey details can be found in Fuentes-Montemayor et al (2020). We excluded recaptures and 3 singleton species, leaving 531 occupancy records of 4 species for analysis (Table S1, Figure S2).

1d. Plants

Vascular plant assemblages were assessed by two skilled surveyors, using a comprehensive walk over of 132 sites in 2015 and 2016 (Waddell et al 2024). Species were identified in situ, using a hand lens as necessary. With only a single visit per site, we had to assume perfect detectability, but some species may have been missed (Perret et al. 2023). Given most of the study woodlands were planted, we excluded tree species from the analysis as their presence is not necessarily reflective of site and landscape conditions (Verheyen et al. 2004). We also excluded 59 singleton species, leaving 3,507 occupancy records for 200 species for analysis (Table S1, Figure S2).

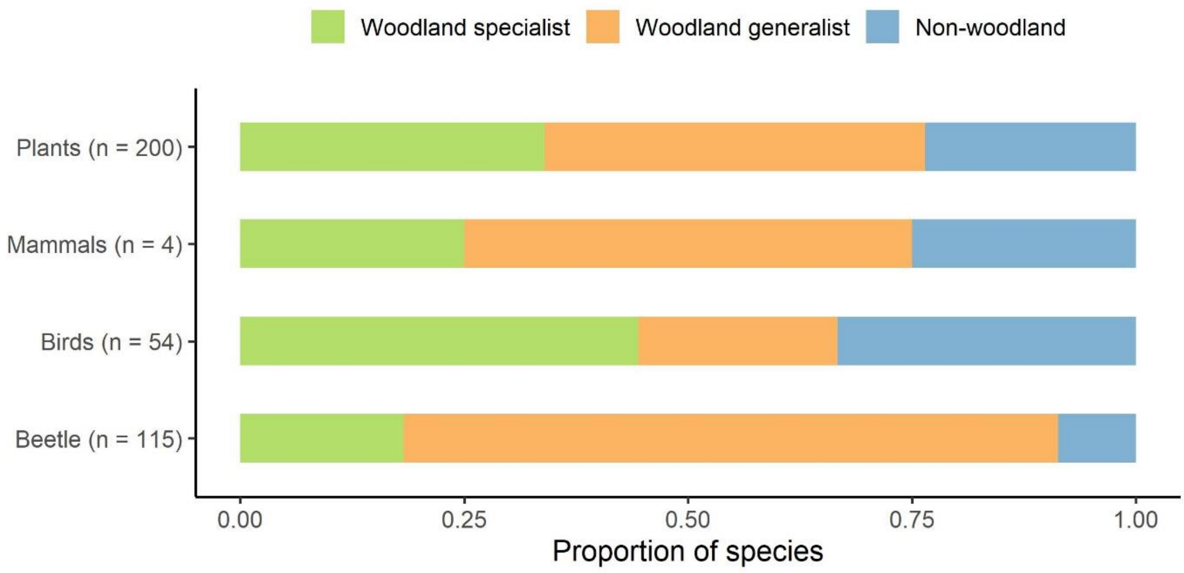

Figure S2. Summary of woodland affiliation distribution for 373 species in this study. 'Woodland specialists' are obligate or near-obligate species, 'woodland generalists' are species which often use woodlands but can persist in alternative habitats, 'non-woodland' species are those which are usually found in alternative habitats. Details for Beetles taken from Luff (1998, 2007); Birds from DEFRA (2024); Mammals from Fuentes Montemayor et al. (2020); Plants from Kirby et al. (2012).

Table 1. List of 373 species included in this study with woodland affiliation details; definitions and sources as per Figure S2.

**Taxon**	**Species**	**Woodland affiliation**
Beetles	Abax parallelepipedus	Generalist
Beetles	Acidota cruentata	Specialist
Beetles	Agonum emarginatum	Non-woodland
Beetles	Agonum fuliginosum	Non-woodland
Beetles	Agonum gracile	Non-woodland
Beetles	Agonum micans	Generalist
Beetles	Agriotes lineatus	Generalist
Beetles	Agriotes obscurus	Non-woodland
Beetles	Agriotes sputator	Generalist
Beetles	Aleocharinae	Generalist
Beetles	Amara eurynota	Non-woodland
Beetles	Anchomenus dorsalis	Non-woodland
Beetles	Anotylus inustus	Non-woodland
Beetles	Anotylus rugosus	Generalist

Beetles	Anotylus sculpturatus	Non-woodland
Beetles	Aphodius depressus	Generalist
Beetles	Aphodius rufipes	Generalist
Beetles	Athous haemorrhoidalis	Generalist
Beetles	Badister bullatus	Non-woodland
Beetles	Bembidion aeneum	Generalist
Beetles	Bembidion lampros	Non-woodland
Beetles	Bembidion mannerheimii	Generalist
Beetles	Bisnius fimetarius	Generalist
Beetles	Calathus fuscipes	Non-woodland
Beetles	Calathus melanocephalus	Non-woodland
Beetles	Calathus rotundicollis	Generalist
Beetles	Carabus nemoralis	Generalist
Beetles	Carabus problematicus	Generalist
Beetles	Carabus violaceus	Generalist
Beetles	Cartodere nodifer	Generalist
Beetles	Choleva jeanneli	Generalist
Beetles	Clivina fossor	Non-woodland
Beetles	Cychrus caraboides	Generalist
Beetles	Exomias araneiformis	Generalist
Beetles	Exomias pellucidus	Generalist
Beetles	Gastrophysa polygoni	Generalist
Beetles	Geotrupes stercorarius	Generalist
Beetles	Harpalus affinis	Non-woodland
Beetles	Harpalus rufipes	Non-woodland
Beetles	Hemicrepidius hirtus	Generalist
Beetles	Hypnoidus riparius	Non-woodland
Beetles	Lathrobium brunnipes	Generalist
Beetles	Lathrobium elongatum	Generalist
Beetles	Lathrobium fulvipenne	Generalist
Beetles	Leistus fulvibarbis	Generalist
Beetles	Leistus rufomarginatus	Specialist
Beetles	Leistus terminatus	Generalist
Beetles	Lesteva sicula heeri	Non-woodland
Beetles	Loricera pilicornis	Generalist
Beetles	Megasternum concinnum	Generalist
Beetles	Nebria brevicollis	Generalist
Beetles	Nicrophorus humator	Generalist
Beetles	Nicrophorus investigator	Generalist
Beetles	Nicrophorus vespilloides	Generalist
Beetles	Notiophilus biguttatus	Generalist
Beetles	Ocypus aeneocephalus	Generalist
Beetles	Ocypus brunnipes	Generalist
Beetles	Ocypus olens	Generalist
Beetles	Omaliinae	Generalist
Beetles	Othius angustus	Generalist

Beetles	Othius laeviusculus	Generalist
Beetles	Othius punctulatus	Specialist
Beetles	Othius subuliformis	Generalist
Beetles	Otiorhynchus nodosus	Specialist
Beetles	Otiorhynchus singularis	Generalist
Beetles	Oxytelus laqueatus	Generalist
Beetles	Paranchus albipes	Generalist
Beetles	Patrobus atrorufus	Generalist
Beetles	Philonthus cognatus	Generalist
Beetles	Philonthus decorus	Generalist
Beetles	Philonthus laminatus	Generalist
Beetles	Philonthus mannerheimi	Generalist
Beetles	Philonthus marginatus	Generalist
Beetles	Philonthus tenuicornis	Generalist
Beetles	Platynus assimilis	Specialist
Beetles	Pterostichus madidus	Generalist
Beetles	Pterostichus melanarius	Non-woodland
Beetles	Pterostichus minor	Non-woodland
Beetles	Pterostichus niger	Generalist
Beetles	Pterostichus nigrata	Generalist
Beetles	Pterostichus nigrata rhaeticus	Non-woodland
Beetles	Pterostichus strenuus	Generalist
Beetles	Quedius curtipennis	Generalist
Beetles	Quedius fuliginosus	Generalist
Beetles	Quedius fumatus	Specialist
Beetles	Quedius invreae	Generalist
Beetles	Quedius lateralis	Specialist
Beetles	Quedius levicollis	Generalist
Beetles	Quedius mesomelinus	Generalist
Beetles	Quedius molochinus	Specialist
Beetles	Quedius nitipennis	Generalist
Beetles	Quedius puncticollis	Generalist
Beetles	Rhagonycha nigriventris	Generalist
Beetles	Rhizophagus dispar	Specialist
Beetles	Rugilus rufipes	Generalist
Beetles	Serica brunnea	Generalist
Beetles	Silpha atrata	Generalist
Beetles	Staphylinus erythropterus	Generalist
Beetles	Stenichnus collaris	Specialist
Beetles	Stenus bimaculatus	Generalist
Beetles	Stenus brunnipes	Generalist
Beetles	Stenus clavicornis	Generalist
Beetles	Stenus juno	Generalist
Beetles	Stenus lustrator	Generalist
Beetles	Synuchus vivalis	Generalist
Beetles	Tachinus humeralis	Generalist

Beetles	Tachinus marginellus	Generalist
Beetles	Tachinus rufipes	Generalist
Beetles	Tachyporinae	Generalist
Beetles	Tasgius morsitans	Generalist
Beetles	Trechus obtusus	Non-woodland
Beetles	Trechus secalis	Generalist
Beetles	Xantholinus elegans	Generalist
Beetles	Xantholinus linearis	Generalist
Beetles	Xantholinus longiventris	Generalist
Birds	Acanthis cabaret	Specialist
Birds	Accipiter nisus	Specialist
Birds	Acrocephalus schoenobaenus	Non-woodland
Birds	Aegithalos caudatus	Generalist
Birds	Anthus trivialis	Specialist
Birds	Buteo buteo	Non-woodland
Birds	Carduelis carduelis	Non-woodland
Birds	Certhia familiaris	Specialist
Birds	Cettia cetti	Non-woodland
Birds	Chloris chloris	Non-woodland
Birds	Columba oenas	Non-woodland
Birds	Columba palumbus	Non-woodland
Birds	Corvus corone	Non-woodland
Birds	Corvus frugilegus	Non-woodland
Birds	Corvus monedula	Non-woodland
Birds	Cuculus canorus	Non-woodland
Birds	Cyanistes caeruleus	Generalist
Birds	Dendrocopos major	Specialist
Birds	Emberiza citrinella	Non-woodland
Birds	Emberiza schoeniclus	Non-woodland
Birds	Erithacus rubecula	Generalist
Birds	Fringilla coelebs	Generalist
Birds	Garrulus glandarius	Specialist
Birds	Linaria cannabina	Non-woodland
Birds	Milvus milvus	Non-woodland
Birds	Motacilla alba	Non-woodland
Birds	Muscicapa striata	Specialist
Birds	Parus major	Generalist
Birds	Passer domesticus	Non-woodland
Birds	Passer montanus	Non-woodland
Birds	Periparus ater	Specialist
Birds	Phasianus colchicus	Non-woodland
Birds	Phoenicurus phoenicurus	Specialist
Birds	Phylloscopus collybita	Specialist
Birds	Phylloscopus trochilus	Specialist
Birds	Pica pica	Non-woodland

Birds	Picus viridis	Specialist
Birds	Poecile palustris	Specialist
Birds	Prunella modularis	Generalist
Birds	Pyrrhula pyrrhula	Generalist
Birds	Regulus regulus	Specialist
Birds	Sitta europaea	Specialist
Birds	Spinus spinus	Specialist
Birds	Strix aluco	Generalist
Birds	Sturnus vulgaris	Non-woodland
Birds	Sylvia atricapilla	Specialist
Birds	Sylvia borin	Specialist
Birds	Sylvia communis	Non-woodland
Birds	Sylvia curruca	Generalist
Birds	Troglodytes troglodytes	Generalist
Birds	Turdus merula	Generalist
Birds	Turdus philomelos	Generalist
Birds	Turdus pilaris	Non-woodland
Birds	Turdus viscivorus	Non-woodland
Mammals	Apodemus sylvaticus	Generalist
Mammals	Microtus agrestis	Non-woodland
Mammals	Myodes glareolus	Specialist
Mammals	Sorex araneus	Generalist
Plants	Achillea millefolium	Non-woodland
Plants	Aegopodium podagraria	Generalist
Plants	Agrimonia eupatoria	Non-woodland
Plants	Agrostis capillaris	Generalist
Plants	Agrostis gigantea	Generalist
Plants	Agrostis stolonifera	Generalist
Plants	Ajuga reptans	Generalist
Plants	Alliaria petiolata	Generalist
Plants	Allium ursinum	Specialist
Plants	Alopecurus pratensis	Non-woodland
Plants	Anemone nemorosa	Specialist
Plants	Angelica officinalis	Non-woodland
Plants	Angelica sylvestris	Generalist
Plants	Anisantha sterilis	Non-woodland
Plants	Anthoxanthum odoratum	Generalist
Plants	Anthriscus sylvestris	Generalist
Plants	Arctium minus	Generalist
Plants	Arrhenatherum elatius	Generalist
Plants	Arum maculatum	Specialist
Plants	Athyrium filix-femina	Specialist
Plants	Atropa belladonna	Generalist
Plants	Bellis perennis	Non-woodland

Plants	Blechnum spicant	Specialist
Plants	Brachypodium sylvaticum	Specialist
Plants	Brassica napus	Non-woodland
Plants	Bromopsis ramosa	Specialist
Plants	Bromus hordeaceus	Non-woodland
Plants	Bryonia dioica	Generalist
Plants	Calluna vulgaris	Generalist
Plants	Caltha palustris	Generalist
Plants	Calystegia sepium	Generalist
Plants	Campanula rotundifolia	Generalist
Plants	Cardamine flexuosa	Generalist
Plants	Cardamine hirsuta	Non-woodland
Plants	Cardamine pratensis	Generalist
Plants	Carduus nutans	Non-woodland
Plants	Carex flacca	Non-woodland
Plants	Carex pendula	Specialist
Plants	Carex sylvatica	Specialist
Plants	Centaurea nigra	Non-woodland
Plants	Cerastium arvense	Non-woodland
Plants	Cerastium fontanum	Non-woodland
Plants	Ceratocapnos claviculata	Specialist
Plants	Chamerion angustifolium	Generalist
Plants	Chrysosplenium oppositifolium	Specialist
Plants	Circaea lutetiana	Generalist
Plants	Cirsium arvense	Non-woodland
Plants	Cirsium palustre	Generalist
Plants	Cirsium vulgare	Generalist
Plants	Claytonia sibirica	Generalist
Plants	Conopodium majus	Specialist
Plants	Crepis biennis	Non-woodland
Plants	Crepis paludosa	Generalist
Plants	Cruciata laevipes	Non-woodland
Plants	Cynosurus cristatus	Non-woodland
Plants	Cytisus scoparius	Generalist
Plants	Dactylis glomerata	Generalist
Plants	Dactylorhiza fuchsii	Generalist
Plants	Daucus carota	Non-woodland
Plants	Deschampsia cespitosa	Generalist
Plants	Deschampsia flexuosa	Generalist
Plants	Digitalis purpurea	Generalist
Plants	Dryopteris affinis	Specialist
Plants	Dryopteris dilatata	Generalist
Plants	Dryopteris filix-mas	Generalist
Plants	Epilobium ciliatum	Non-woodland
Plants	Epilobium hirsutum	Generalist
Plants	Epilobium montanum	Generalist

Plants	Epilobium tetragonum	Generalist
Plants	Equisetum arvense	Non-woodland
Plants	Fallopia japonica	Non-woodland
Plants	Festuca ovina	Generalist
Plants	Festuca pratensis	Non-woodland
Plants	Festuca rubra	Generalist
Plants	Filipendula ulmaria	Generalist
Plants	Fragaria vesca	Specialist
Plants	Galeopsis tetrahit	Generalist
Plants	Galium aparine	Generalist
Plants	Galium odoratum	Specialist
Plants	Galium palustre	Generalist
Plants	Galium saxatile	Generalist
Plants	Geranium dissectum	Non-woodland
Plants	Geranium pusillum	Non-woodland
Plants	Geranium robertianum	Specialist
Plants	Geum rivale	Specialist
Plants	Geum urbanum	Generalist
Plants	Glechoma hederacea	Generalist
Plants	Hedera helix	Generalist
Plants	Heracleum mantegazzianum	Non-woodland
Plants	Heracleum sphondylium	Generalist
Plants	Holcus lanatus	Generalist
Plants	Holcus mollis	Specialist
Plants	Hyacinthoides hispanica	Non-woodland
Plants	Hyacinthoides non-scripta	Specialist
Plants	Hypericum perforatum	Generalist
Plants	Hypericum pulchrum	Specialist
Plants	Hypochaeris radicata	Non-woodland
Plants	Impatiens glandulifera	Non-woodland
Plants	Iris pseudacorus	Generalist
Plants	Juncus articulatus	Non-woodland
Plants	Juncus conglomeratus	Generalist
Plants	Juncus effusus	Generalist
Plants	Juncus inflexus	Non-woodland
Plants	Lamiastrum galeobdolon	Specialist
Plants	Lamium album	Generalist
Plants	Lamium purpureum	Non-woodland
Plants	Lapsana communis	Generalist
Plants	Lathyrus pratensis	Non-woodland
Plants	Leontodon hispidus	Non-woodland
Plants	Lolium perenne	Non-woodland
Plants	Lonicera periclymenum	Specialist
Plants	Lotus corniculatus	Non-woodland
Plants	Lotus pedunculatus	Non-woodland
Plants	Luzula sylvatica	Specialist

Plants	Lysimachia nemorum	Specialist
Plants	Meconopsis cambrica	Generalist
Plants	Melica uniflora	Specialist
Plants	Mentha aquatica	Generalist
Plants	Mercurialis perennis	Specialist
Plants	Molinia caerulea	Generalist
Plants	Myosotis arvensis	Generalist
Plants	Myosotis sylvatica	Specialist
Plants	Narcissus pseudonarcissus	Specialist
Plants	Oxalis acetosella	Specialist
Plants	Persicaria hydropiper	Non-woodland
Plants	Petasites albus	Non-woodland
Plants	Petasites hybridus	Generalist
Plants	Phalaris arundinacea	Non-woodland
Plants	Phleum pratense	Non-woodland
Plants	Phragmites australis	Non-woodland
Plants	Picris echioides	Non-woodland
Plants	Plantago lanceolata	Non-woodland
Plants	Plantago major	Non-woodland
Plants	Plantago media	Non-woodland
Plants	Poa annua	Generalist
Plants	Poa pratensis sens lat	Non-woodland
Plants	Poa trivialis	Generalist
Plants	Polygonatum multiflorum	Specialist
Plants	Polygonum aviculare	Non-woodland
Plants	Polystichum setiferum	Specialist
Plants	Potentilla anserina	Generalist
Plants	Potentilla erecta	Generalist
Plants	Potentilla reptans	Generalist
Plants	Potentilla sterilis	Specialist
Plants	Primula vulgaris	Specialist
Plants	Prunella vulgaris	Generalist
Plants	Pteridium aquilinum	Generalist
Plants	Ranunculus acris	Non-woodland
Plants	Ranunculus ficaria	Generalist
Plants	Ranunculus repens	Generalist
Plants	Ranunculus sardous	Non-woodland
Plants	Ribes rubrum	Specialist
Plants	Ribes uva-crispa	Generalist
Plants	Rosa arvensis	Specialist
Plants	Rosa canina agg	Generalist
Plants	Rubus fruticosus	Generalist
Plants	Rubus idaeus	Generalist
Plants	Rumex acetosa	Generalist
Plants	Rumex alpinus	Non-woodland
Plants	Rumex conglomeratus	Generalist

Plants	Rumex longifolius	Non-woodland
Plants	Rumex obtusifolius	Generalist
Plants	Rumex sanguineus	Generalist
Plants	Sanicula europaea	Specialist
Plants	Saxifraga granulata	Non-woodland
Plants	Saxifraga oppositifolia	Non-woodland
Plants	Scrophularia nodosa	Specialist
Plants	Senecio erucifolius	Non-woodland
Plants	Senecio jacobaea	Non-woodland
Plants	Silene dioica	Specialist
Plants	Solanum dulcamara	Generalist
Plants	Sonchus oleraceus	Non-woodland
Plants	Stachys sylvatica	Specialist
Plants	Stellaria graminea	Generalist
Plants	Stellaria holostea	Specialist
Plants	Stellaria media	Generalist
Plants	Stellaria nemorum	Specialist
Plants	Symphytum officinale	Non-woodland
Plants	Taraxacum	Generalist
Plants	Teucrium scorodonia	Generalist
Plants	Torilis japonica	Generalist
Plants	Trifolium pratense	Non-woodland
Plants	Trifolium repens	Non-woodland
Plants	Triticum aestivum	Non-woodland
Plants	Typha latifolia	Non-woodland
Plants	Ulex europaeus	Generalist
Plants	Urtica dioica	Generalist
Plants	Vaccinium myrtillus	Specialist
Plants	Veronica anagallis-aquatica	Non-woodland
Plants	Veronica beccabunga	Non-woodland
Plants	Veronica chamaedrys	Generalist
Plants	Veronica montana	Specialist
Plants	Veronica serpyllifolia	Non-woodland
Plants	Vicia cracca	Non-woodland
Plants	Vicia sativa	Non-woodland
Plants	Vicia sepium	Specialist
Plants	Vicia tetrasperma	Non-woodland
Plants	Viola odorata	Specialist
Plants	Viola palustris	Specialist
Plants	Viola riviniana	Specialist

2. Predictor preparation

2a. Site-level predictors

Ancient woodlands were identified using national Ancient Woodland Inventories (Natural England 2023; Scottish Government 2023). We used historical Ordnance Survey mapping to determine age of planted sites. We assumed that the woodland was planted during the map's publication year although there is likely to have been a delay between planting and the woodland's appearance on the map (Watts et al. 2016).

Woodland site area and shape were derived from National Forest Inventory mapping (NFI, FR 2018) using GIS. Shape was the ratio of site perimeter divided by the perimeter of a circle with the same area; larger values therefore indicate less-compact sites. We used the standard deviation of tree DBH in cms collected during vegetation surveys as a measure of woodland structural heterogeneity. Full vegetation survey details are in Fuentes-Montemayor et al. (2022).

2b. Landscape-level predictors – within-habitat-type

We defined the landscape as the 3 km radius around each site, and calculated four within-habitat-type landscape-scale predictors. We identified all woodlands greater than 0.5 ha in size at four points in time (1920s, 1950s, 1990s, and 2015). The 1920s and 1950s data were assembled from historical Ordnance Survey maps using a custom workflow in R and QGIS that allowed us to extract areas of woodland on the basis of colour. Although the Ordnance Survey extends back to the 1860s, the earliest comprehensive colour maps only appeared in the 1920s, hence our choice of baseline. The 1990s data came from Forest Research's National Inventory of Woodlands and Trees (Smith et al. 2010). The 2015 data were extracted from the National Forest Inventory (FR 2018).

From these four woodland layers we calculated three measures of woodland cover. Current woodland was the proportion of the 3 km radius landscape with woodland in 2015. Old woodland was the proportion of the current woodland that had been present at all four points in time (and was therefore at least 100 years old). This was achieved by stacking the four layers together and identifying the 5 m^2 pixels that were woodland in all time points. Lost woodland was the proportion of the landscape that had been wooded during at least one of the historic time points, but was not woodland in 2015.

As matrix permeability plays a key role in dispersal (Hinsley & Bellamy 2000; Vanneste et al 2020), we supplemented the woodland cover predictors with a 'trees outside woodlands' dataset for 2015 (Forest Research 2021). This was created using a combination of LiDAR and photogrammetry to identify woodlands smaller than 0.5 ha (and therefore absent from the NFI), linear hedgerows and isolated trees. We used proportion of the landscape covered with trees outside woodlands as our predictor.

2c. Landscape-level predictors – between-habitat-type

We created two measures of agricultural intensity for 3 km radius landscapes around each woodland site. Using LCM2015 data (Rowland et al 2017), we calculated the proportion of all agricultural land (categories 'Arable', 'Improved grassland', 'Semi-natural grassland') that was 'Arable'.

AgCensus (EDINA 2022) maps a range of agricultural information derived from the UK's annual June Agricultural Census. The most recent data available for both Scotland and England are from 2010, at 2 km^2 and 5 km^2 resolution respectively. We converted numbers of cattle and sheep in each pixel to Livestock Units (LSU) using information from Eurostat. Dairy cattle are 1 LSU, with other animals scaled relative to this according to dietary requirements. For each pixel we divided the total LSUs by the area of grassland in ha. Grassland area was derived by combining 'Grassland < 5 years old', 'Grassland > 5 years old' and 'Rough grazing' information from AgCensus. Although slightly different to the LCM2015 grassland values derived above (r = 0.93), this had the benefit of guaranteeing spatial congruence between livestock and grassland data.

3. Correlations across landscape scales

Apparent relationships between biodiversity and landscape composition can be influenced by the size of radius chosen to represent the 'landscape-scale' (Jackson & Fahrig 2014). However, beyond 1 km radius our study landscapes are largely homogenous, showing high correlations (> 0.7) across scales. We extracted landcover data from the LCM2015 dataset (Rowland et al 2017) as proportions of a series of radii between 1 – 3 km, for woodlands, and agriculture (arable and intensive grasslands combined), see Figures S3 and S4.

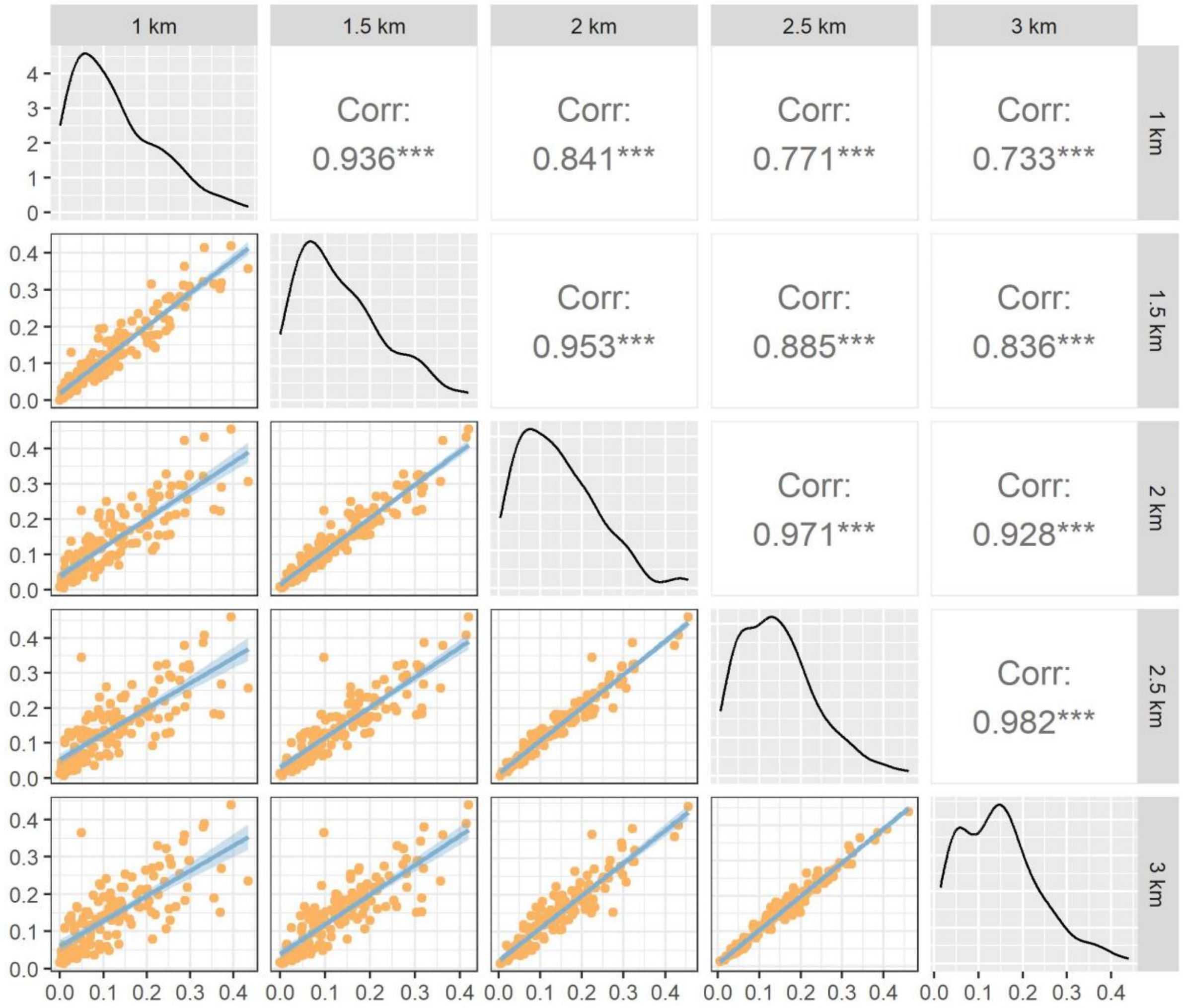

Figure S3. Correlations between proportions of the landscape covered in woodland across a range of spatial scales surrounding study sites, from LCM2015 data.

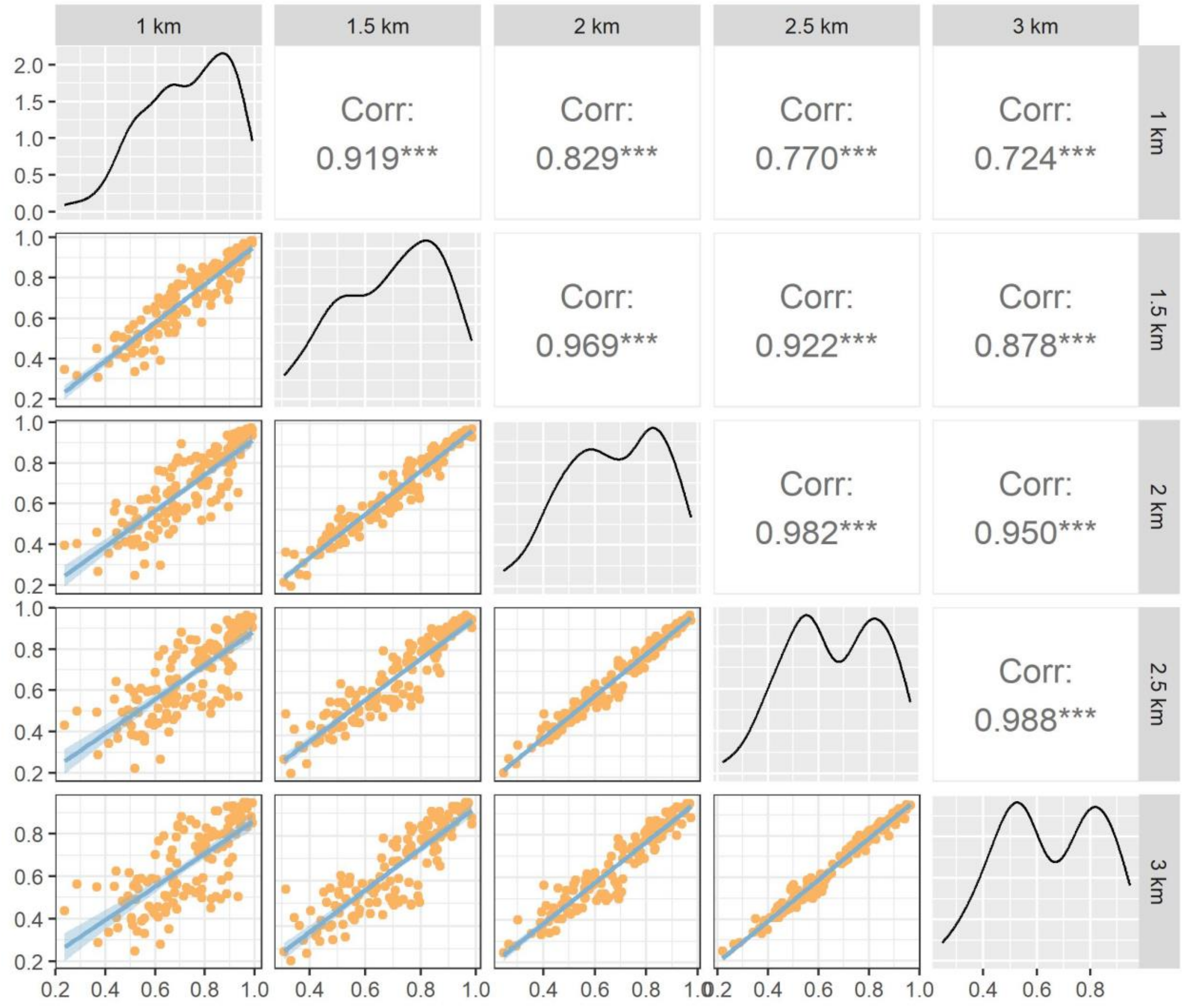

Figure S4. Correlations between proportions of the landscape covered in agriculture (arable and intensive grassland) across a range of spatial scales surrounding study sites, from LCM2015 data.

4. Excluded predictor variables

During study design we considered but discarded several additional predictor variables.

4a. Roads

Roads are known to effect wildlife distributions, particularly for vertebrate species (e.g., Benitez-Lopez et al. 2010; Cooke et al. 2020a & b). We extracted road length from Ordnance Survey mapping for the 3 km radius landscapes. Among sites there was some variation in road density (m per ha), but this covaried strongly with area of urban landcover from LCM 2015 data (correlation 0.74, Figure S5). As urban landcover types are the second

most prevalent after agriculture in our study landscapes, any increase in urban area (and therefore road density) is associated with lower amounts of agriculture. We therefore excluded roads from our potential set of predictors, while acknowledging it leaves this influence on wildlife distributions unaccounted for.

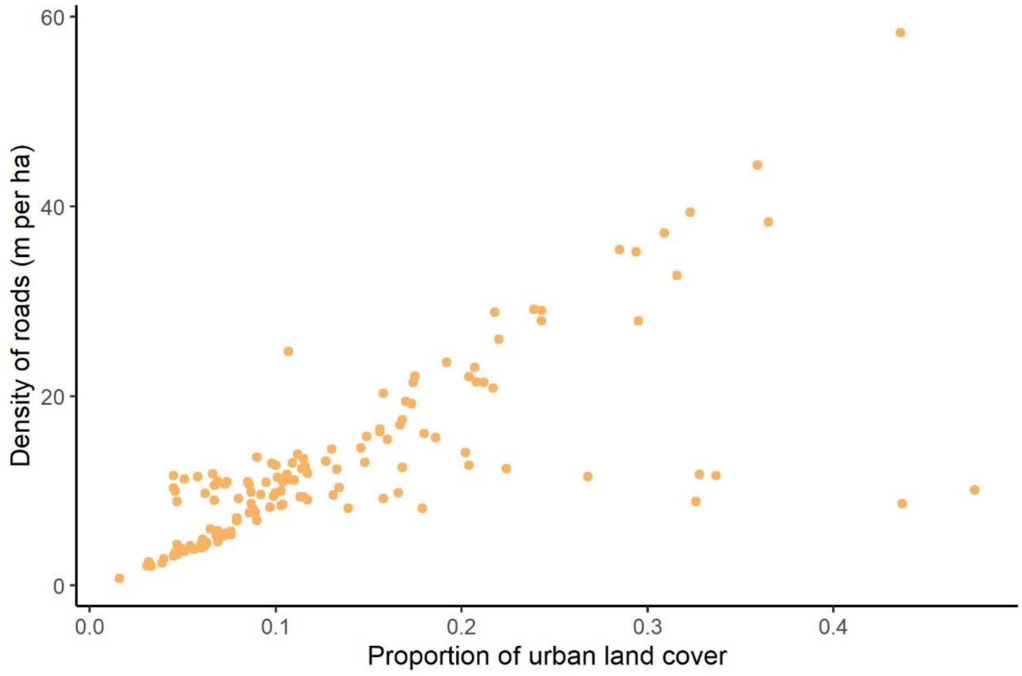

Figure S5. Correlation between proportion of urban land cover and density of roads.

4b. Agricultural inputs

Agricultural intensification, and in particular the widespread application of pesticides and fertilisers, has caused major population declines in a wide range of taxa (e.g., Frampton & Dorne 2007 for invertebrates; Li et al. 2020, Rigal et al. 2023 for birds). We explored the 1km^2-resolution 'CEH Land Cover plus' datasets as potential predictors reflecting agricultural management intensity (CEH 2020). At present, data for fertilisers are only available for England not Scotland, so we discounted this. The pesticide dataset combines mean annual usage of 162 pesticides for 2012-2017 with detailed crop maps for all of Britain. To account for the potential disconnect between the amount of pesticide used and its impact on wildlife, we multiplied the weight applied by a measure of ecotoxicity for each active ingredient. Ecotoxicity information was collated from the Pesticide Properties Database (Lewis et al. 2016) and combined following Kudsk et al. (2018), incorporating eight

measures of acute toxicity (for mammals, birds, fish, daphnia, algae, aquatic plants, earthworms and bees), and three measures of chronic toxicity (for fish, daphnia and earthworms). However, the resulting ecotoxicity map was highly correlated (r = 0.99) with our measure of arable land as a proportion of agriculture and hence we opted not to include the pesticide predictor in our models.

4c. Temporal trends in between-habitat-type predictors

Between-habitat-type temporal legacies reflect the influence of changing agricultural management on nearby woodland patches, which are potentially important drivers in determining contemporary distributions of species. Although there are studies of such between-habitat-type temporal legacies at national scales (e.g., Chamberlain et al. 2000; Robinson & Sutherland 2002), there is little suitable data at a sufficiently fine spatial resolution which we could include in our analysis. The Agcensus data does have patchy coverage extending back to 1969 (EDINA 2021), which we used to generate decadal trends in proportion of arable cropland and grazing livestock density using the methods described in SI section 2c. While intensity fluctuated over time, values were tightly correlated across sites so that their relative ranked positions did not change; landscapes with high agricultural intensity in the 1960s continued to have high intensity management through to the present day (proportion of arable cropland correlations 0.89 – 0.97; livestock density 0.49 – 0.87; Figures S6 and S7). It is likely that agricultural intensification occurred at different times among our study sites, so that the legacies are at different stages and may be influencing woodland biodiversity. However, we are unable to reflect these changes in our models, and so opted to only retain our between-habitat-type spatial predictors for our analysis.

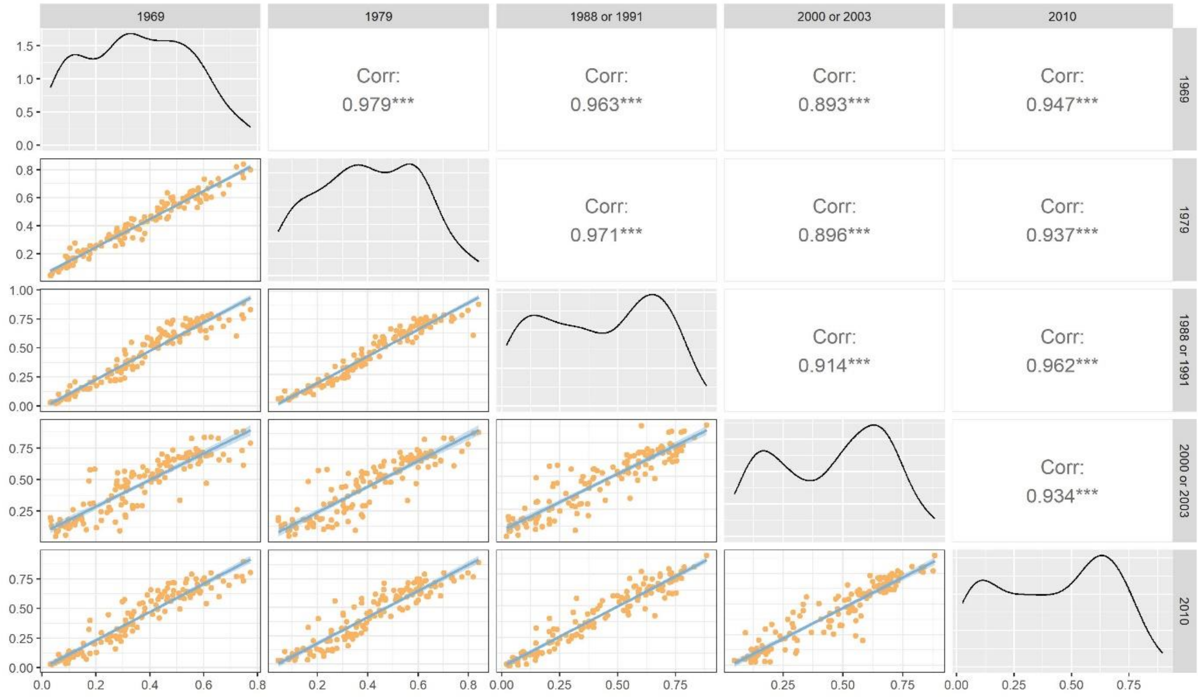

Figure S6. Correlations across time in proportion of arable cropland in 3-km radius landscapes surrounding our study sites. Agcensus data for England and Scotland is available for 1969, 1979 and 2010. Data is only available for Scotland in 1991 and 2000, and only for England in 1988 and 2003.

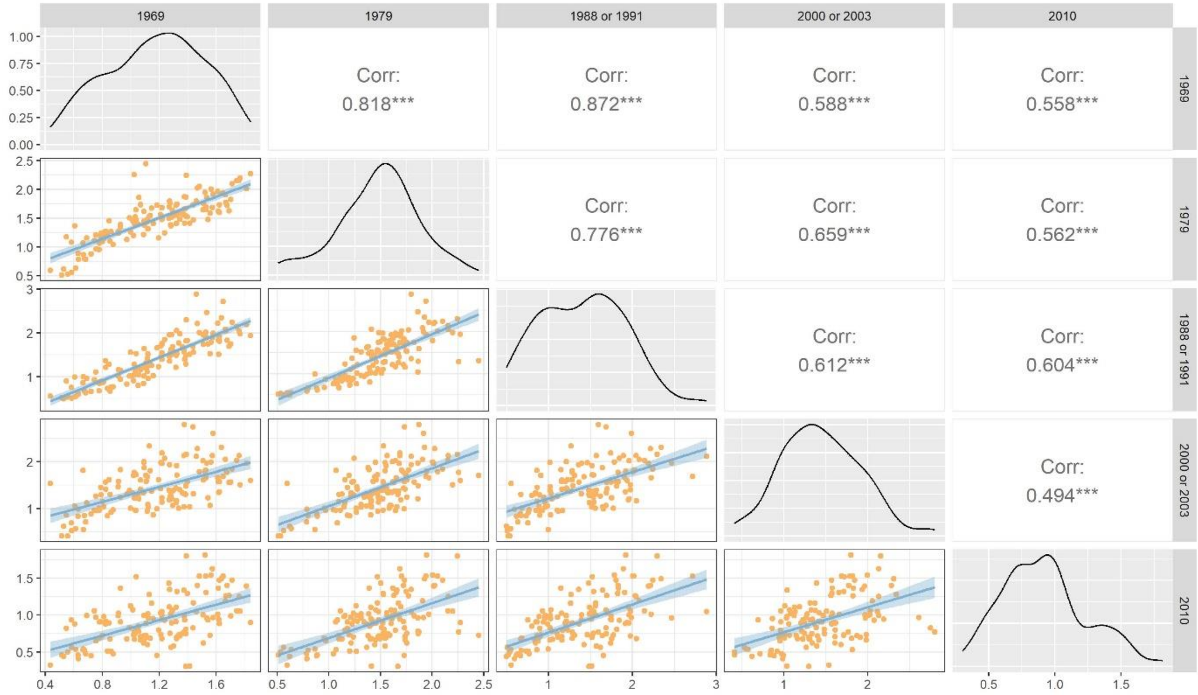

Figure S7. Correlations across time in livestock density (as Livestock Units) in 3-km radius landscapes surrounding our study sites. Agcensus data for England and Scotland is available

for 1969, 1979 and 2010. Data is only available for Scotland in 1991 and 2000, and only for England in 1988 and 2003.

5. Posterior Predictive Checks

We undertook Goodness of Fit tests to confirm that all models were capable of simulating data similar to the original data. For the animal taxa, this was done via the spOccupancy package 'ppcOcc' function, testing with both chi-squared and Freeman-Tukey statistics and combining across either sites or replicates (Doser et al 2022). This function is not available for sfJSDM outputs, so we manually conducted similar tests for plants. In all cases, the assemblage level Bayesian p values were between 0.1 – 0.5 suggesting an adequate model fit. For individual species, 786 of 892 (88%) of all possible species by site or species by replicate tests were satisfactory. We deemed this to be acceptable given that we were concerned with assemblage-level responses, and propagated full uncertainty from the models throughout subsequent analyses, so that any inaccuracies would be equally imprecise across all outputs.

6. Species-level effects

Plots below show species-level effects for the 10 linear predictors for each of the four taxa. Thick bars show central 50% of the posterior, whiskers show 95% credible intervals. Red bars indicate when credible intervals do not include zero, grey bars when they overlap with zero.

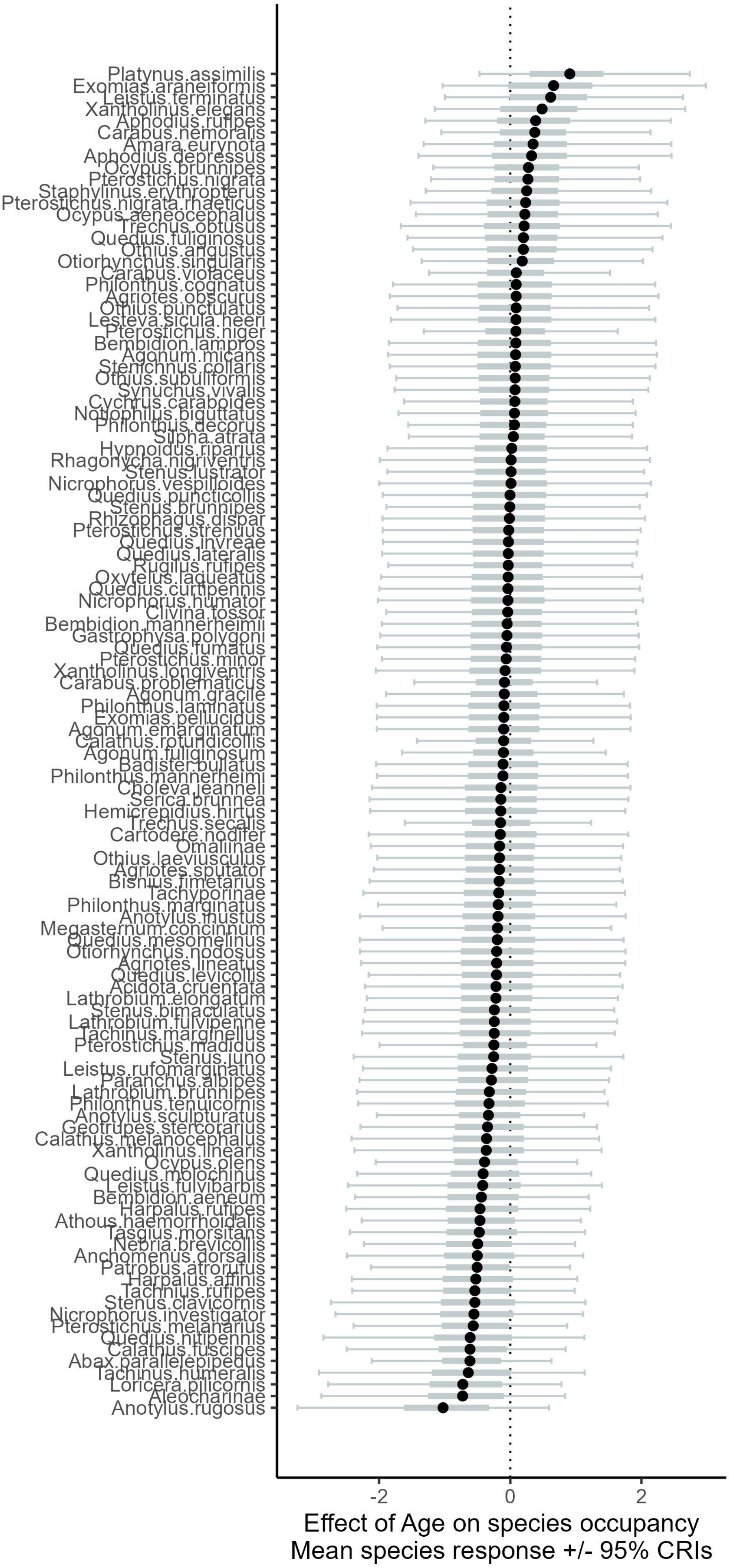
Platynus.assimilis
Exomias.araneiformis
Leistus.terminatus
Xantholinus.elegans
Aphodius.rufipes
Carabus.nemoralis
Amara.eurynota
Aphodius.depressus
Ocypus.brunnipes
Pterostichus.nigrata
Staphylinus.erythropterus
Pterostichus.nigrata.rhaeticus
Ocypus.aeneocephalus
Trechus.obtusus
Quedius.fuliginosus
Othius.angustus
Otiorhynchus.singularis
Carabus.violaceus
Philonthus.cognatus
Agriotes.obscurus
Othius.punctulatus
Lesteva.sicula.heeri
Pterostichus.niger
Bembidion.lampros
Agonum.micans
Stenichnus.collaris
Othius.subuliformis
Synuchus.vivalis
Cychrus.caraboides
Notiophilus.biguttatus
Philonthus.decorus
Silpha.atrata
Hypnoidus.riparius
Rhagonycha.nigriventris
Stenus.lustrator
Nicrophorus.vespilloides
Quedius.puncticollis
Stenus.brunnipes
Rhizophagus.dispar
Pterostichus.strenuus
Quedius.invreae
Quedius.lateralis
Rugilus.rufipes
Oxytelus.laqueatus
Quedius.curtipennis
Nicrophorus.humator
Clivina.fossor
Bembidion.mannerheimii
Gastrophysa.polygoni
Quedius.fumatus
Pterostichus.minor
Xantholinus.longiventris
Carabus.problematicus
Agonum.gracile
Philonthus.laminatus
Exomias.pellucidus
Agonum.emarginatum
Calathus.rotundicollis
Agonum.fuliginosum
Badister.bullatus
Philonthus.mannerheimi
Choleva.jeanneli
Serica.brunnea
Hemicrepidius.hirtus
Trechus.secalis
Cartodere.nodifer
Omaliinae
Othius.laeviusculus
Agriotes.sputator
Bisnius.fimetarius
Tachyporinae
Philonthus.marginatus
Anotylus.inustus
Megasternum.concinnum
Quedius.mesomelinus
Otiorhynchus.nodosus
Agriotes.lineatus
Quedius.levicollis
Acidota.cruentata
Lathrobium.elongatum
Stenus.bimaculatus
Lathrobium.fulvipenne
Tachinus.marginellus
Pterostichus.madidus
Stenus.juno
Leistus.rufomarginatus
Paranchus.albipes
Lathrobium.brunnipes
Philonthus.tenuicornis
Anotylus.sculpturatus
Geotrupes.stercorarius
Calathus.melanocephalus
Xantholinus.linearis
Ocypus.olens
Quedius.molochinus
Leistus.fulvibarbis
Bembidion.aeneum
Harpalus.rufipes
Athous.haemorrhoidalis
Tasgius.morsitans
Nebria.brevicollis
Anchomenus.dorsalis
Patrobus.atrorufus
Harpalus.affinis
Tachinus.rufipes
Stenus.clavicornis
Nicrophorus.investigator
Pterostichus.melanarius
Quedius.nitipennis
Calathus.fuscipes
Abax.parallelepipedus
Tachinus.humeralis
Loricera.pilicornis
Aleocharinae
Anotylus.rugosus
-2
0
2
Effect of Age on species occupancy
Mean species response +/- 95% CRIs

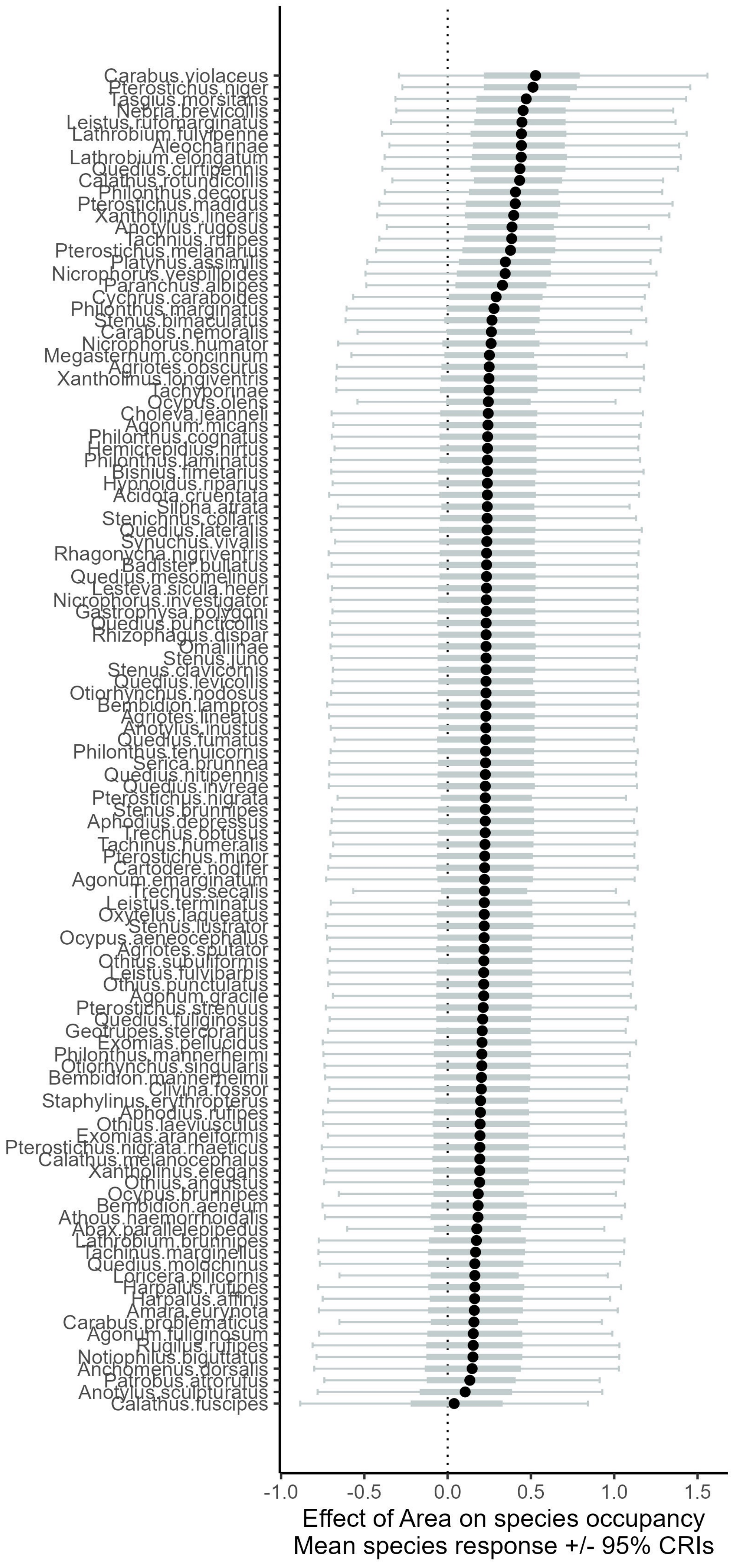
Carabus.violaceus
Pterostichus.niger
Tasgius.morsitans
Nebria.brevicollis
Leistus.rufomarginatus
Lathrobium.fulvipenne
Aleocharinae
Lathrobium.elongatum
Quedius.curtipennis
Calathus.rotundicollis
Philonthus.decorus
Pterostichus.madidus
Xantholinus.linearis
Anotylus.rugosus
Tachnius.rufipes
Pterostichus.melanarius
Platynus.assimilis
Nicrophorus.vespilloides
Paranchus.albipes
Cychrus.caraboides
Philonthus.marginatus
Stenus.bimaculatus
Carabus.nemoralis
Nicrophorus.humator
Megasternum.concinnum
Agriotes.obscurus
Xantholinus.longiventris
Tachyporinae
Ocypus.olens
Choleva.jeanneli
Agonum.micans
Philonthus.cognatus
Hemicrepidius.hirtus
Philonthus.laminatus
Bisnius.fimetarius
Hypnoidus.riparius
Acidota.cruentata
Silpha.atrata
Stenichnus.collaris
Quedius.lateralis
Synuchus.vivalis
Rhagonycha.nigriventris
Badister.bullatus
Quedius.mesomelinus
Lesteva.sicula.heeri
Nicrophorus.investigator
Gastrophysa.polygoni
Quedius.puncticollis
Rhizophagus.dispar
Omaliinae
Stenus.juno
Stenus.clavicornis
Quedius.levicollis
Otiorhynchus.nodosus
Bembidion.lampros
Agriotes.lineatus
Anotylus.inustus
Quedius.fumatus
Philonthus.tenuicornis
Serica.brunnea
Quedius.nitipennis
Quedius.invreae
Pterostichus.nigrata
Stenus.brunnipes
Aphodius.depressus
Trechus.obtusus
Tachinus.humeralis
Pterostichus.minor
Cartodere.nodifer
Agonum.emarginatum
Trechus.secalis
Leistus.terminatus
Oxytelus.laqueatus
Stenus.lustrator
Ocypus.aeneocephalus
Agriotes.sputator
Othius.subuliformis
Leistus.fulvibarbis
Othius.punctulatus
Agonum.gracile
Pterostichus.strenuus
Quedius.fuliginosus
Geotrupes.stercorarius
Exomias.pellucidus
Philonthus.mannerheimi
Otiorhynchus.singularis
Bembidion.mannerheimii
Clivina.fossor
Staphylinus.erythropterus
Aphodius.rufipes
Othius.laeviusculus
Exomias.araneiformis
Pterostichus.nigrata.rhaeticus
Calathus.melanocephalus
Xantholinus.elegans
Othius.angustus
Ocypus.brunnipes
Bembidion.aeneum
Athous.haemorrhoidalis
Abax.parallelepipedus
Lathrobium.brunnipes
Tachinus.marginellus
Quedius.molochinus
Loricera.pilicornis
Harpalus.rufipes
Harpalus.affinis
Amara.eurynota
Carabus.problematicus
Agonum.fuliginosum
Rugilus.rufipes
Notiophilus.biguttatus
Anchomenus.dorsalis
Patrobus.atrorufus
Anotylus.sculpturatus
Calathus.fuscipes
-1.0
-0.5
0.0
0.5
1.0
1.5
Effect of Area on species occupancy
Mean species response +/- 95% CRIs

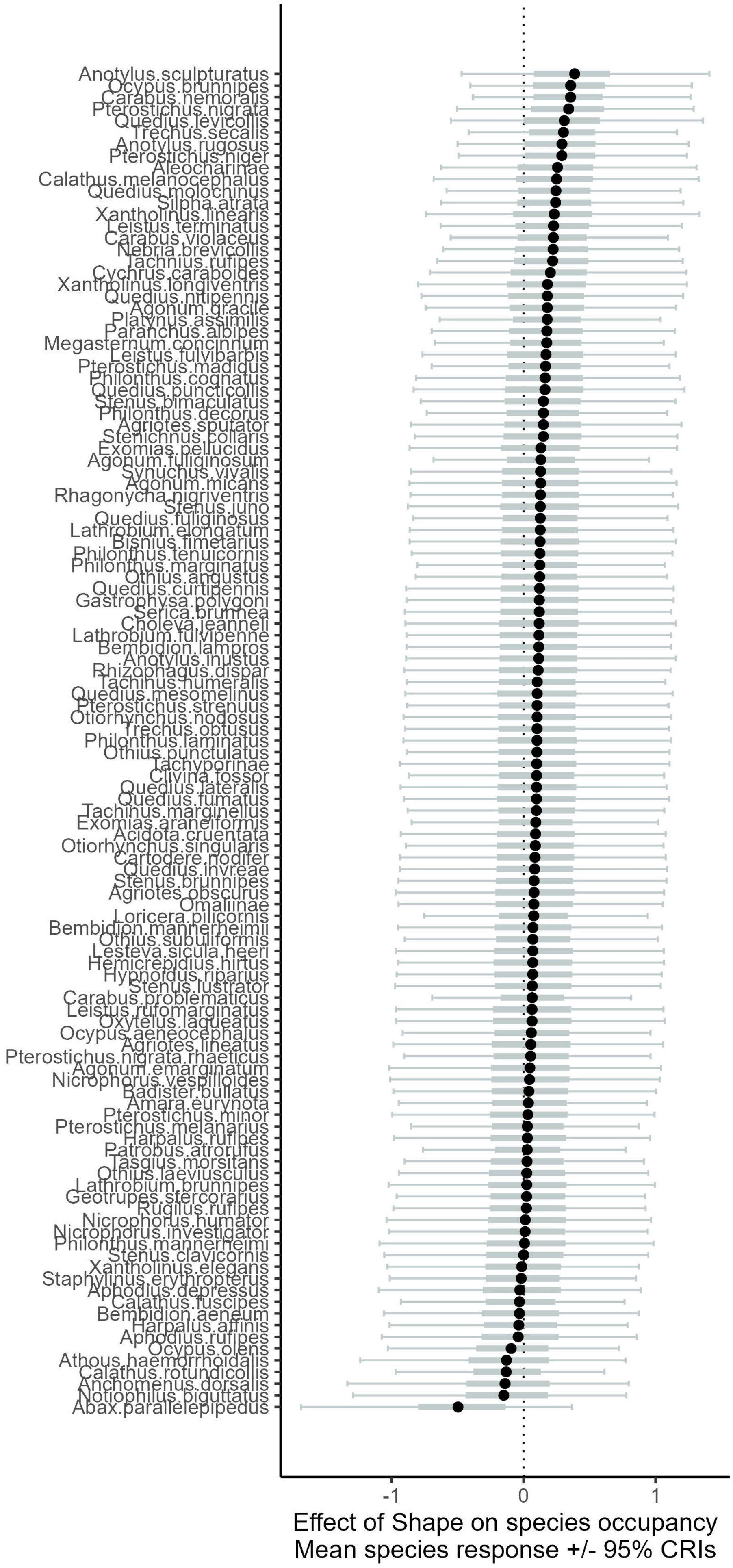
Anotylus.sculpturatus
Ocypus.brunnipes
Carabus.nemoralis
Pterostichus.nigrata
Quedius.levicollis
Trechus.secalis
Anotylus.rugosus
Pterostichus.niger
Aleocharinae
Calathus.melanocephalus
Quedius.molochinus
Silpha.atrata
Xantholinus.linearis
Leistus.terminatus
Carabus.violaceus
Nebria.brevicollis
Tachnius.rufipes
Cychrus.caraboides
Xantholinus.longiventris
Quedius.nitipennis
Agonum.gracile
Platynus.assimilis
Paranchus.albipes
Megasternum.concinnum
Leistus.fulvibarbis
Pterostichus.madidus
Philonthus.cognatus
Quedius.puncticollis
Stenus.bimaculatus
Philonthus.decorus
Agriotes.sputator
Stenichnus.collaris
Exomias.pellucidus
Agonum.fuliginosum
Synuchus.vivalis
Agonum.micans
Rhagonycha.nigriventris
Stenus.juno
Quedius.fuliginosus
Lathrobium.elongatum
Bisnius.fimetarius
Philonthus.tenuicornis
Philonthus.marginatus
Othius.angustus
Quedius.curtipennis
Gastrophysa.polygoni
Serica.brunnea
Choleva.jeanneli
Lathrobium.fulvipenne
Bembidion.lampros
Anotylus.inustus
Rhizophagus.dispar
Tachinus.humeralis
Quedius.mesomelinus
Pterostichus.strenuus
Otiorhynchus.nodosus
Trechus.obtusus
Philonthus.laminatus
Othius.punctulatus
Tachyporinae
Clivina.fossor
Quedius.lateralis
Quedius.fumatus
Tachinus.marginellus
Exomias.araneiformis
Acidota.cruentata
Otiorhynchus.singularis
Cartodere.nodifer
Quedius.invreae
Stenus.brunnipes
Agriotes.obscurus
Omaliinae
Loricera.pilicornis
Bembidion.mannerheimii
Othius.subuliformis
Lesteva.sicula.heeri
Hemicrepidius.hirtus
Hypnoidus.riparius
Stenus.lustrator
Carabus.problematicus
Leistus.rufomarginatus
Oxytelus.laqueatus
Ocypus.aeneocephalus
Agriotes.lineatus
Pterostichus.nigrata.rhaeticus
Agonum.emarginatum
Nicrophorus.vespilloides
Badister.bullatus
Amara.eurynota
Pterostichus.minor
Pterostichus.melanarius
Harpalus.rufipes
Patrobus.atrorufus
Tasgius.morsitans
Othius.laeviusculus
Lathrobium.brunnipes
Geotrupes.stercorarius
Rugilus.rufipes
Nicrophorus.humator
Nicrophorus.investigator
Philonthus.mannerheimi
Stenus.clavicornis
Xantholinus.elegans
Staphylinus.erythropterus
Aphodius.depressus
Calathus.fuscipes
Bembidion.aeneum
Harpalus.affinis
Aphodius.rufipes
Ocypus.olens
Athous.haemorrhoidalis
Calathus.rotundicollis
Anchomenus.dorsalis
Notiophilus.biguttatus
Abax.parallelepipedus
-1
0
1
Effect of Shape on species occupancy
Mean species response +/- 95% CRIs

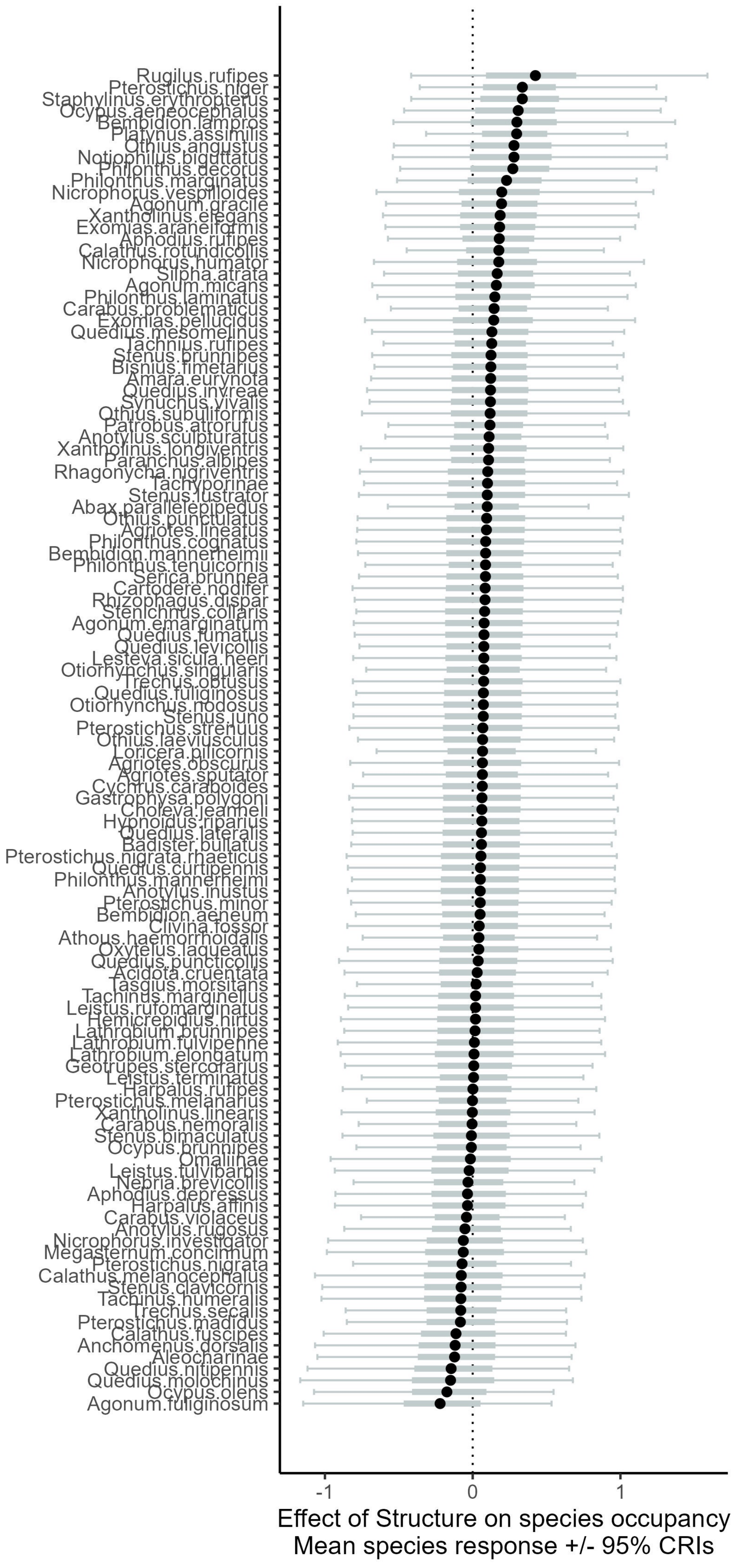

Rugilus.rufipes
Pterostichus.niger
Staphylinus.erythropterus
Ocypus.aeneocephalus
Bembidion.lampros
Platynus.assimilis
Othius.angustus
Notiophilus.biguttatus
Philonthus.decorus
Philonthus.marginatus
Nicrophorus.vespilloides
Agonum.gracile
Xantholinus.elegans
Exomias.araneiformis
Aphodius.rufipes
Calathus.rotundicollis
Nicrophorus.humator
Silpha.atrata
Agonum.micans
Philonthus.laminatus
Carabus.problematicus
Exomias.pellucidus
Quedius.mesomelinus
Tachnius.rufipes
Stenus.brunnipes
Bisnius.fimetarius
Amara.eurynota
Quedius.invreae
Synuchus.vivalis
Othius.subuliformis
Patrobus.atrorufus
Anotylus.sculpturatus
Xantholinus.longiventris
Paranchus.albipes
Rhagonycha.nigriventris
Tachyporinae
Stenus.lustrator
Abax.parallelepipedus
Othius.punctulatus
Agriotes.lineatus
Philonthus.cognatus
Bembidion.mannerheimii
Philonthus.tenuicornis
Serica.brunnea
Cartodere.nodifer
Rhizophagus.dispar
Stenichnus.collaris
Agonum.emarginatum
Quedius.fumatus
Quedius.levicollis
Lesteva.sicula.heeri
Otiorhynchus.singularis
Trechus.obtusus
Quedius.fuliginosus
Otiorhynchus.nodosus
Stenus.juno
Pterostichus.strenuus
Othius.laeviusculus
Loricera.pilicornis
Agriotes.obscurus
Agriotes.sputator
Cychrus.caraboides
Gastrophysa.polygoni
Choleva.jeanneli
Hypnoidus.riparius
Quedius.lateralis
Badister.bullatus
Pterostichus.nigrata.rhaeticus
Quedius.curtipennis
Philonthus.mannerheimi
Anotylus.inustus
Pterostichus.minor
Bembidion.aeneum
Clivina.fossor
Athous.haemorrhoidalis
Oxytelus.laqueatus
Quedius.puncticollis
Acidota.cruentata
Tasgius.morsitans
Tachinus.marginellus
Leistus.rufomarginatus
Hemicrepidius.hirtus
Lathrobium.brunnipes
Lathrobium.fulvipenne
Lathrobium.elongatum
Geotrupes.stercorarius
Leistus.terminatus
Harpalus.rufipes
Pterostichus.melanarius
Xantholinus.linearis
Carabus.nemoralis
Stenus.bimaculatus
Ocypus.brunnipes
Omaliinae
Leistus.fulvibarbis
Nebria.brevicollis
Aphodius.depressus
Harpalus.affinis
Carabus.violaceus
Anotylus.rugosus
Nicrophorus.investigator
Megasternum.concinnum
Pterostichus.nigrata
Calathus.melanocephalus
Stenus.clavicornis
Tachinus.humeralis
Trechus.secalis
Pterostichus.madidus
Calathus.fuscipes
Anchomenus.dorsalis
Aleocharinae
Quedius.nitipennis
Quedius.molochinus
Ocypus.olens
Agonum.fuliginosum
-1
0
1
Effect of Structure on species occupancy
Mean species response +/- 95% CRIs

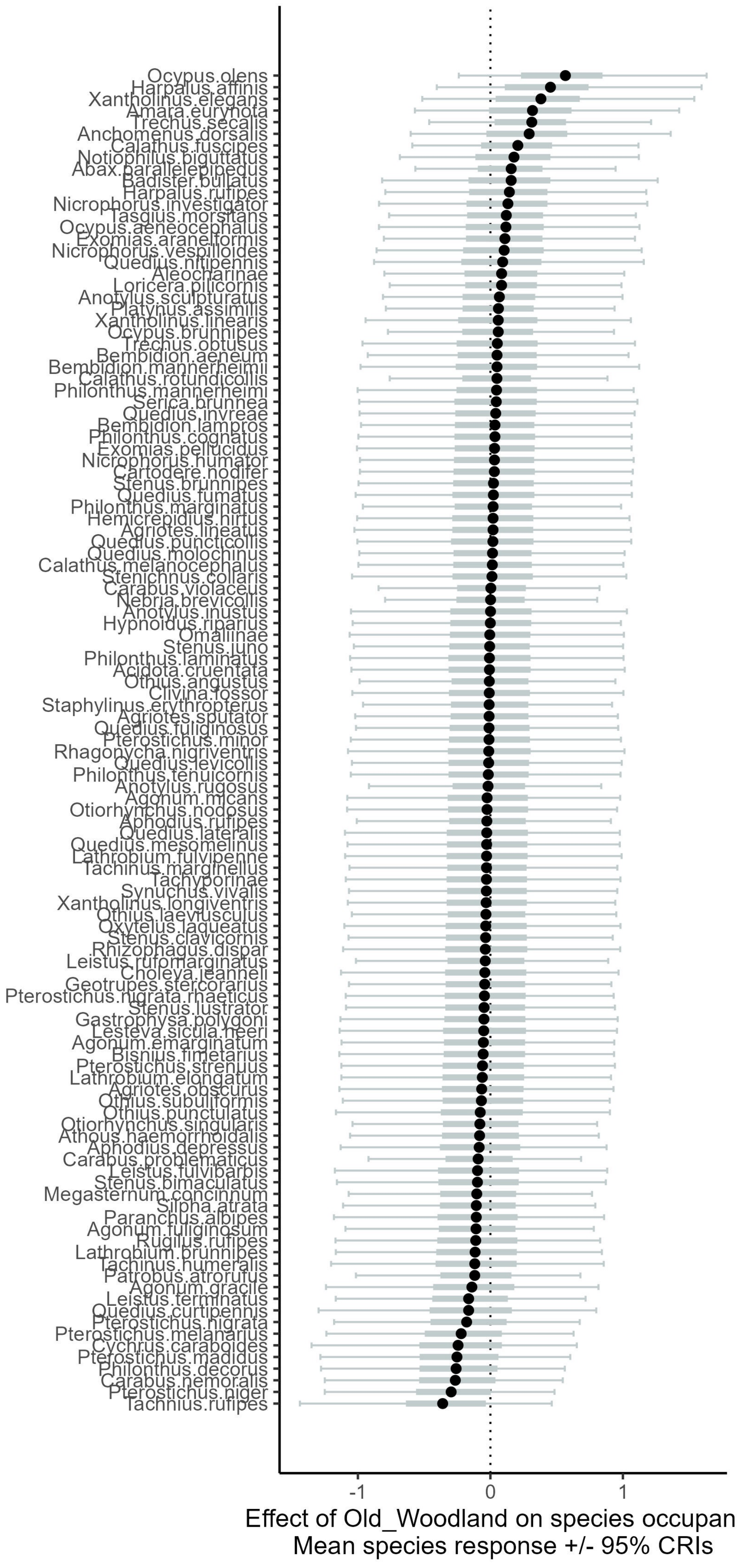

Ocypus.olens
Harpalus.affinis
Xantholinus.elegans
Amara.eurynota
Trechus.secalis
Anchomenus.dorsalis
Calathus.fuscipes
Notiophilus.biguttatus
Abax.parallelepipedus
Badister.bullatus
Harpalus.rufipes
Nicrophorus.investigator
Tasgius.morsitans
Ocypus.aeneocephalus
Exomias.araneiformis
Nicrophorus.vespilloides
Quedius.nitipennis
Aleocharinae
Loricera.pilicornis
Anotylus.sculpturatus
Platynus.assimilis
Xantholinus.linearis
Ocypus.brunnipes
Trechus.obtusus
Bembidion.aeneum
Bembidion.mannerheimii
Calathus.rotundicollis
Philonthus.mannerheimi
Serica.brunnea
Quedius.invreae
Bembidion.lampros
Philonthus.cognatus
Exomias.pellucidus
Nicrophorus.humator
Cartodere.nodifer
Stenus.brunnipes
Quedius.fumatus
Philonthus.marginatus
Hemicrepidius.hirtus
Agriotes.lineatus
Quedius.puncticollis
Quedius.molochinus
Calathus.melanocephalus
Stenichnus.collaris
Carabus.violaceus
Nebria.brevicollis
Anotylus.inustus
Hypnoidus.riparius
Omaliinae
Stenus.juno
Philonthus.laminatus
Acidota.cruentata
Othius.angustus
Clivina.fossor
Staphylinus.erythropterus
Agriotes.sputator
Quedius.fuliginosus
Pterostichus.minor
Rhagonycha.nigriventris
Quedius.levicollis
Philonthus.tenuicornis
Anotylus.rugosus
Agonum.micans
Otiorhynchus.nodosus
Aphodius.rufipes
Quedius.lateralis
Quedius.mesomelinus
Lathrobium.fulvipenne
Tachinus.marginellus
Tachyporinae
Synuchus.vivalis
Xantholinus.longiventris
Othius.laeviusculus
Oxytelus.laqueatus
Stenus.clavicornis
Rhizophagus.dispar
Leistus.rufomarginatus
Choleva.jeanneli
Geotrupes.stercorarius
Pterostichus.nigrata.rhaeticus
Stenus.lustrator
Gastrophysa.polygoni
Lesteva.sicula.heeri
Agonum.emarginatum
Bisnius.fimetarius
Pterostichus.strenuus
Lathrobium.elongatum
Agriotes.obscurus
Othius.subuliformis
Othius.punctulatus
Otiorhynchus.singularis
Athous.haemorrhoidalis
Aphodius.depressus
Carabus.problematicus
Leistus.fulvibarbis
Stenus.bimaculatus
Megasternum.concinnum
Silpha.atrata
Paranchus.albipes
Agonum.fuliginosum
Rugilus.rufipes
Lathrobium.brunnipes
Tachinus.humeralis
Patrobus.atrorufus
Agonum.gracile
Leistus.terminatus
Quedius.curtipennis
Pterostichus.nigrata
Pterostichus.melanarius
Cychrus.caraboides
Pterostichus.madidus
Philonthus.decorus
Carabus.nemoralis
Pterostichus.niger
Tachnius.rufipes
-1
0
1
Effect of Old_Woodland on species occupancy
Mean species response +/- 95% CRIs

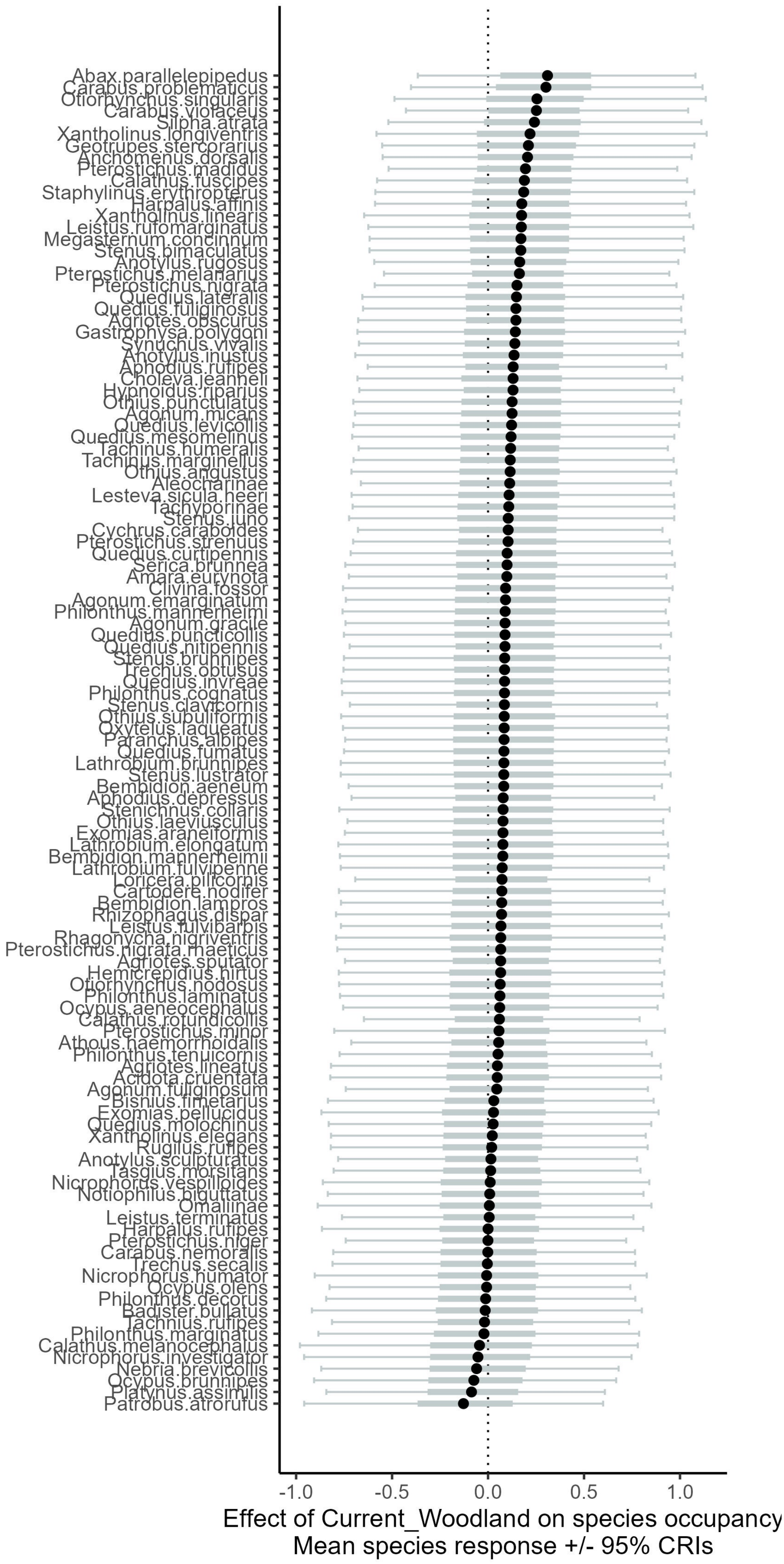
Abax.parallelepipedus
Carabus.problematicus
Otiorhynchus.singularis
Carabus.violaceus
Silpha.atrata
Xantholinus.longiventris
Geotrupes.stercorarius
Anchomenus.dorsalis
Pterostichus.madidus
Calathus.fuscipes
Staphylinus.erythropterus
Harpalus.affinis
Xantholinus.linearis
Leistus.rufomarginatus
Megasternum.concinnum
Stenus.bimaculatus
Anotylus.rugosus
Pterostichus.melanarius
Pterostichus.nigrata
Quedius.lateralis
Quedius.fuliginosus
Agriotes.obscurus
Gastrophysa.polygoni
Synuchus.vivalis
Anotylus.inustus
Aphodius.rufipes
Choleva.jeanneli
Hypnoidus.riparius
Othius.punctulatus
Agonum.micans
Quedius.levicollis
Quedius.mesomelinus
Tachinus.humeralis
Tachinus.marginellus
Othius.angustus
Aleocharinae
Lesteva.sicula.heeri
Tachyporinae
Stenus.juno
Cychrus.caraboides
Pterostichus.strenuus
Quedius.curtipennis
Serica.brunnea
Amara.eurynota
Clivina.fossor
Agonum.emarginatum
Philonthus.mannerheimi
Agonum.gracile
Quedius.puncticollis
Quedius.nitipennis
Stenus.brunnipes
Trechus.obtusus
Quedius.invreae
Philonthus.cognatus
Stenus.clavicornis
Othius.subuliformis
Oxytelus.laqueatus
Paranchus.albipes
Quedius.fumatus
Lathrobium.brunnipes
Stenus.lustrator
Bembidion.aeneum
Aphodius.depressus
Stenichnus.collaris
Othius.laeviusculus
Exomias.araneiformis
Lathrobium.elongatum
Bembidion.mannerheimii
Lathrobium.fulvipenne
Loricera.pilicornis
Cartodere.nodifer
Bembidion.lampros
Rhizophagus.dispar
Leistus.fulvibarbis
Rhagonycha.nigriventris
Pterostichus.nigrata.rhaeticus
Agriotes.sputator
Hemicrepidius.hirtus
Otiorhynchus.nodosus
Philonthus.laminatus
Ocypus.aeneocephalus
Calathus.rotundicollis
Pterostichus.minor
Athous.haemorrhoidalis
Philonthus.tenuicornis
Agriotes.lineatus
Acidota.cruentata
Agonum.fuliginosum
Bisnius.fimetarius
Exomias.pellucidus
Quedius.molochinus
Xantholinus.elegans
Rugilus.rufipes
Anotylus.sculpturatus
Tasgius.morsitans
Nicrophorus.vespilloides
Notiophilus.biguttatus
Omaliinae
Leistus.terminatus
Harpalus.rufipes
Pterostichus.niger
Carabus.nemoralis
Trechus.secalis
Nicrophorus.humator
Ocypus.olens
Philonthus.decorus
Badister.bullatus
Tachnius.rufipes
Philonthus.marginatus
Calathus.melanocephalus
Nicrophorus.investigator
Nebria.brevicollis
Ocypus.brunnipes
Platynus.assimilis
Patrobus.atrorufus
-1.0
-0.5
0.0
0.5
1.0
Effect of Current_Woodland on species occupancy
Mean species response +/- 95% CRIs

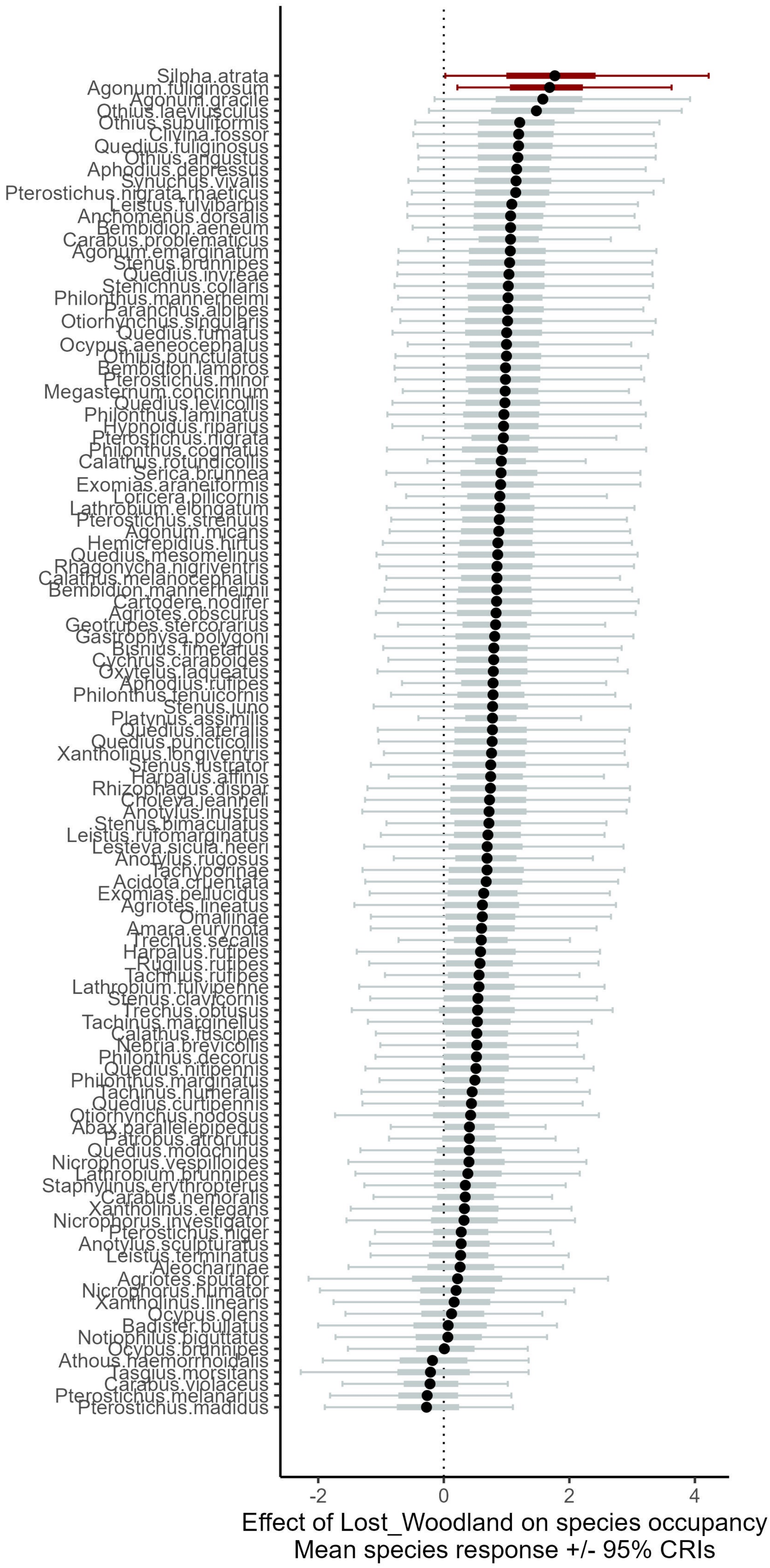

Silpha.atrata
Agonum.fuliginosum
Agonum.gracile
Othius.laeviusculus
Othius.subuliformis
Clivina.fossor
Quedius.fuliginosus
Othius.angustus
Aphodius.depressus
Synuchus.vivalis
Pterostichus.nigrata.rhaeticus
Leistus.fulvibarbis
Anchomenus.dorsalis
Bembidion.aeneum
Carabus.problematicus
Agonum.emarginatum
Stenus.brunnipes
Quedius.invreae
Stenichnus.collaris
Philonthus.mannerheimi
Paranchus.albipes
Otiorhynchus.singularis
Quedius.fumatus
Ocypus.aeneocephalus
Othius.punctulatus
Bembidion.lampros
Pterostichus.minor
Megasternum.concinnum
Quedius.levicollis
Philonthus.laminatus
Hypnoidus.riparius
Pterostichus.nigrata
Philonthus.cognatus
Calathus.rotundicollis
Serica.brunnea
Exomias.araneiformis
Loricera.pilicornis
Lathrobium.elongatum
Pterostichus.strenuus
Agonum.micans
Hemicrepidius.hirtus
Quedius.mesomelinus
Rhagonycha.nigriventris
Calathus.melanocephalus
Bembidion.mannerheimii
Cartodere.nodifer
Agriotes.obscurus
Geotrupes.stercorarius
Gastrophysa.polygoni
Bisnius.fimetarius
Cychrus.caraboides
Oxytelus.laqueatus
Aphodius.rufipes
Philonthus.tenuicornis
Stenus.juno
Platynus.assimilis
Quedius.lateralis
Quedius.puncticollis
Xantholinus.longiventris
Stenus.fustrator
Harpalus.affinis
Rhizophagus.dispar
Choleva.jeanneli
Anotylus.inustus
Stenus.bimaculatus
Leistus.rufomarginatus
Lesteva.sicula.heeri
Anotylus.rugosus
Tachyporinae
Acidota.cruentata
Exomias.pellucidus
Agriotes.lineatus
Omaliinae
Amara.eurynota
Trechus.secalis
Harpalus.rufipes
Rugilus.rufipes
Tachinus.rufipes
Lathrobium.fulvipenne
Stenus.clavicornis
Trechus.obtusus
Tachinus.marginellus
Calathus.fuscipes
Nebria.brevicollis
Philonthus.decorus
Quedius.nitipennis
Philonthus.marginatus
Tachinus.humeralis
Quedius.curtipennis
Otiorhynchus.nodosus
Abax.parallelepipedus
Patrobus.atrorufus
Quedius.molochinus
Nicrophorus.vespilloides
Lathrobium.brunnipes
Staphylinus.erythropterus
Carabus.nemoralis
Xantholinus.elegans
Nicrophorus.investigator
Pterostichus.niger
Anotylus.sculpturatus
Leistus.terminatus
Aleocharinae
Agriotes.sputator
Nicrophorus.humator
Xantholinus.linearis
Ocypus.olens
Badister.bullatus
Notiophilus.biguttatus
Ocypus.brunnipes
Athous.haemorrhoidalis
Tasgius.morsitans
Carabus.violaceus
Pterostichus.melanarius
Pterostichus.madidus
-2
0
2
4
Effect of Lost_Woodland on species occupancy
Mean species response +/- 95% CRIs

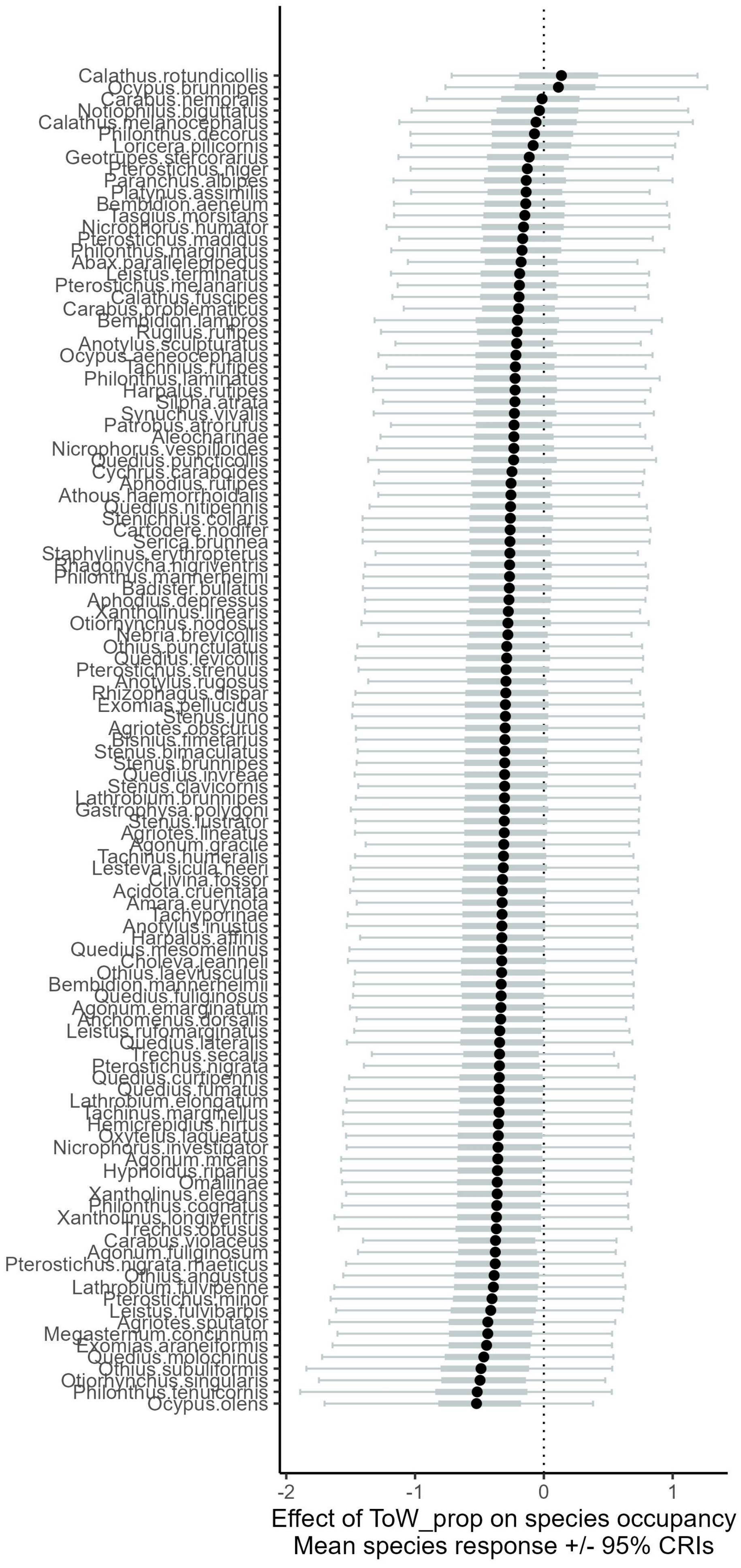

Calathus.rotundicollis
Ocypus.brunnipes
Carabus.nemoralis
Notiophilus.biguttatus
Calathus.melanocephalus
Philonthus.decorus
Loricera.pilicornis
Geotrupes.stercorarius
Pterostichus.niger
Paranchus.albipes
Platynus.assimilis
Bembidion.aeneum
Tasgius.morsitans
Nicrophorus.humator
Pterostichus.madidus
Philonthus.marginatus
Abax.parallelepipedus
Leistus.terminatus
Pterostichus.melanarius
Calathus.fuscipes
Carabus.problematicus
Bembidion.lampros
Rugilus.rufipes
Anotylus.sculpturatus
Ocypus.aeneocephalus
Tachnius.rufipes
Philonthus.laminatus
Harpalus.rufipes
Silpha.atrata
Synuchus.vivalis
Patrobus.atrorufus
Aleocharinae
Nicrophorus.vespilloides
Quedius.puncticollis
Cychrus.caraboides
Aphodius.rufipes
Athous.haemorrhoidalis
Quedius.nitipennis
Stenichnus.collaris
Cartodere.nodifer
Serica.brunnea
Staphylinus.erythropterus
Rhagonycha.nigriventris
Philonthus.mannerheimi
Badister.bullatus
Aphodius.depressus
Xantholinus.linearis
Otiorhynchus.nodosus
Nebria.brevicollis
Othius.punctulatus
Quedius.levicollis
Pterostichus.strenuus
Anotylus.rugosus
Rhizophagus.dispar
Exomias.pellucidus
Stenus.juno
Agriotes.obscurus
Bisnius.fimetarius
Stenus.bimaculatus
Stenus.brunnipes
Quedius.invreae
Stenus.clavicornis
Lathrobium.brunnipes
Gastrophysa.polygoni
Stenus.lustrator
Agriotes.lineatus
Agonum.gracile
Tachinus.humeralis
Lesteva.sicula.heeri
Clivina.fossor
Acidota.cruentata
Amara.eurynota
Tachyporinae
Anotylus.inustus
Harpalus.affinis
Quedius.mesomelinus
Choleva.jeanneli
Othius.laeviusculus
Bembidion.mannerheimii
Quedius.fuliginosus
Agonum.emarginatum
Anchomenus.dorsalis
Leistus.rufomarginatus
Quedius.lateralis
Trechus.secalis
Pterostichus.nigrata
Quedius.curtipennis
Quedius.fumatus
Lathrobium.elongatum
Tachinus.marginellus
Hemicrepidius.hirtus
Oxytelus.laqueatus
Nicrophorus.investigator
Agonum.micans
Hypnoidus.riparius
Omaliinae
Xantholinus.elegans
Philonthus.cognatus
Xantholinus.longiventris
Trechus.obtusus
Carabus.violaceus
Agonum.fuliginosum
Pterostichus.nigrata.maeticus
Othius.angustus
Lathrobium.fulvipenne
Pterostichus.minor
Leistus.fulvibarbis
Agriotes.sputator
Megasternum.concinnum
Exomias.araneiformis
Quedius.molochinus
Othius.subuliformis
Otiorhynchus.singularis
Philonthus.tenuicornis
Ocypus.olens
-2
-1
0
1
Effect of ToW_prop on species occupancy
Mean species response +/- 95% CRIs

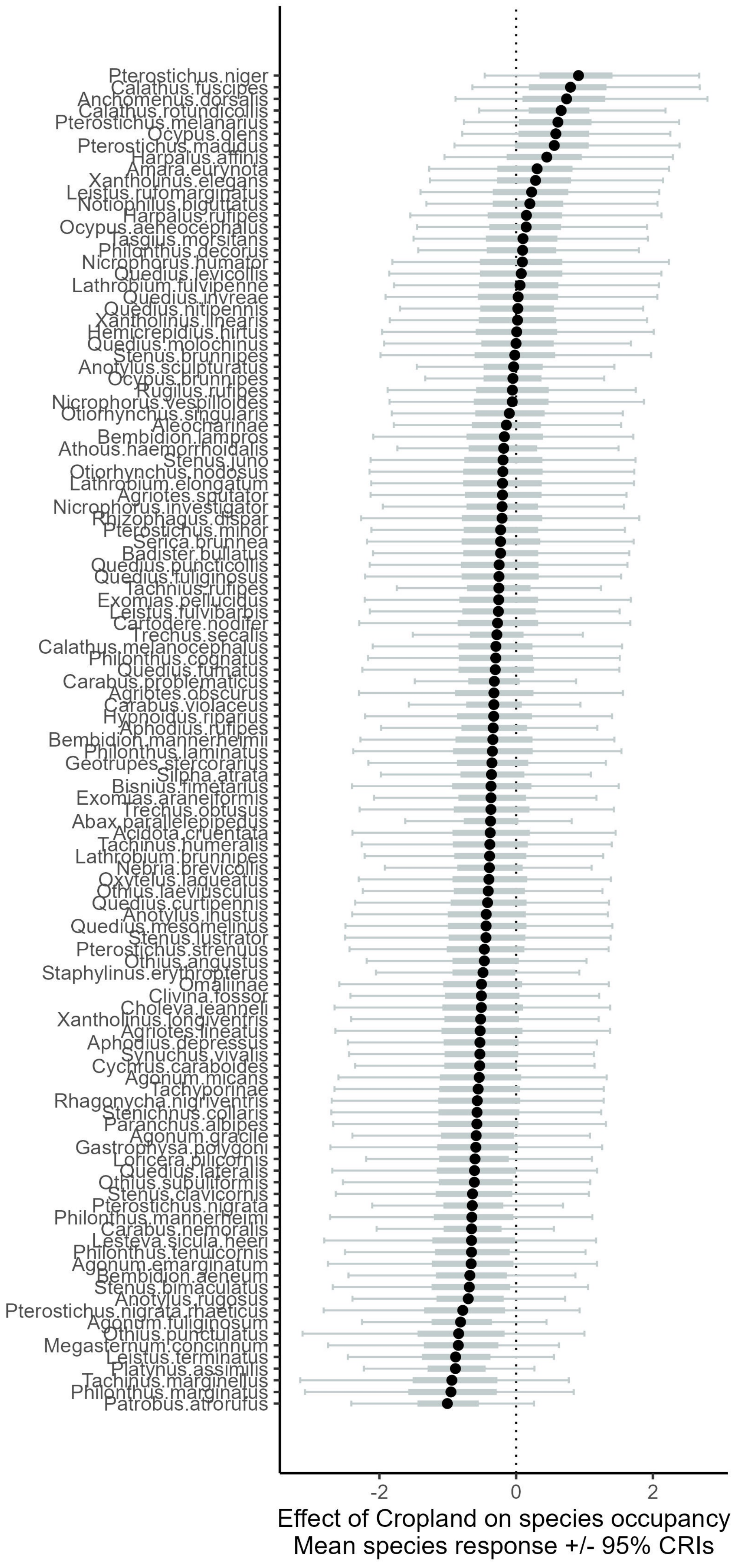
Pterostichus.niger
Calathus.fuscipes
Anchomenus.dorsalis
Calathus.rotundicollis
Pterostichus.melanarius
Ocypus.olens
Pterostichus.madidus
Harpalus.affinis
Amara.eurynota
Xantholinus.elegans
Leistus.rufomarginatus
Notiophilus.biguttatus
Harpalus.rufipes
Ocypus.aeneocephalus
Tasgius.morsitans
Philonthus.decorus
Nicrophorus.humator
Quedius.levicollis
Lathrobium.fulvipenne
Quedius.invreae
Quedius.nitipennis
Xantholinus.linearis
Hemicrepidius.hirtus
Quedius.molochinus
Stenus.brunnipes
Anotylus.sculpturatus
Ocypus.brunnipes
Rugilus.rufipes
Nicrophorus.vespilloides
Otiorhynchus.singularis
Aleocharinae
Bembidion.lampros
Athous.haemorrhoidalis
Stenus.juno
Otiorhynchus.nodosus
Lathrobium.elongatum
Agriotes.sputator
Nicrophorus.investigator
Rhizophagus.dispar
Pterostichus.minor
Serica.brunnea
Badister.bullatus
Quedius.puncticollis
Quedius.fuliginosus
Tachnius.rufipes
Exomias.pellucidus
Leistus.fulvibarbis
Cartodere.nodifer
Trechus.secalis
Calathus.melanocephalus
Philonthus.cognatus
Quedius.fumatus
Carabus.problematicus
Agriotes.obscurus
Carabus.violaceus
Hypnoidus.riparius
Aphodius.rufipes
Bembidion.mannerheimii
Philonthus.laminatus
Geotrupes.stercorarius
Silpha.atrata
Bisnius.fimetarius
Exomias.araneiformis
Trechus.obtusus
Abax.parallelepipedus
Acidota.cruentata
Tachinus.humeralis
Lathrobium.brunnipes
Nebria.brevicollis
Oxytelus.laqueatus
Othius.laeviusculus
Quedius.curtipennis
Anotylus.inustus
Quedius.mesomelinus
Stenus.lustrator
Pterostichus.strenuus
Othius.angustus
Staphylinus.erythropterus
Omaliinae
Clivina.fossor
Choleva.jeanneli
Xantholinus.longiventris
Agriotes.lineatus
Aphodius.depressus
Synuchus.vivalis
Cychrus.caraboides
Agonum.micans
Tachyporinae
Rhagonycha.nigriventris
Stenichnus.collaris
Paranchus.albipes
Agonum.gracile
Gastrophysa.polygoni
Loricera.pilicornis
Quedius.lateralis
Othius.subuliformis
Stenus.clavicornis
Pterostichus.nigrita
Philonthus.mannerheimi
Carabus.nemoralis
Lesteva.sicula.heeri
Philonthus.tenuicornis
Agonum.emarginatum
Bembidion.aeneum
Stenus.bimaculatus
Anotylus.rugosus
Pterostichus.nigrita.rhaeticus
Agonum.fuliginosum
Othius.punctulatus
Megasternum.concinnum
Leistus.terminatus
Platynus.assimilis
Tachinus.marginellus
Philonthus.marginatus
Patrobus.atrorufus
-2
0
2
Effect of Cropland on species occupancy
Mean species response +/- 95% CRIs

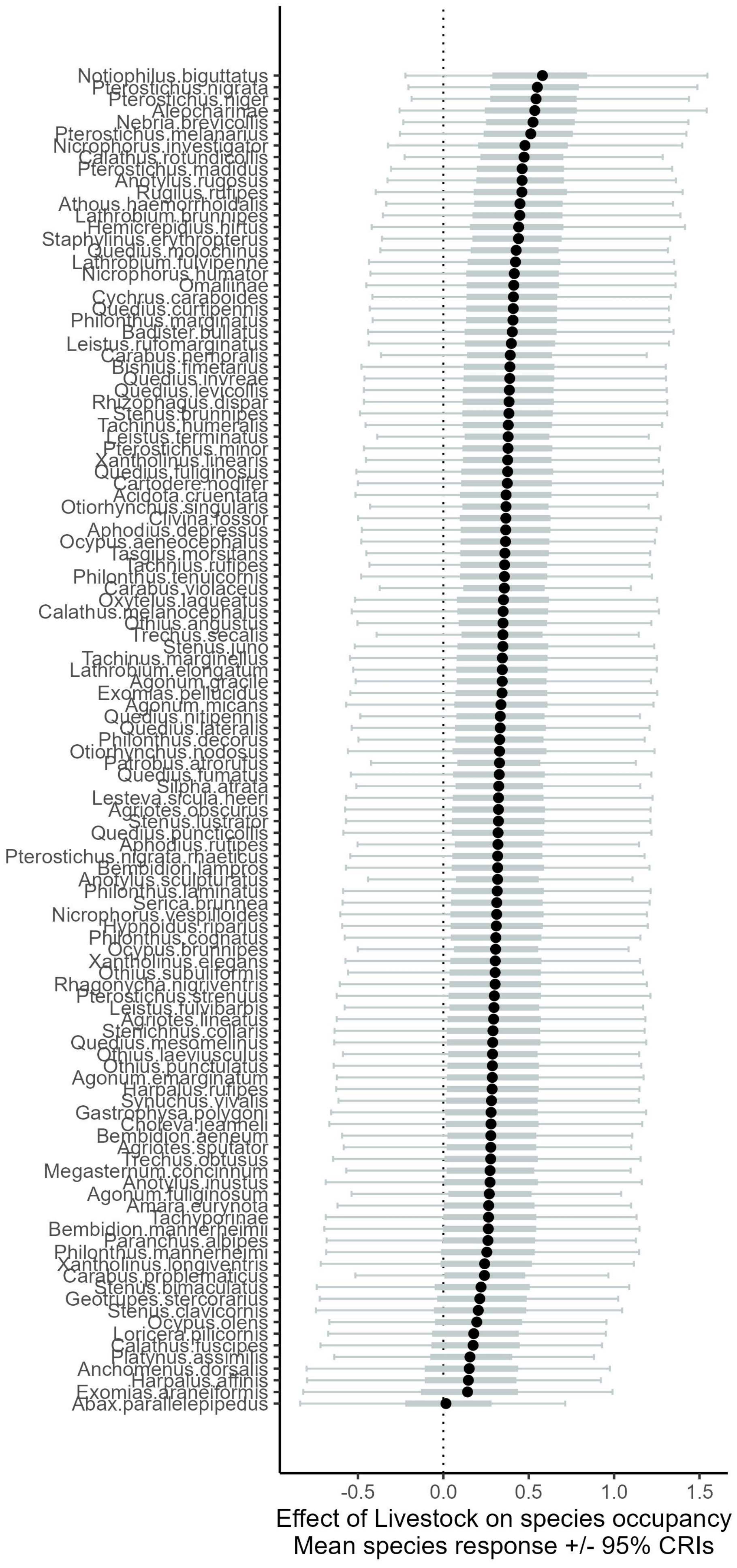
Notiophilus.biguttatus
Pterostichus.nigrata
Pterostichus.niger
Aleocharinae
Nebria.brevicollis
Pterostichus.melanarius
Nicrophorus.investigator
Calathus.rotundicollis
Pterostichus.madidus
Anotylus.rugosus
Rugilus.rufipes
Athous.haemorrhoidalis
Lathrobium.brunnipes
Hemicrepidius.hirtus
Staphylinus.erythropterus
Quedius.molochinus
Lathrobium.fulvipenne
Nicrophorus.humator
Omaliinae
Cychrus.caraboides
Quedius.curtipennis
Philonthus.marginatus
Badister.bullatus
Leistus.rufomarginatus
Carabus.nemoralis
Bisnius.fimetarius
Quedius.invreae
Quedius.levicollis
Rhizophagus.dispar
Stenus.brunnipes
Tachinus.humeralis
Leistus.terminatus
Pterostichus.minor
Xantholinus.linearis
Quedius.fuliginosus
Cartodere.nodifer
Acidota.cruentata
Otiorhynchus.singularis
Clivina.fossor
Aphodius.depressus
Ocypus.aeneocephalus
Tasgius.morsitans
Tachinus.rufipes
Philonthus.tenuicornis
Carabus.violaceus
Oxytelus.laqueatus
Calathus.melanocephalus
Othius.angustus
Trechus.secalis
Stenus.juno
Tachinus.marginellus
Lathrobium.elongatum
Agonum.gracile
Exomias.pellucidus
Agonum.micans
Quedius.nitipennis
Quedius.lateralis
Philonthus.decorus
Otiorhynchus.nodosus
Patrobus.atrorufus
Quedius.fumatus
Silpha.atrata
Lesteva.sicula.heeri
Agriotes.obscurus
Stenus.lustrator
Quedius.puncticollis
Aphodius.rufipes
Pterostichus.nigrata.rhaeticus
Bembidion.lampros
Anotylus.sculpturatus
Philonthus.laminatus
Serica.brunnea
Nicrophorus.vespilloides
Hypnoidus.riparius
Philonthus.cognatus
Ocypus.brunnipes
Xantholinus.elegans
Othius.subuliformis
Rhagonycha.nigriventris
Pterostichus.strenuus
Leistus.fulvibarbis
Agriotes.lineatus
Stenichnus.collaris
Quedius.mesomelinus
Othius.laeviusculus
Othius.punctulatus
Agonum.emarginatum
Harpalus.rufipes
Synuchus.vivalis
Gastrophysa.polygoni
Choleva.jeanneli
Bembidion.aeneum
Agriotes.sputator
Trechus.obtusus
Megasternum.concinnum
Anotylus.inustus
Agonum.fuliginosum
Amara.eurynota
Tachyporinae
Bembidion.mannerheimii
Paranchus.albipes
Philonthus.mannerheimi
Xantholinus.longiventris
Carabus.problematicus
Stenus.bimaculatus
Geotrupes.stercorarius
Stenus.clavicornis
Ocypus.olens
Loricera.pilicornis
Calathus.fuscipes
Platynus.assimilis
Anchomenus.dorsalis
Harpalus.affinis
Exomias.araneiformis
Abax.parallelepipedus
-0.5
0.0
0.5
1.0
1.5
Effect of Livestock on species occupancy
Mean species response +/- 95% CRIs

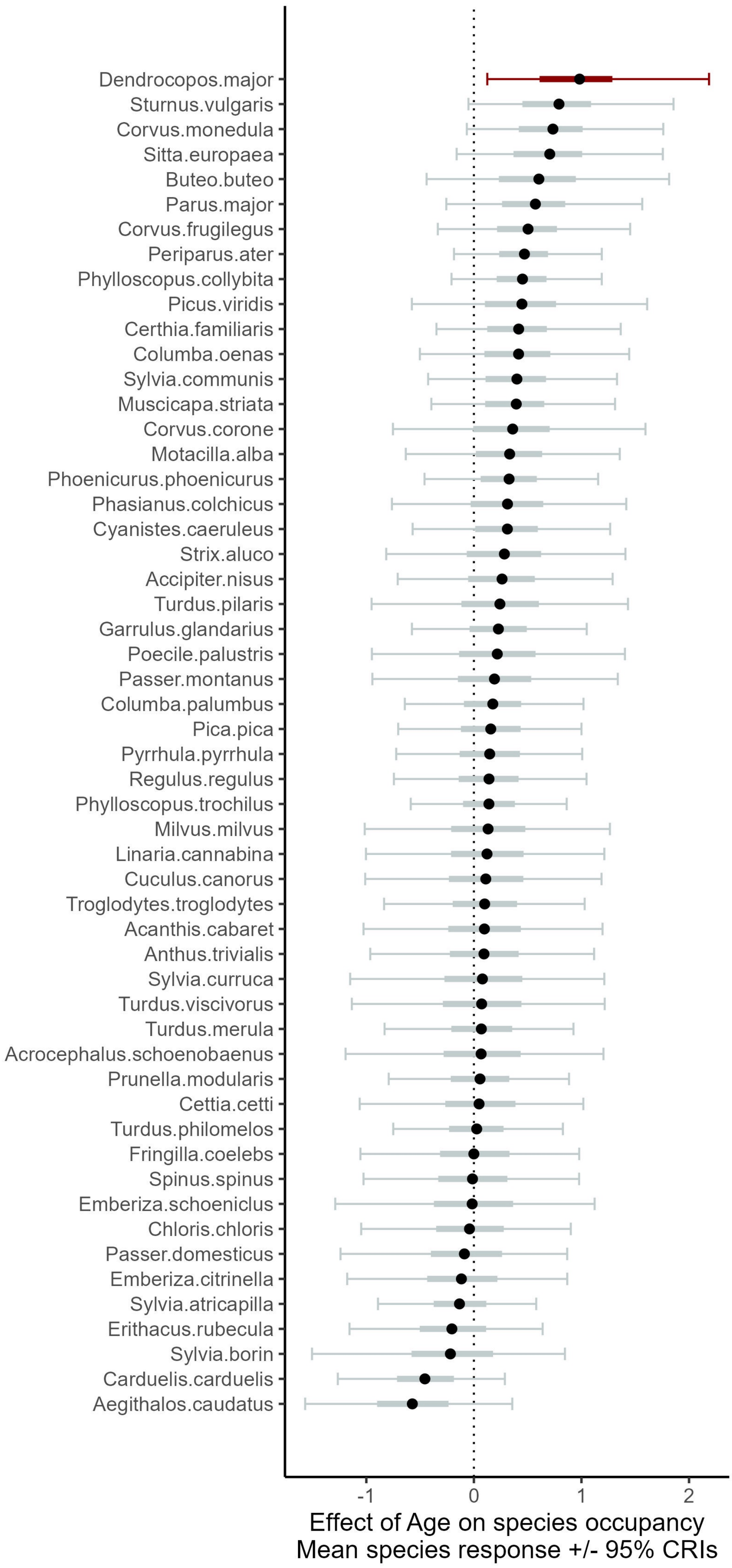

Dendrocopos.major
Sturnus.vulgaris
Corvus.monedula
Sitta.europaea
Buteo.buteo
Parus.major
Corvus.frugilegus
Periparus.ater
Phylloscopus.collybita
Picus.viridis
Certhia.familiaris
Columba.oenas
Sylvia.communis
Muscicapa.striata
Corvus.corone
Motacilla.alba
Phoenicurus.phoenicurus
Phasianus.colchicus
Cyanistes.caeruleus
Strix.aluco
Accipiter.nisus
Turdus.pilaris
Garrulus.glandarius
Poecile.palustris
Passer.montanus
Columba.palumbus
Pica.pica
Pyrrhula.pyrrhula
Regulus.regulus
Phylloscopus.trochilus
Milvus.milvus
Linaria.cannabina
Cuculus.canorus
Troglodytes.troglodytes
Acanthis.cabaret
Anthus.trivialis
Sylvia.curruca
Turdus.viscivorus
Turdus.merula
Acrocephalus.schoenobaenus
Prunella.modularis
Cettia.cetti
Turdus.philomelos
Fringilla.coelebs
Spinus.spinus
Emberiza.schoeniclus
Chloris.chloris
Passer.domesticus
Emberiza.citrinella
Sylvia.atricapilla
Erithacus.rubecula
Sylvia.borin
Carduelis.carduelis
Aegithalos.caudatus
-1
0
1
2
Effect of Age on species occupancy
Mean species response +/- 95% CRIs

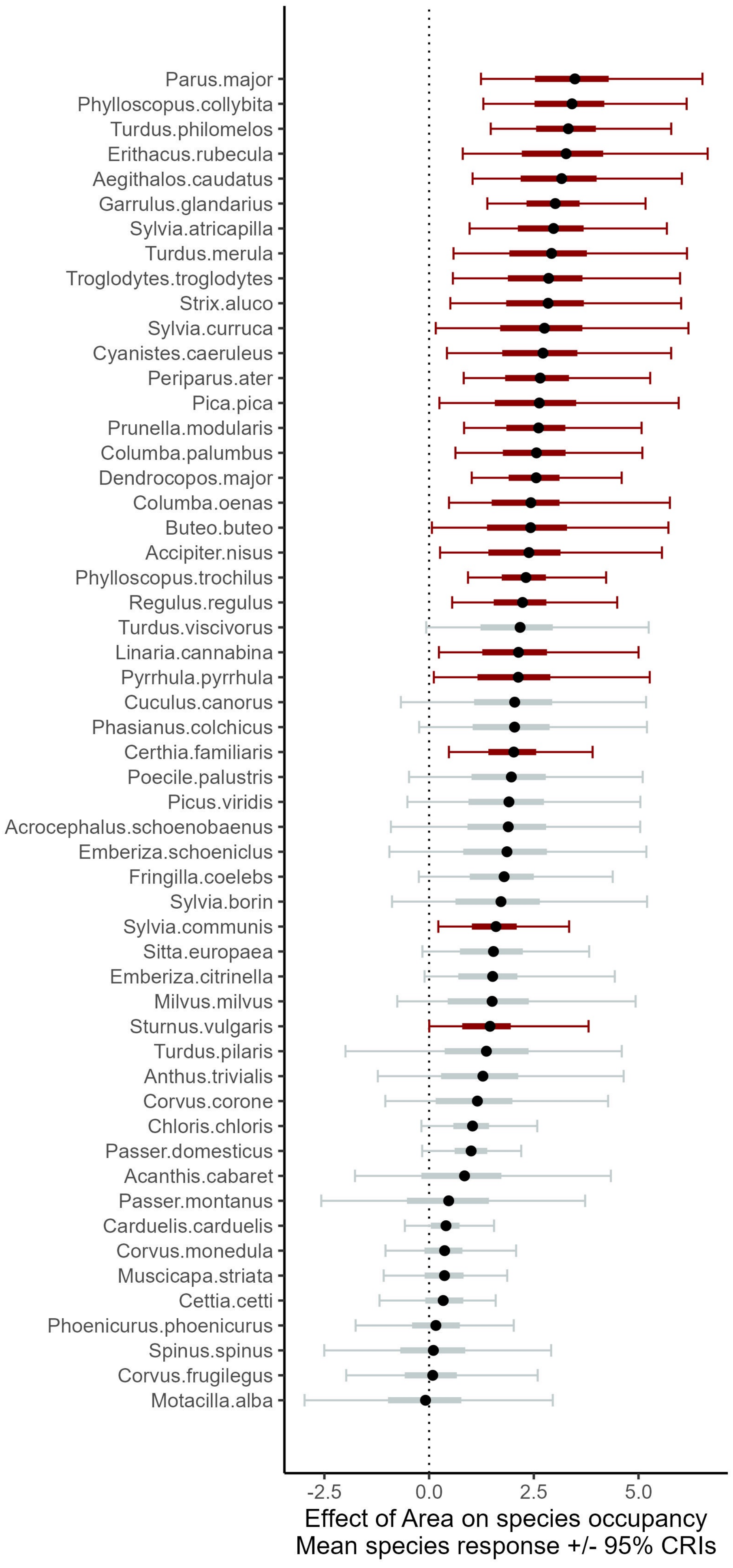
Parus.major
Phylloscopus.collybita
Turdus.philomelos
Erithacus.rubecula
Aegithalos.caudatus
Garrulus.glandarius
Sylvia.atricapilla
Turdus.merula
Troglodytes.troglodytes
Strix.aluco
Sylvia.curruca
Cyanistes.caeruleus
Periparus.ater
Pica.pica
Prunella.modularis
Columba.palumbus
Dendrocopos.major
Columba.oenas
Buteo.buteo
Accipiter.nisus
Phylloscopus.trochilus
Regulus.regulus
Turdus.viscivorus
Linaria.cannabina
Pyrrhula.pyrrhula
Cuculus.canorus
Phasianus.colchicus
Certhia.familiaris
Poecile.palustris
Picus.viridis
Acrocephalus.schoenobaenus
Emberiza.schoeniclus
Fringilla.coelebs
Sylvia.borin
Sylvia.communis
Sitta.europaea
Emberiza.citrinella
Milvus.milvus
Sturnus.vulgaris
Turdus.pilaris
Anthus.trivialis
Corvus.corone
Chloris.chloris
Passer.domesticus
Acanthis.cabaret
Passer.montanus
Carduelis.carduelis
Corvus.monedula
Muscicapa.striata
Cettia.cetti
Phoenicurus.phoenicurus
Spinus.spinus
Corvus.frugilegus
Motacilla.alba
-2.5
0.0
2.5
5.0
Effect of Area on species occupancy
Mean species response +/- 95% CRIs

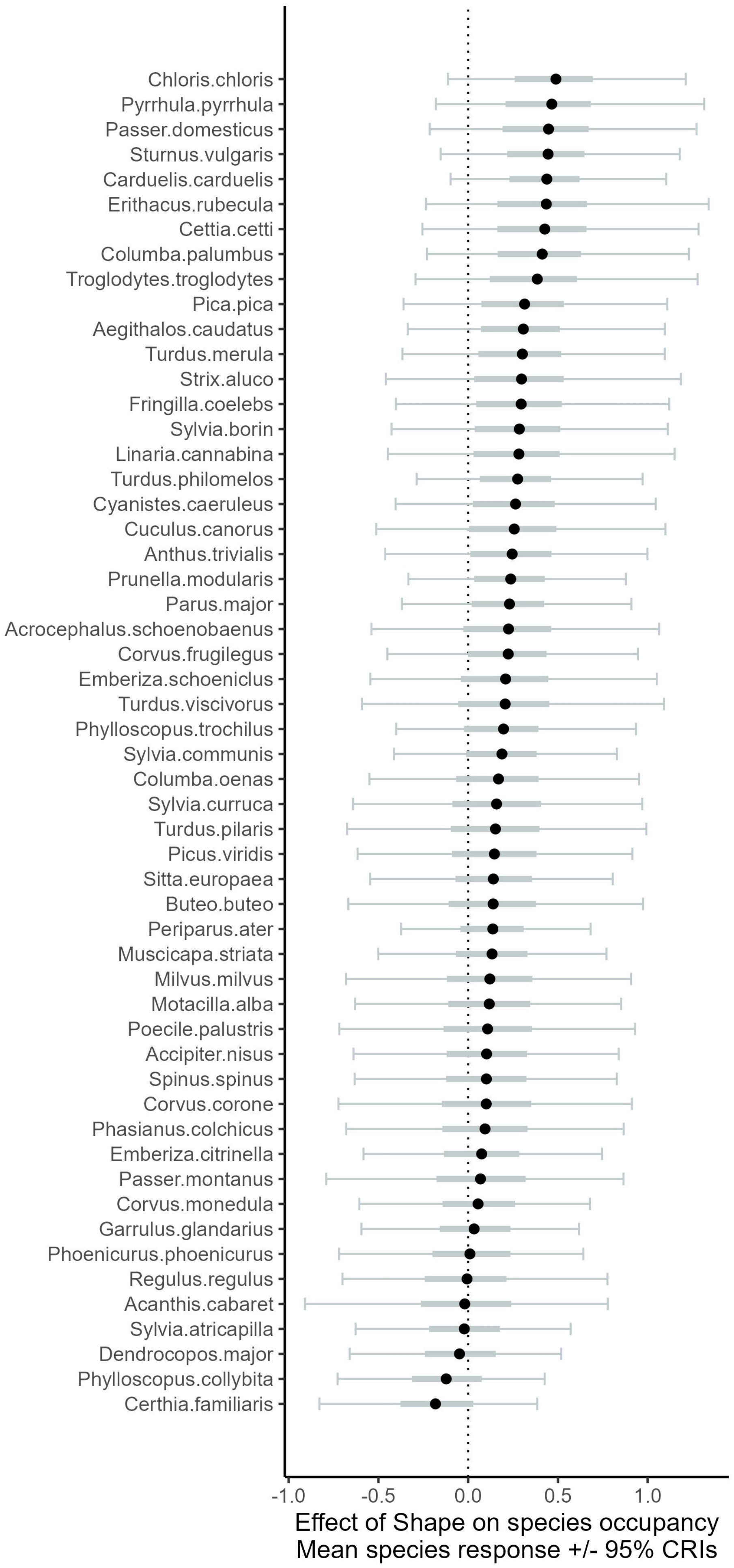
Chloris.chloris
Pyrrhula.pyrrhula
Passer.domesticus
Sturnus.vulgaris
Carduelis.carduelis
Erithacus.rubecula
Cettia.cetti
Columba.palumbus
Troglodytes.troglodytes
Pica.pica
Aegithalos.caudatus
Turdus.merula
Strix.aluco
Fringilla.coelebs
Sylvia.borin
Linaria.cannabina
Turdus.philomelos
Cyanistes.caeruleus
Cuculus.canorus
Anthus.trivialis
Prunella.modularis
Parus.major
Acrocephalus.schoenobaenus
Corvus.frugilegus
Emberiza.schoeniclus
Turdus.viscivorus
Phylloscopus.trochilus
Sylvia.communis
Columba.oenas
Sylvia.curruca
Turdus.pilaris
Picus.viridis
Sitta.europaea
Buteo.buteo
Periparus.ater
Muscicapa.striata
Milvus.milvus
Motacilla.alba
Poecile.palustris
Accipiter.nisus
Spinus.spinus
Corvus.corone
Phasianus.colchicus
Emberiza.citrinella
Passer.montanus
Corvus.monedula
Garrulus.glandarius
Phoenicurus.phoenicurus
Regulus.regulus
Acanthis.cabaret
Sylvia.atricapilla
Dendrocopos.major
Phylloscopus.collybita
Certhia.familiaris
-1.0
-0.5
0.0
0.5
1.0
Effect of Shape on species occupancy
Mean species response +/- 95% CRIs

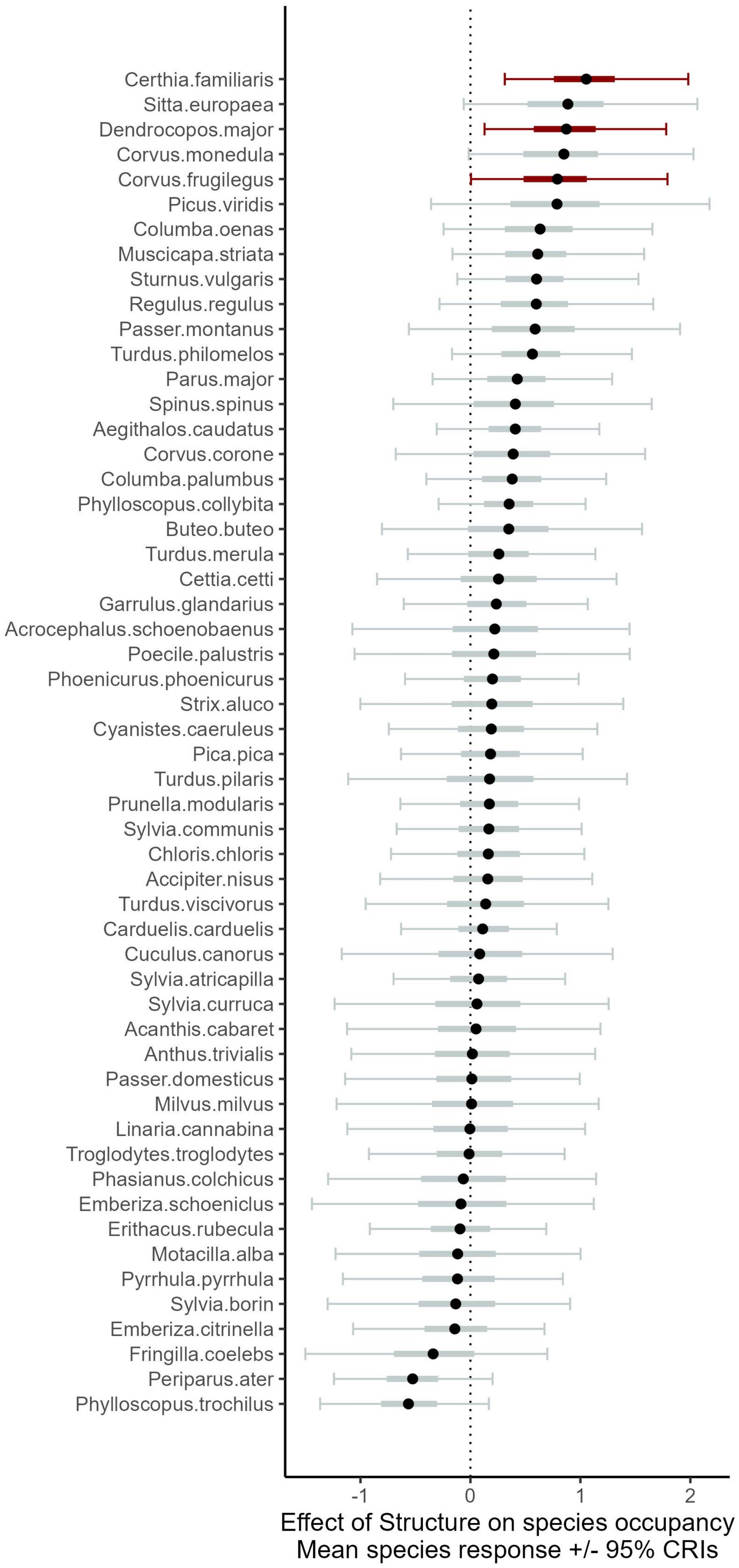
Certhia.familiaris
Sitta.europaea
Dendrocopos.major
Corvus.monedula
Corvus.frugilegus
Picus.viridis
Columba.oenas
Muscicapa.striata
Sturnus.vulgaris
Regulus.regulus
Passer.montanus
Turdus.philomelos
Parus.major
Spinus.spinus
Aegithalos.caudatus
Corvus.corone
Columba.palumbus
Phylloscopus.collybita
Buteo.buteo
Turdus.merula
Cettia.cetti
Garrulus.glandarius
Acrocephalus.schoenobaenus
Poecile.palustris
Phoenicurus.phoenicurus
Strix.aluco
Cyanistes.caeruleus
Pica.pica
Turdus.pilaris
Prunella.modularis
Sylvia.communis
Chloris.chloris
Accipiter.nisus
Turdus.viscivorus
Carduelis.carduelis
Cuculus.canorus
Sylvia.atricapilla
Sylvia.curruca
Acanthis.cabaret
Anthus.trivialis
Passer.domesticus
Milvus.milvus
Linaria.cannabina
Troglodytes.troglodytes
Phasianus.colchicus
Emberiza.schoeniclus
Erithacus.rubecula
Motacilla.alba
Pyrrhula.pyrrhula
Sylvia.borin
Emberiza.citrinella
Fringilla.coelebs
Periparus.ater
Phylloscopus.trochilus
-1
0
1
2
Effect of Structure on species occupancy
Mean species response +/- 95% CRIs

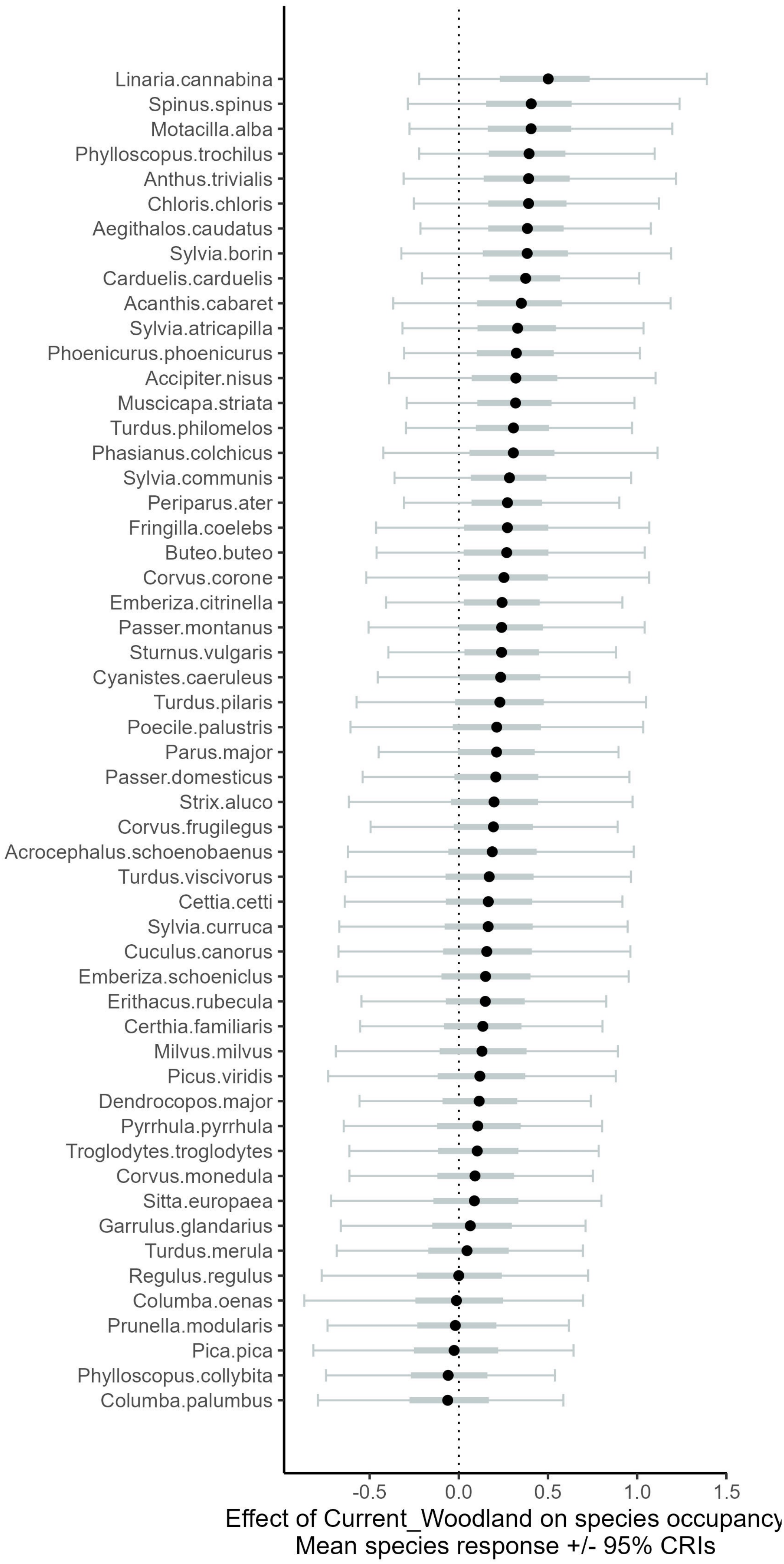

Linaria.cannabina
Spinus.spinus
Motacilla.alba
Phylloscopus.trochilus
Anthus.trivialis
Chloris.chloris
Aegithalos.caudatus
Sylvia.borin
Carduelis.carduelis
Acanthis.cabaret
Sylvia.atricapilla
Phoenicurus.phoenicurus
Accipiter.nisus
Muscicapa.striata
Turdus.philomelos
Phasianus.colchicus
Sylvia.communis
Periparus.ater
Fringilla.coelebs
Buteo.buteo
Corvus.corone
Emberiza.citrinella
Passer.montanus
Sturnus.vulgaris
Cyanistes.caeruleus
Turdus.pilaris
Poecile.palustris
Parus.major
Passer.domesticus
Strix.aluco
Corvus.frugilegus
Acrocephalus.schoenobaenus
Turdus.viscivorus
Cettia.cetti
Sylvia.curruca
Cuculus.canorus
Emberiza.schoeniclus
Erithacus.rubecula
Certhia.familiaris
Milvus.milvus
Picus.viridis
Dendrocopos.major
Pyrrhula.pyrrhula
Troglodytes.troglodytes
Corvus.monedula
Sitta.europaea
Garrulus.glandarius
Turdus.merula
Regulus.regulus
Columba.oenas
Prunella.modularis
Pica.pica
Phylloscopus.collybita
Columba.palumbus
-0.5
0.0
0.5
1.0
1.5
Effect of Current_Woodland on species occupancy
Mean species response +/- 95% CRIs

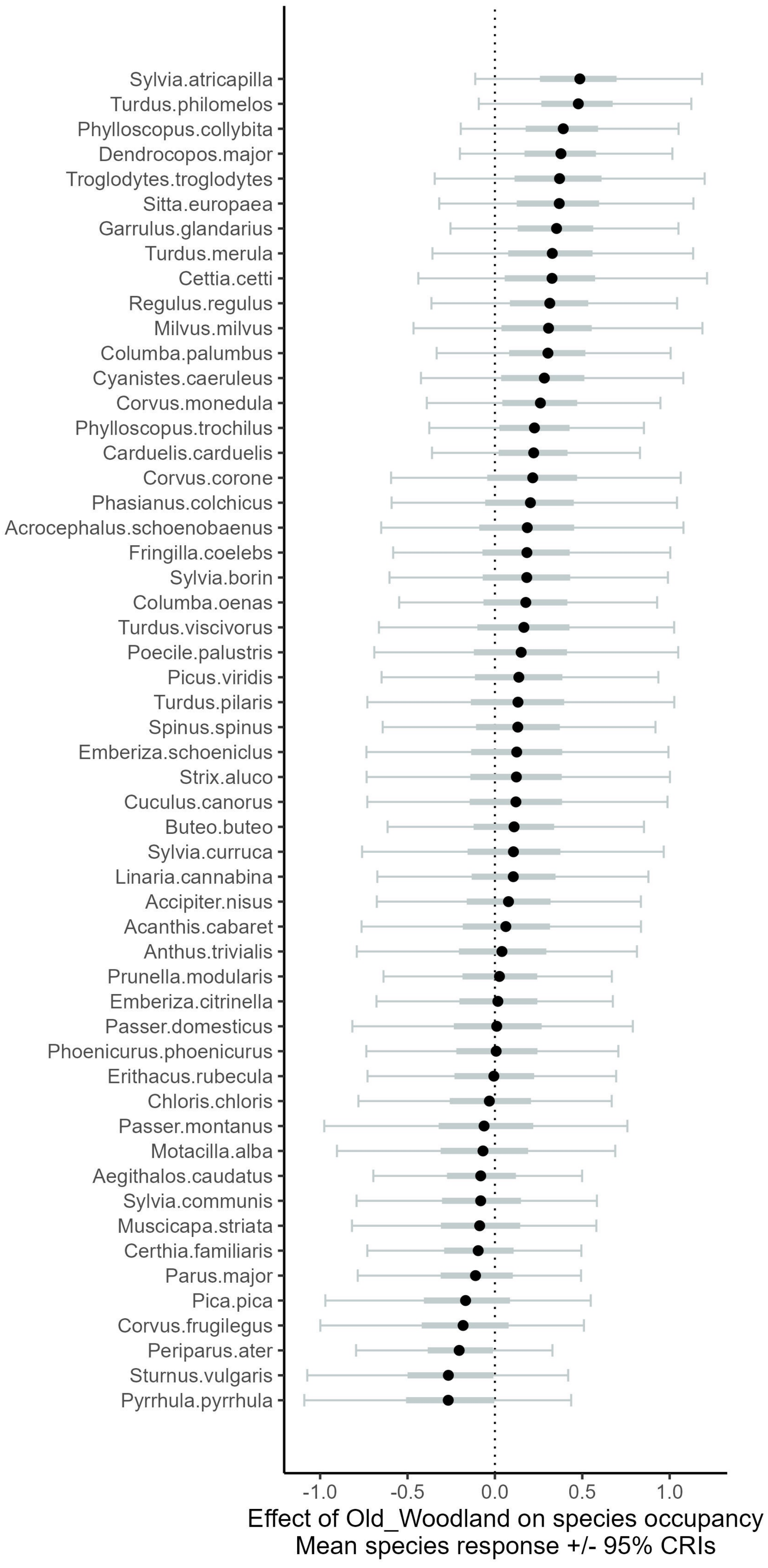

Sylvia.atricapilla
Turdus.philomelos
Phylloscopus.collybita
Dendrocopos.major
Troglodytes.troglodytes
Sitta.europaea
Garrulus.glandarius
Turdus.merula
Cettia.cetti
Regulus.regulus
Milvus.milvus
Columba.palumbus
Cyanistes.caeruleus
Corvus.monedula
Phylloscopus.trochilus
Carduelis.carduelis
Corvus.corone
Phasianus.colchicus
Acrocephalus.schoenobaenus
Fringilla.coelebs
Sylvia.borin
Columba.oenas
Turdus.viscivorus
Poecile.palustris
Picus.viridis
Turdus.pilaris
Spinus.spinus
Emberiza.schoeniclus
Strix.aluco
Cuculus.canorus
Buteo.buteo
Sylvia.curruca
Linaria.cannabina
Accipiter.nisus
Acanthis.cabaret
Anthus.trivialis
Prunella.modularis
Emberiza.citrinella
Passer.domesticus
Phoenicurus.phoenicurus
Erithacus.rubecula
Chloris.chloris
Passer.montanus
Motacilla.alba
Aegithalos.caudatus
Sylvia.communis
Muscicapa.striata
Certhia.familiaris
Parus.major
Pica.pica
Corvus.frugilegus
Periparus.ater
Sturnus.vulgaris
Pyrrhula.pyrrhula
-1.0
-0.5
0.0
0.5
1.0
Effect of Old_Woodland on species occupancy
Mean species response +/- 95% CRIs

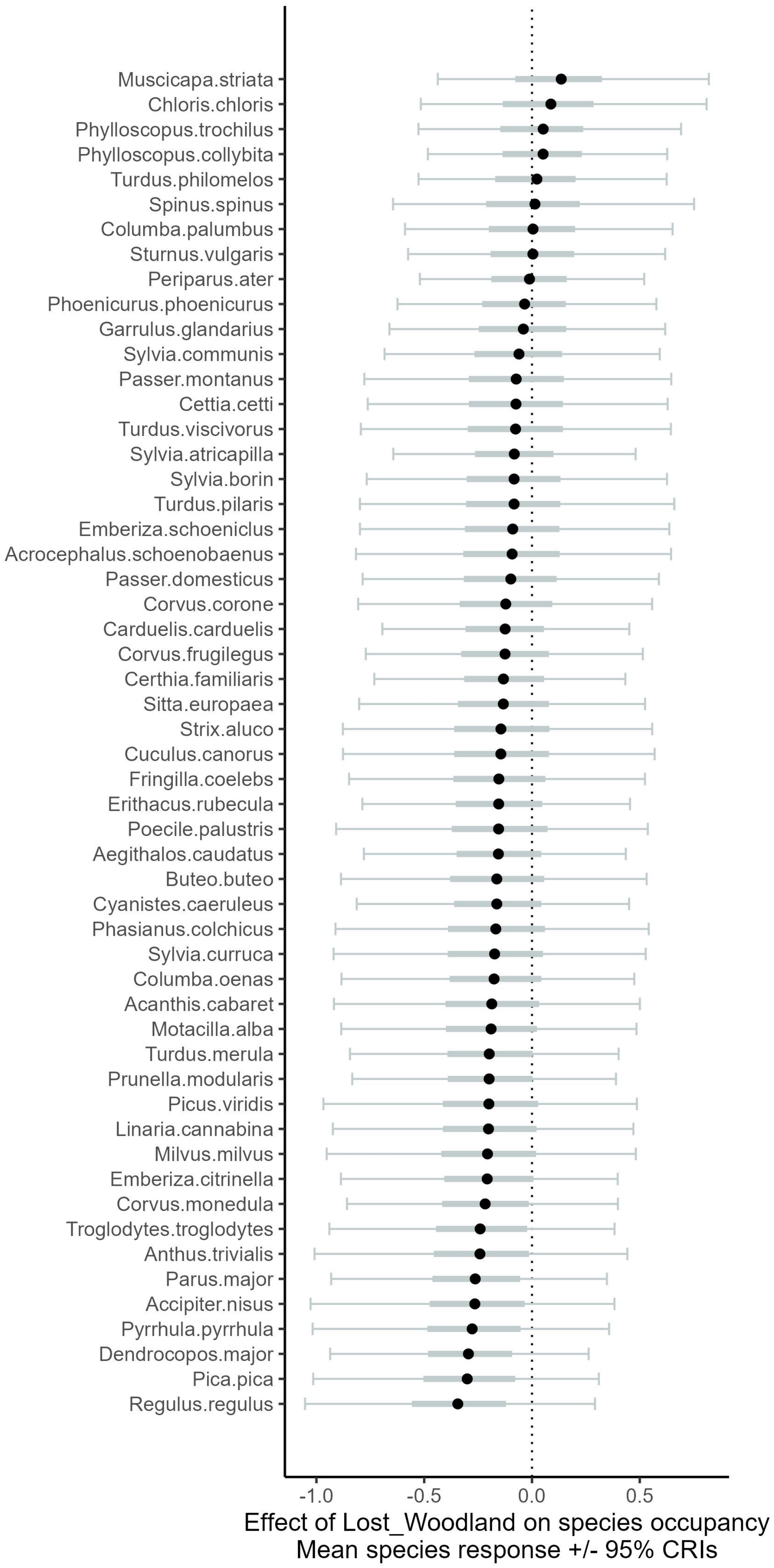

Muscicapa.striata
Chloris.chloris
Phylloscopus.trochilus
Phylloscopus.collybita
Turdus.philomelos
Spinus.spinus
Columba.palumbus
Sturnus.vulgaris
Periparus.ater
Phoenicurus.phoenicurus
Garrulus.glandarius
Sylvia.communis
Passer.montanus
Cettia.cetti
Turdus.viscivorus
Sylvia.atricapilla
Sylvia.borin
Turdus.pilaris
Emberiza.schoeniclus
Acrocephalus.schoenobaenus
Passer.domesticus
Corvus.corone
Carduelis.carduelis
Corvus.frugilegus
Certhia.familiaris
Sitta.europaea
Strix.aluco
Cuculus.canorus
Fringilla.coelebs
Erithacus.rubecula
Poecile.palustris
Aegithalos.caudatus
Buteo.buteo
Cyanistes.caeruleus
Phasianus.colchicus
Sylvia.curruca
Columba.oenas
Acanthis.cabaret
Motacilla.alba
Turdus.merula
Prunella.modularis
Picus.viridis
Linaria.cannabina
Milvus.milvus
Emberiza.citrinella
Corvus.monedula
Troglodytes.troglodytes
Anthus.trivialis
Parus.major
Accipiter.nisus
Pyrrhula.pyrrhula
Dendrocopos.major
Pica.pica
Regulus.regulus
-1.0
-0.5
0.0
0.5
Effect of Lost_Woodland on species occupancy
Mean species response +/- 95% CRIs

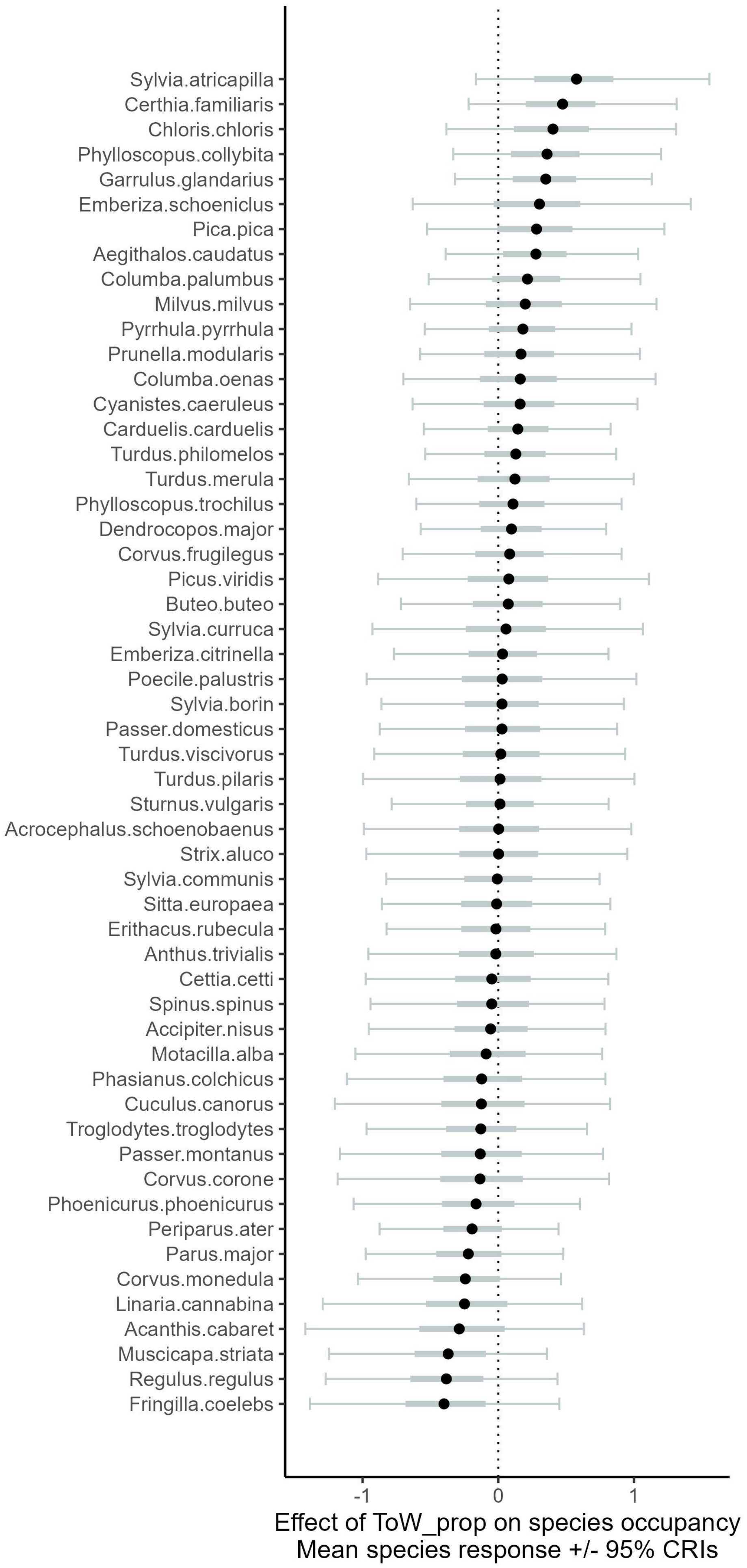
Sylvia.atricapilla
Certhia.familiaris
Chloris.chloris
Phylloscopus.collybita
Garrulus.glandarius
Emberiza.schoeniclus
Pica.pica
Aegithalos.caudatus
Columba.palumbus
Milvus.milvus
Pyrrhula.pyrrhula
Prunella.modularis
Columba.oenas
Cyanistes.caeruleus
Carduelis.carduelis
Turdus.philomelos
Turdus.merula
Phylloscopus.trochilus
Dendrocopos.major
Corvus.frugilegus
Picus.viridis
Buteo.buteo
Sylvia.curruca
Emberiza.citrinella
Poecile.palustris
Sylvia.borin
Passer.domesticus
Turdus.viscivorus
Turdus.pilaris
Sturnus.vulgaris
Acrocephalus.schoenobaenus
Strix.aluco
Sylvia.communis
Sitta.europaea
Erithacus.rubecula
Anthus.trivialis
Cettia.cetti
Spinus.spinus
Accipiter.nisus
Motacilla.alba
Phasianus.colchicus
Cuculus.canorus
Troglodytes.troglodytes
Passer.montanus
Corvus.corone
Phoenicurus.phoenicurus
Periparus.ater
Parus.major
Corvus.monedula
Linaria.cannabina
Acanthis.cabaret
Muscicapa.striata
Regulus.regulus
Fringilla.coelebs
-1
0
1
Effect of ToW_prop on species occupancy
Mean species response +/- 95% CRIs

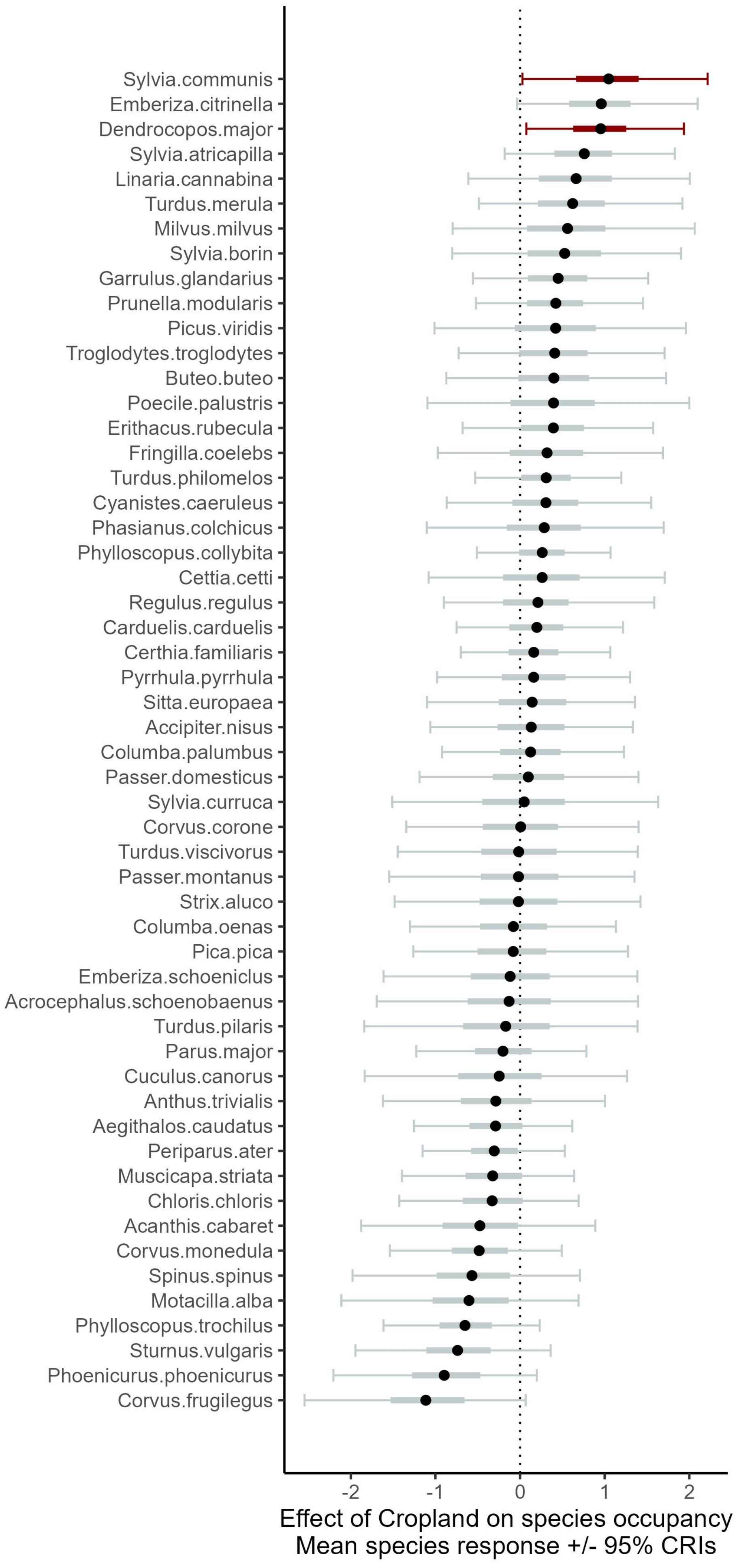
Sylvia.communis
Emberiza.citrinella
Dendrocopos.major
Sylvia.atricapilla
Linaria.cannabina
Turdus.merula
Milvus.milvus
Sylvia.borin
Garrulus.glandarius
Prunella.modularis
Picus.viridis
Troglodytes.troglodytes
Buteo.buteo
Poecile.palustris
Erithacus.rubecula
Fringilla.coelebs
Turdus.philomelos
Cyanistes.caeruleus
Phasianus.colchicus
Phylloscopus.collybita
Cettia.cetti
Regulus.regulus
Carduelis.carduelis
Certhia.familiaris
Pyrrhula.pyrrhula
Sitta.europaea
Accipiter.nisus
Columba.palumbus
Passer.domesticus
Sylvia.curruca
Corvus.corone
Turdus.viscivorus
Passer.montanus
Strix.aluco
Columba.oenas
Pica.pica
Emberiza.schoeniclus
Acrocephalus.schoenobaenus
Turdus.pilaris
Parus.major
Cuculus.canorus
Anthus.trivialis
Aegithalos.caudatus
Periparus.ater
Muscicapa.striata
Chloris.chloris
Acanthis.cabaret
Corvus.monedula
Spinus.spinus
Motacilla.alba
Phylloscopus.trochilus
Sturnus.vulgaris
Phoenicurus.phoenicurus
Corvus.frugilegus
-2
-1
0
1
2
Effect of Cropland on species occupancy
Mean species response +/- 95% CRIs

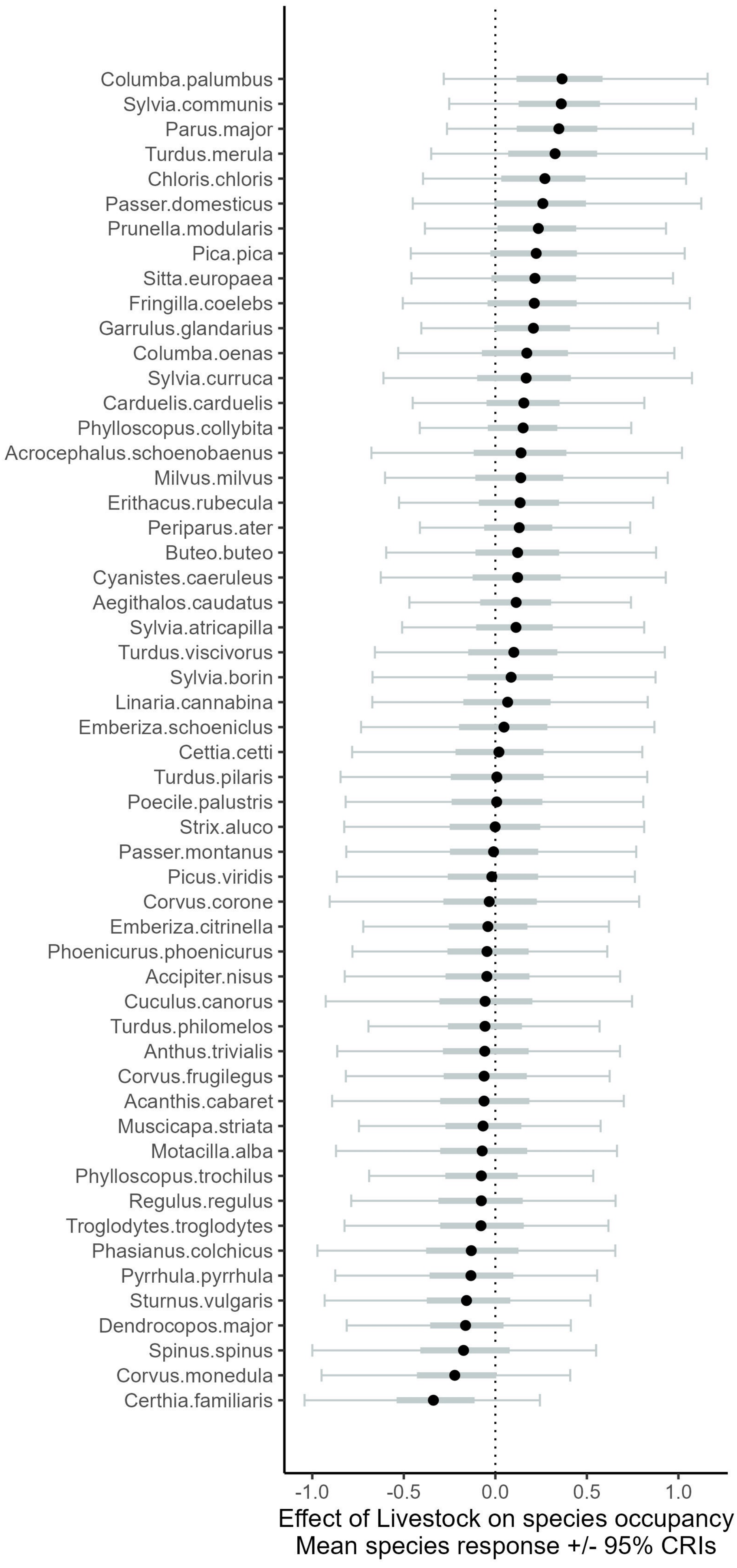
Columba.palumbus
Sylvia.communis
Parus.major
Turdus.merula
Chloris.chloris
Passer.domesticus
Prunella.modularis
Pica.pica
Sitta.europaea
Fringilla.coelebs
Garrulus.glandarius
Columba.oenas
Sylvia.curruca
Carduelis.carduelis
Phylloscopus.collybita
Acrocephalus.schoenobaenus
Milvus.milvus
Erithacus.rubecula
Periparus.ater
Buteo.buteo
Cyanistes.caeruleus
Aegithalos.caudatus
Sylvia.atricapilla
Turdus.viscivorus
Sylvia.borin
Linaria.cannabina
Emberiza.schoeniclus
Cettia.cetti
Turdus.pilaris
Poecile.palustris
Strix.aluco
Passer.montanus
Picus.viridis
Corvus.corone
Emberiza.citrinella
Phoenicurus.phoenicurus
Accipiter.nisus
Cuculus.canorus
Turdus.philomelos
Anthus.trivialis
Corvus.frugilegus
Acanthis.cabaret
Muscicapa.striata
Motacilla.alba
Phylloscopus.trochilus
Regulus.regulus
Troglodytes.troglodytes
Phasianus.colchicus
Pyrrhula.pyrrhula
Sturnus.vulgaris
Dendrocopos.major
Spinus.spinus
Corvus.monedula
Certhia.familiaris
-1.0
-0.5
0.0
0.5
1.0
Effect of Livestock on species occupancy
Mean species response +/- 95% CRIs

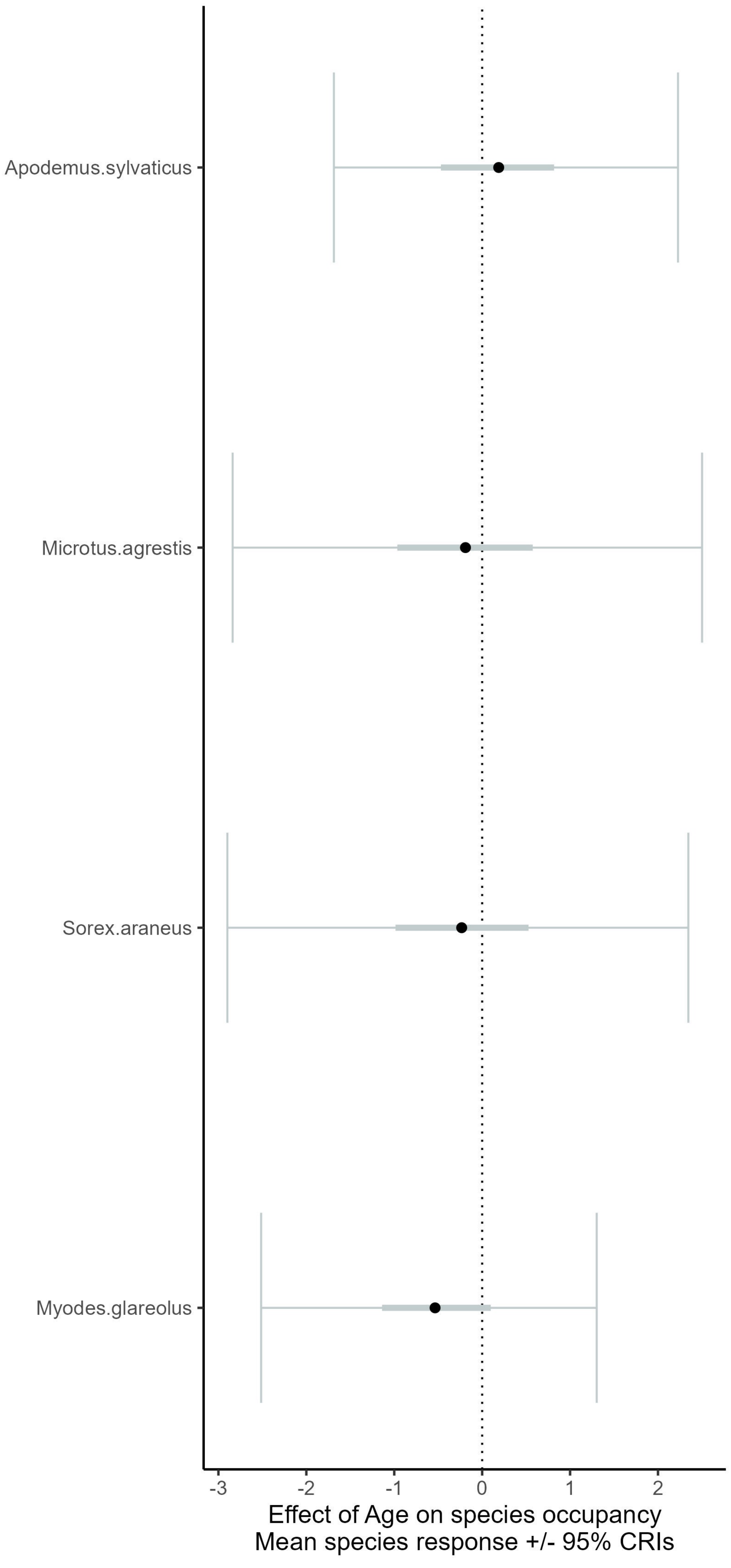

Apodemus.sylvaticus
Microtus.agrestis
Sorex.araneus
Myodes.glareolus
-3
-2
-1
0
1
2
Effect of Age on species occupancy
Mean species response +/- 95% CRIs

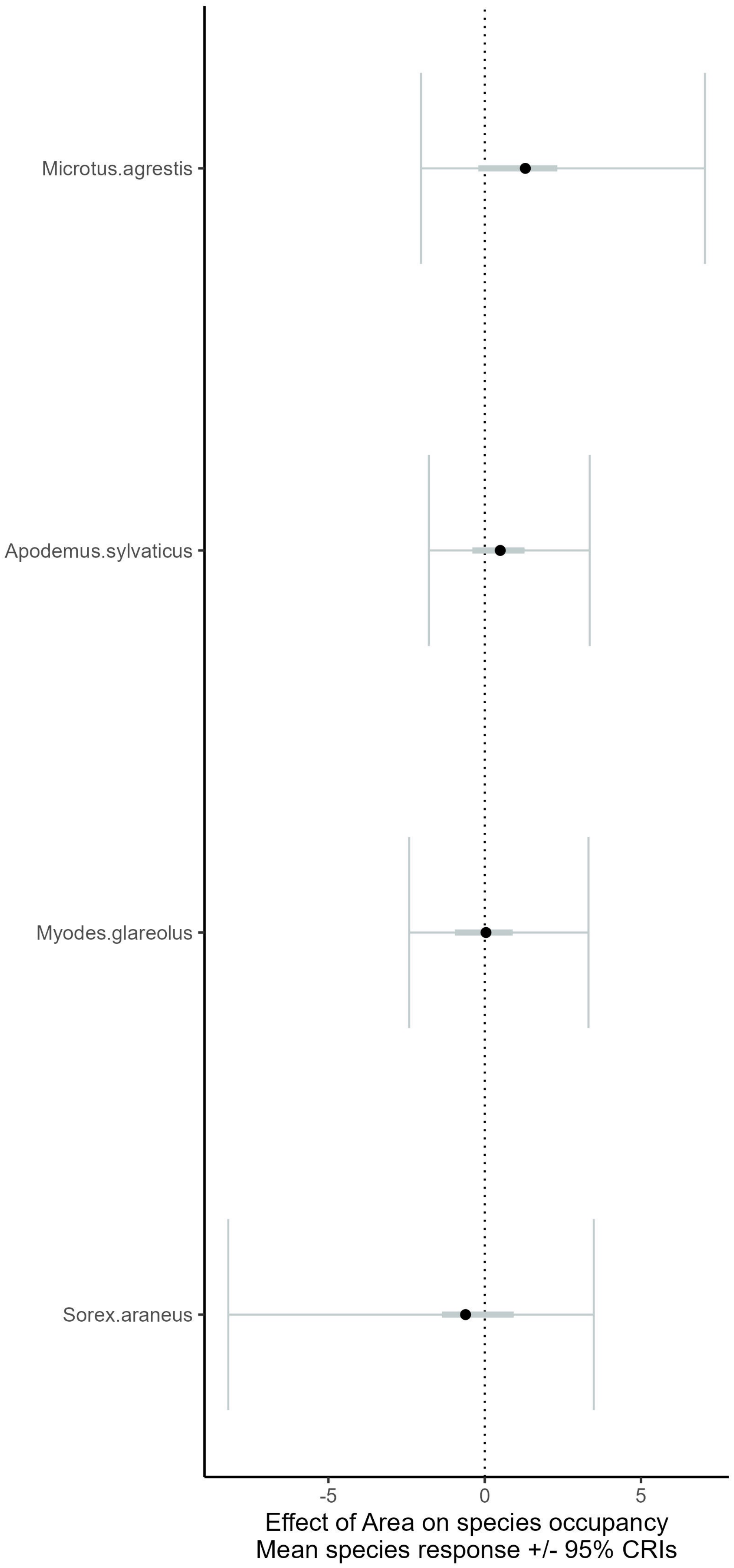

Microtus.agrestis
Apodemus.sylvaticus
Myodes.glareolus
Sorex.araneus
-5
0
5
Effect of Area on species occupancy
Mean species response +/- 95% CRIs

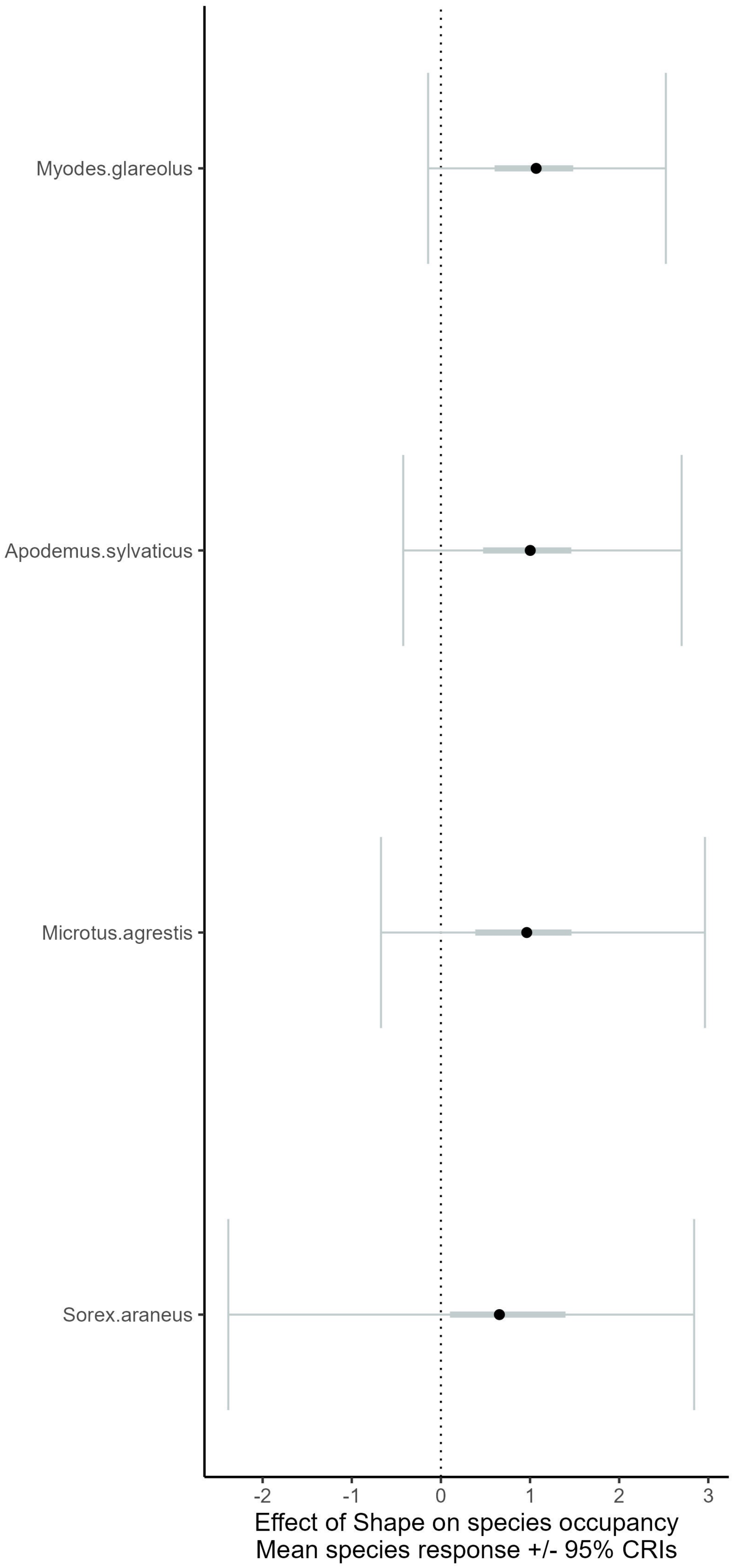
Myodes.glareolus
Apodemus.sylvaticus
Microtus.agrestis
Sorex.araneus
-2
-1
0
1
2
3
Effect of Shape on species occupancy
Mean species response +/- 95% CRIs

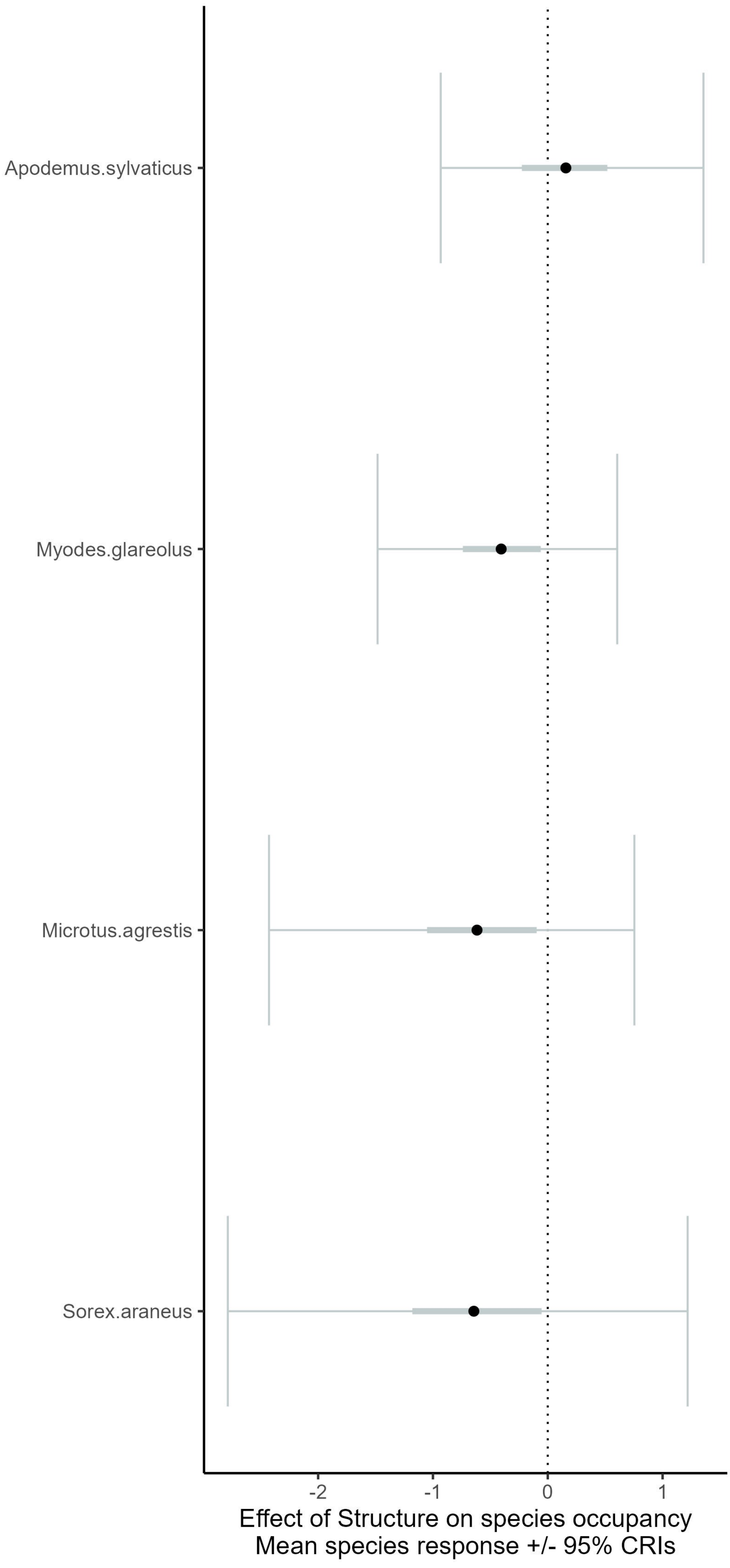
Apodemus.sylvaticus
Myodes.glareolus
Microtus.agrestis
Sorex.araneus
-2
-1
0
1
Effect of Structure on species occupancy
Mean species response +/- 95% CRIs

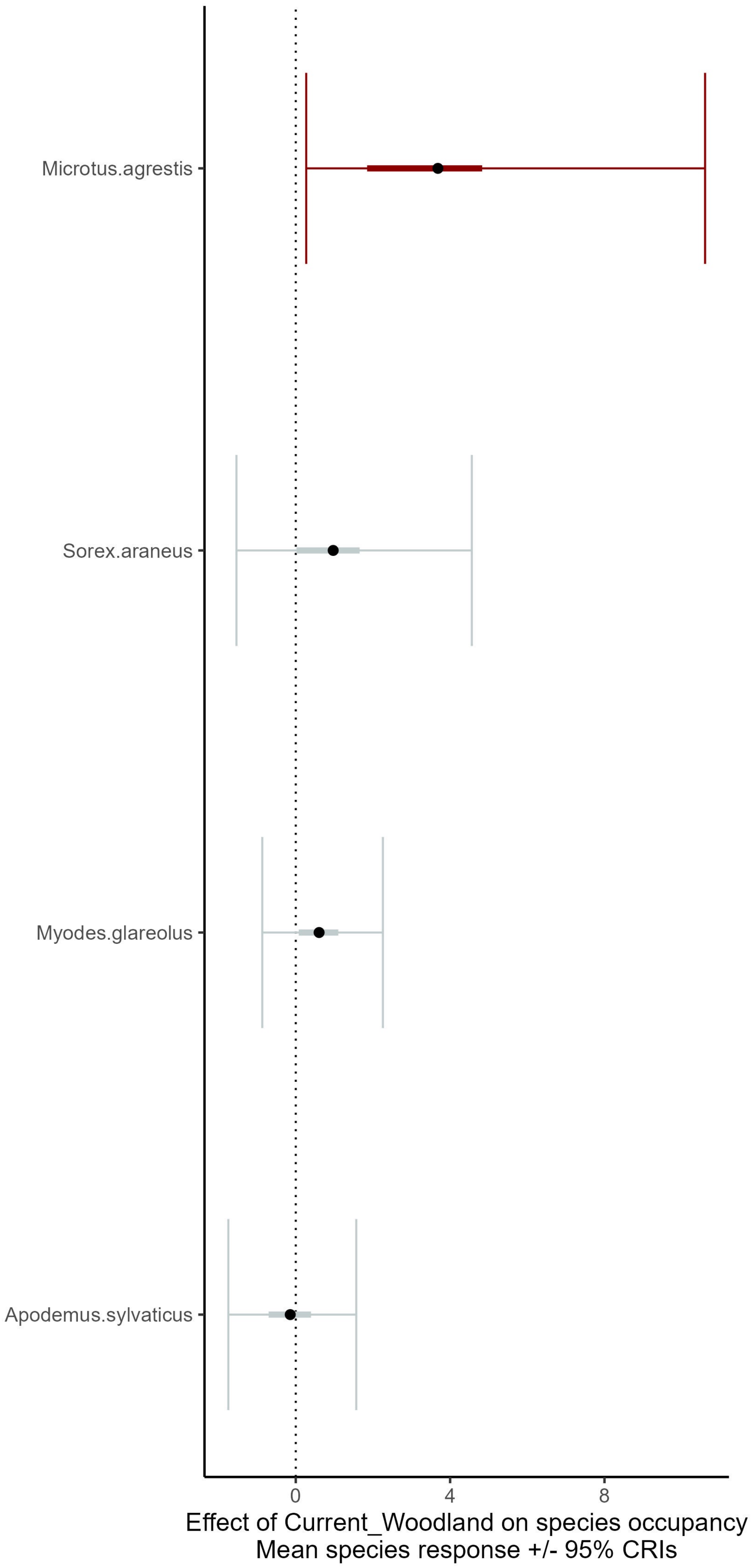
Microtus.agrestis
Sorex.araneus
Myodes.glareolus
Apodemus.sylvaticus
0
4
8
Effect of Current_Woodland on species occupancy
Mean species response +/- 95% CRIs

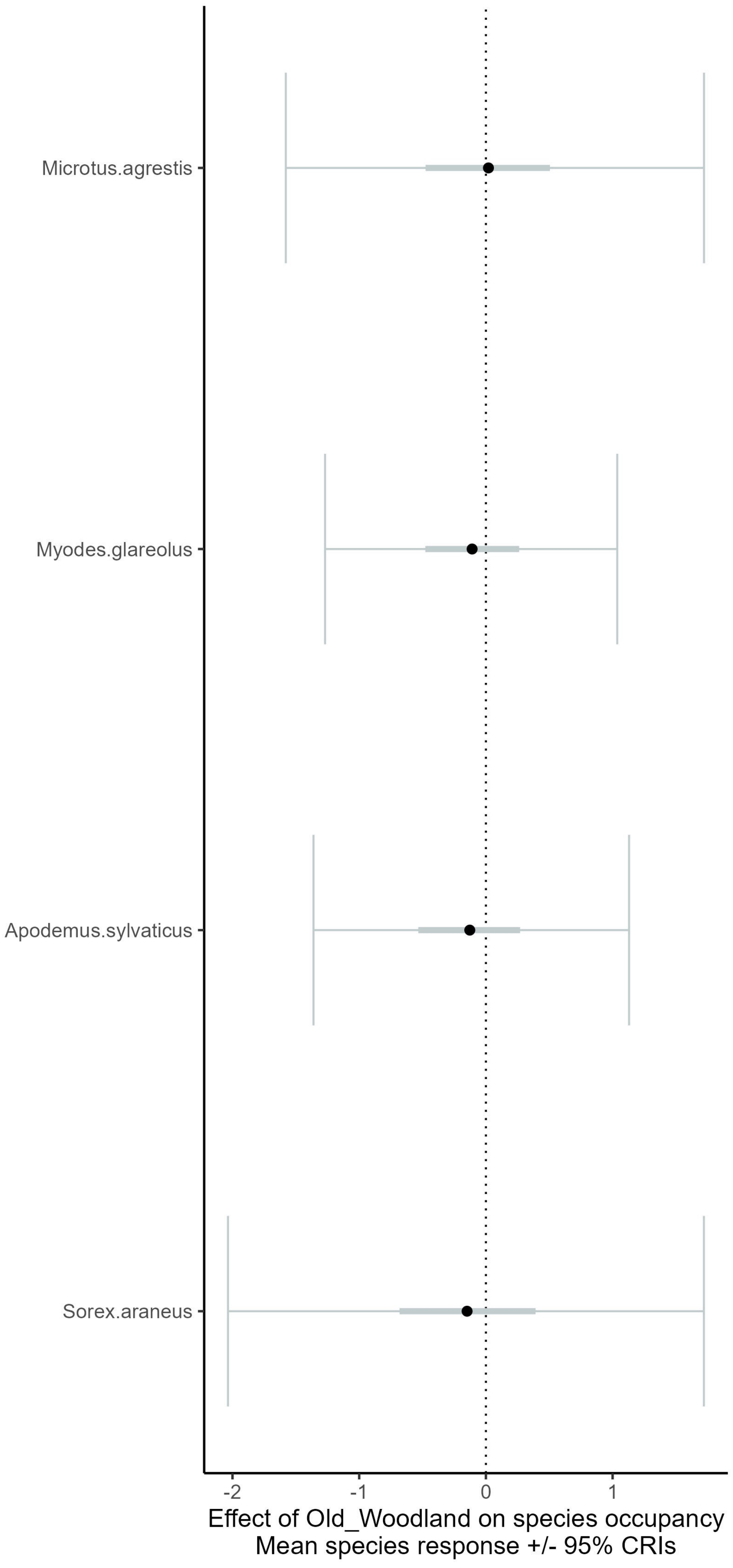

Microtus.agrestis
Myodes.glareolus
Apodemus.sylvaticus
Sorex.araneus
-2
-1
0
1
Effect of Old_Woodland on species occupancy
Mean species response +/- 95% CRIs

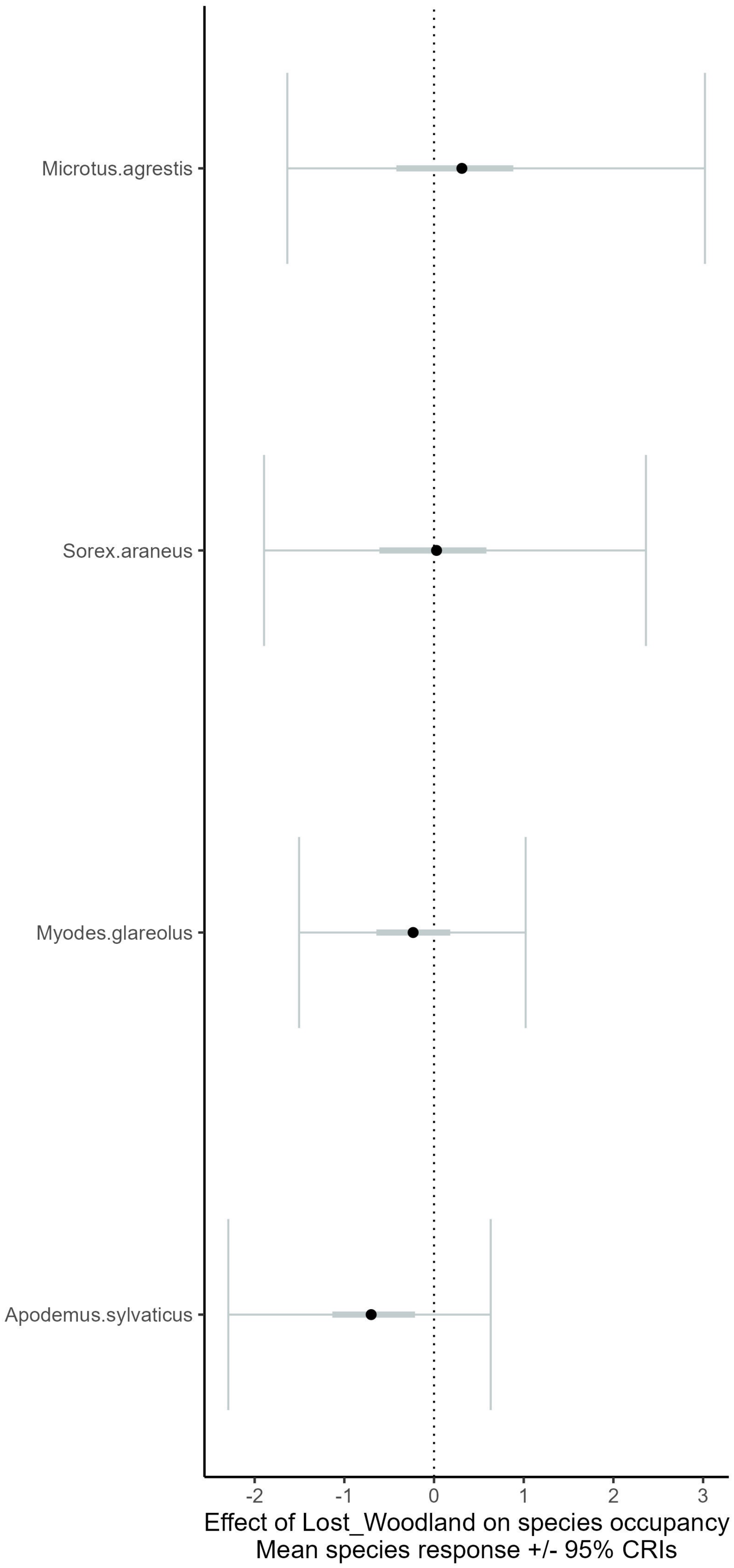
Microtus.agrestis
Sorex.araneus
Myodes.glareolus
Apodemus.sylvaticus
-2
-1
0
1
2
3
Effect of Lost_Woodland on species occupancy
Mean species response +/- 95% CRIs

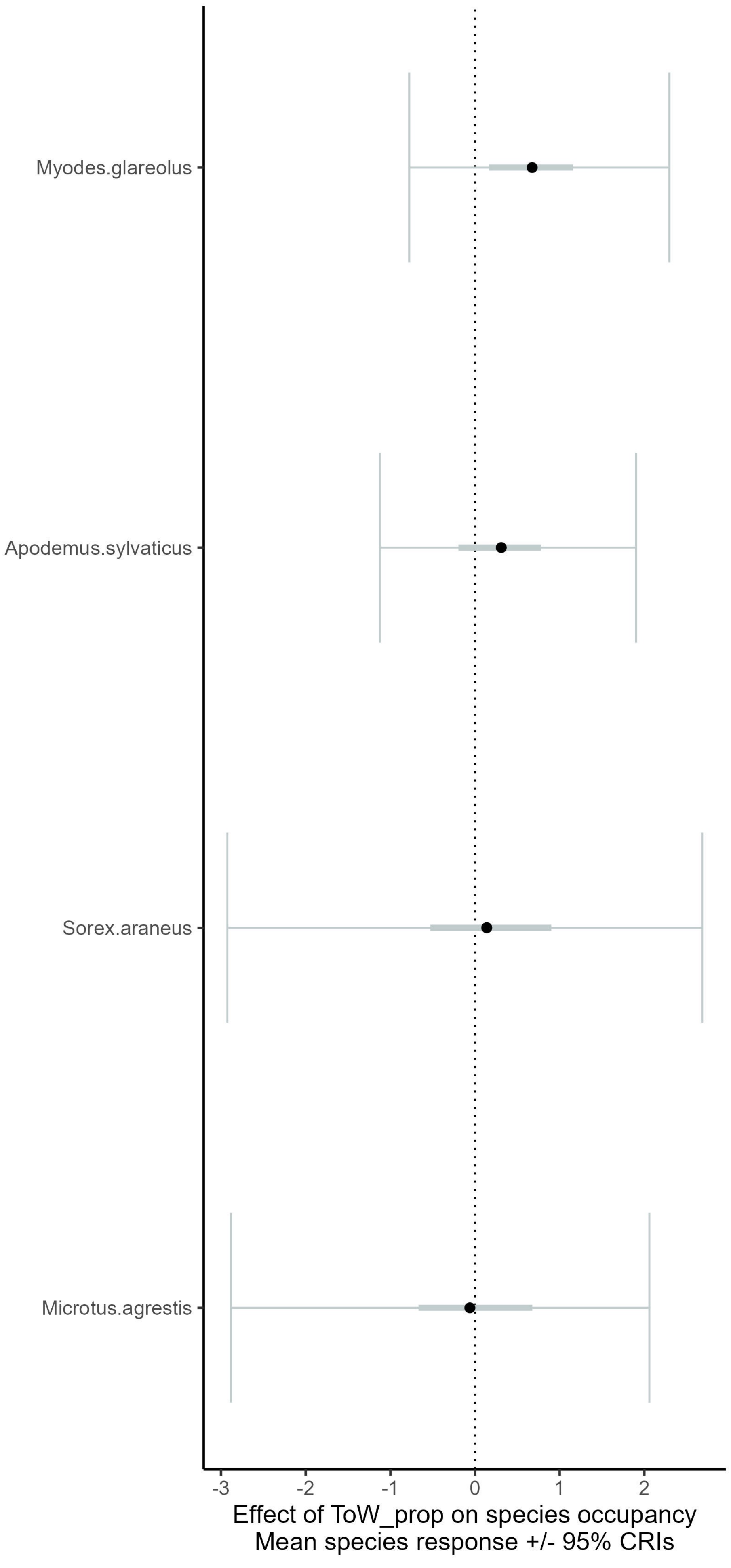

Myodes.glareolus
Apodemus.sylvaticus
Sorex.araneus
Microtus.agrestis
-3
-2
-1
0
1
2
Effect of ToW_prop on species occupancy
Mean species response +/- 95% CRIs

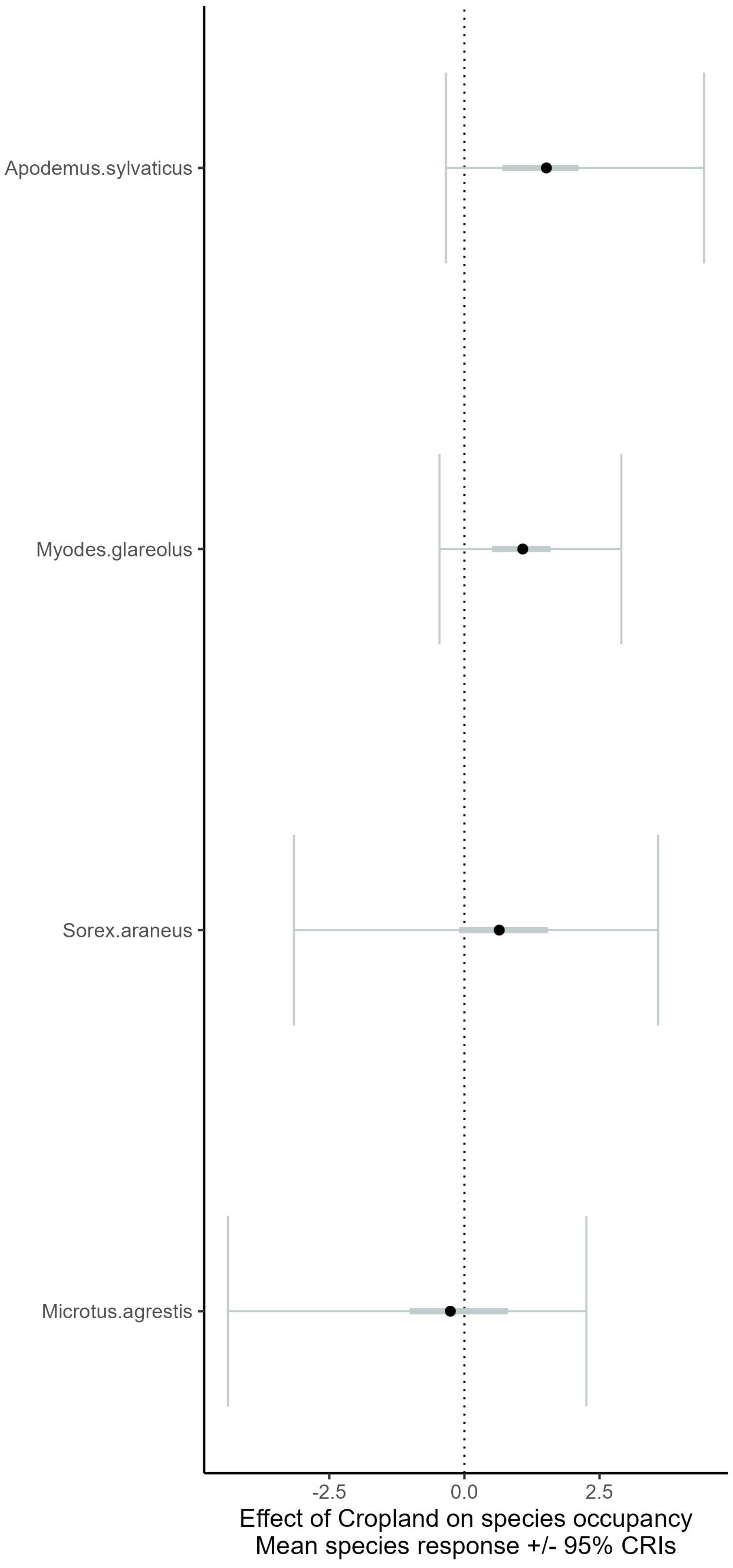
Apodemus.sylvaticus
Myodes.glareolus
Sorex.araneus
Microtus.agrestis
-2.5
0.0
2.5
Effect of Cropland on species occupancy
Mean species response +/- 95% CRIs

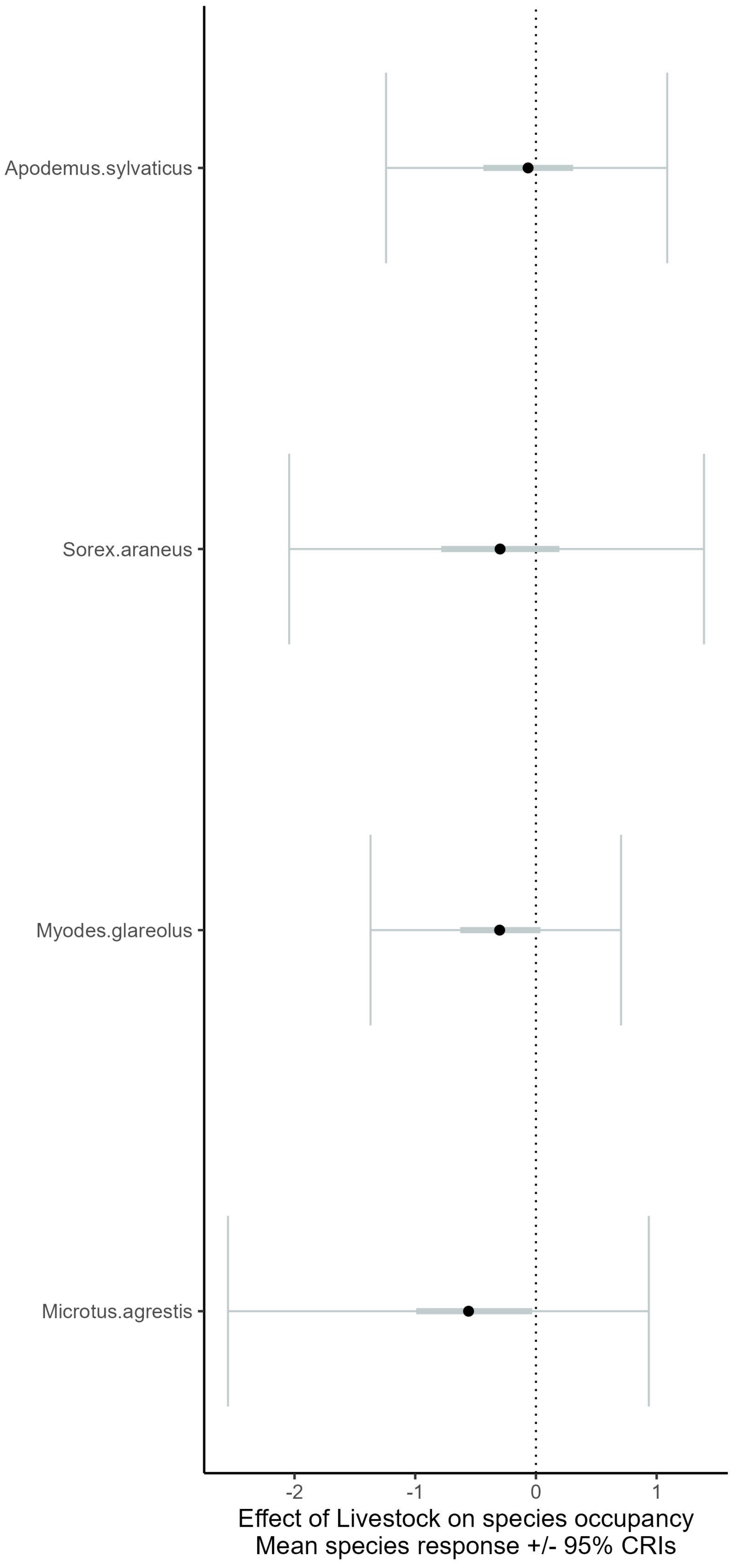
Apodemus.sylvaticus
Sorex.araneus
Myodes.glareolus
Microtus.agrestis
-2
-1
0
1
Effect of Livestock on species occupancy
Mean species response +/- 95% CRIs

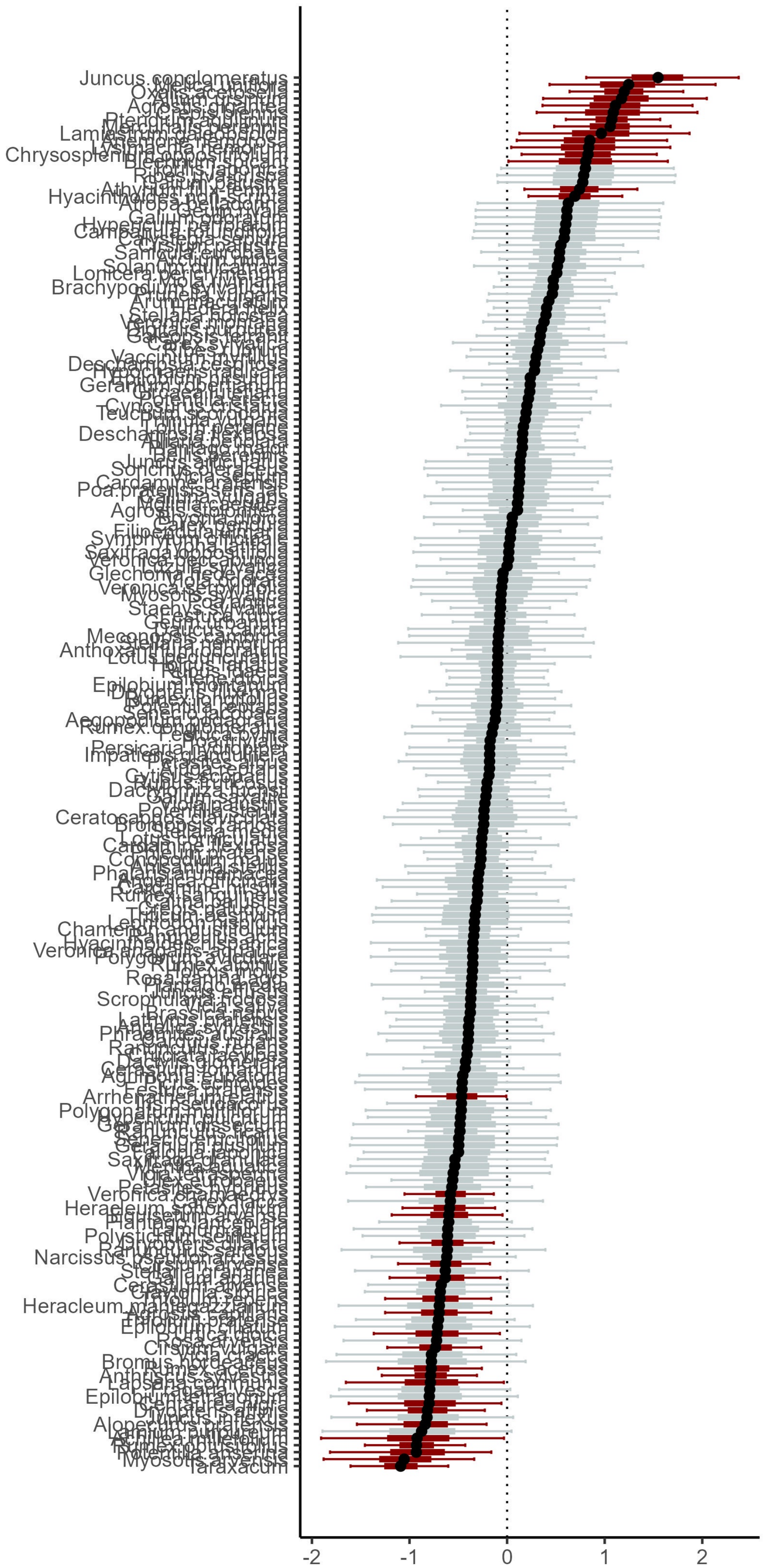

Juncus.conglomeratus
Taraxacum
-2
-1
0
1
2
Effect of Age on species occupancy
Mean species response +/- 95% CRIs

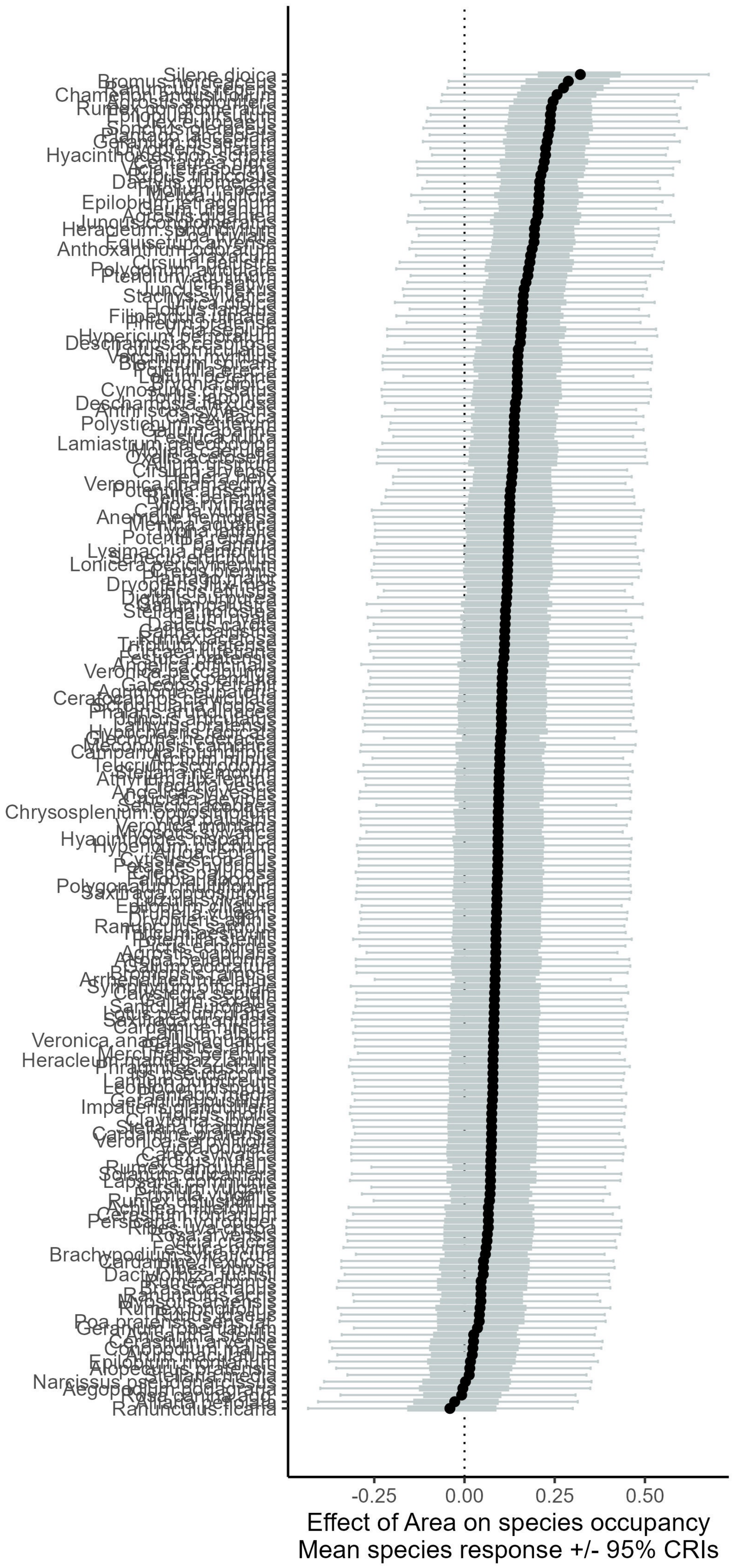

Silene dioica
Ranunculus.ficaria
-0.25
0.00
0.25
0.50
Effect of Area on species occupancy
Mean species response +/- 95% CRIs

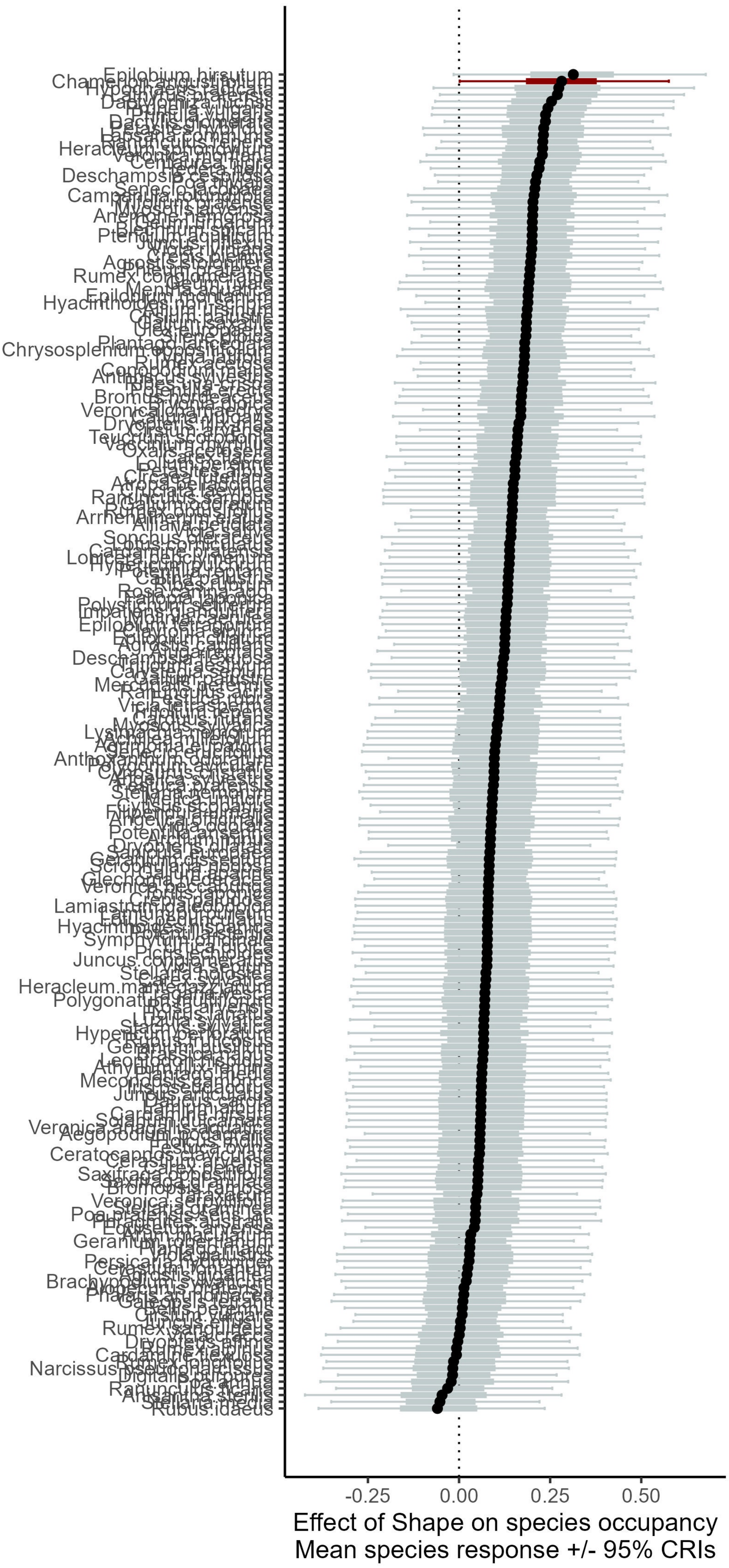

Epilobium.hirsutum
Rubus.idaeus
-0.25
0.00
0.25
0.50
Effect of Shape on species occupancy
Mean species response +/- 95% CRIs

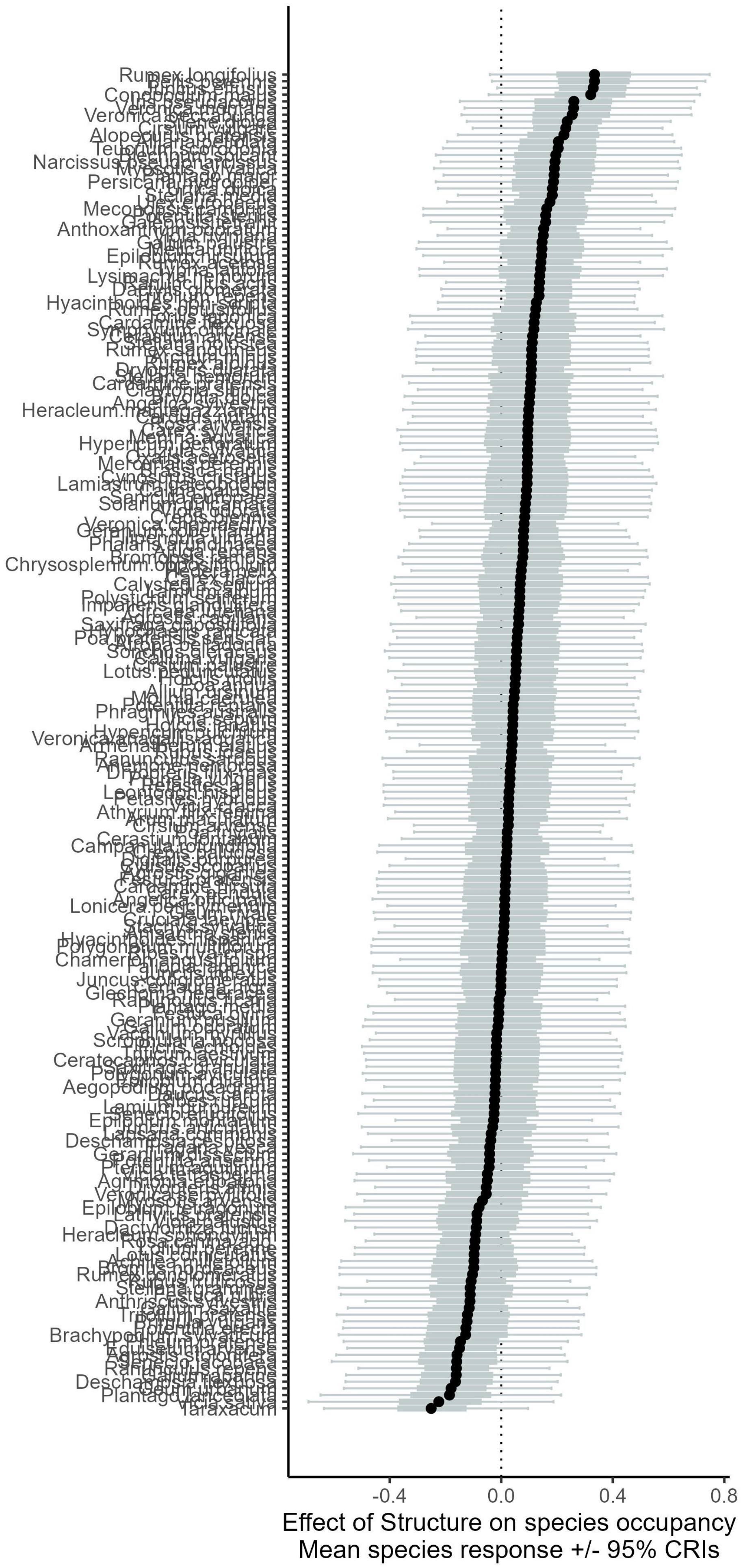
Rumex.longifolius
Taraxacum
-0.4
0.0
0.4
0.8
Effect of Structure on species occupancy
Mean species response +/- 95% CRIs

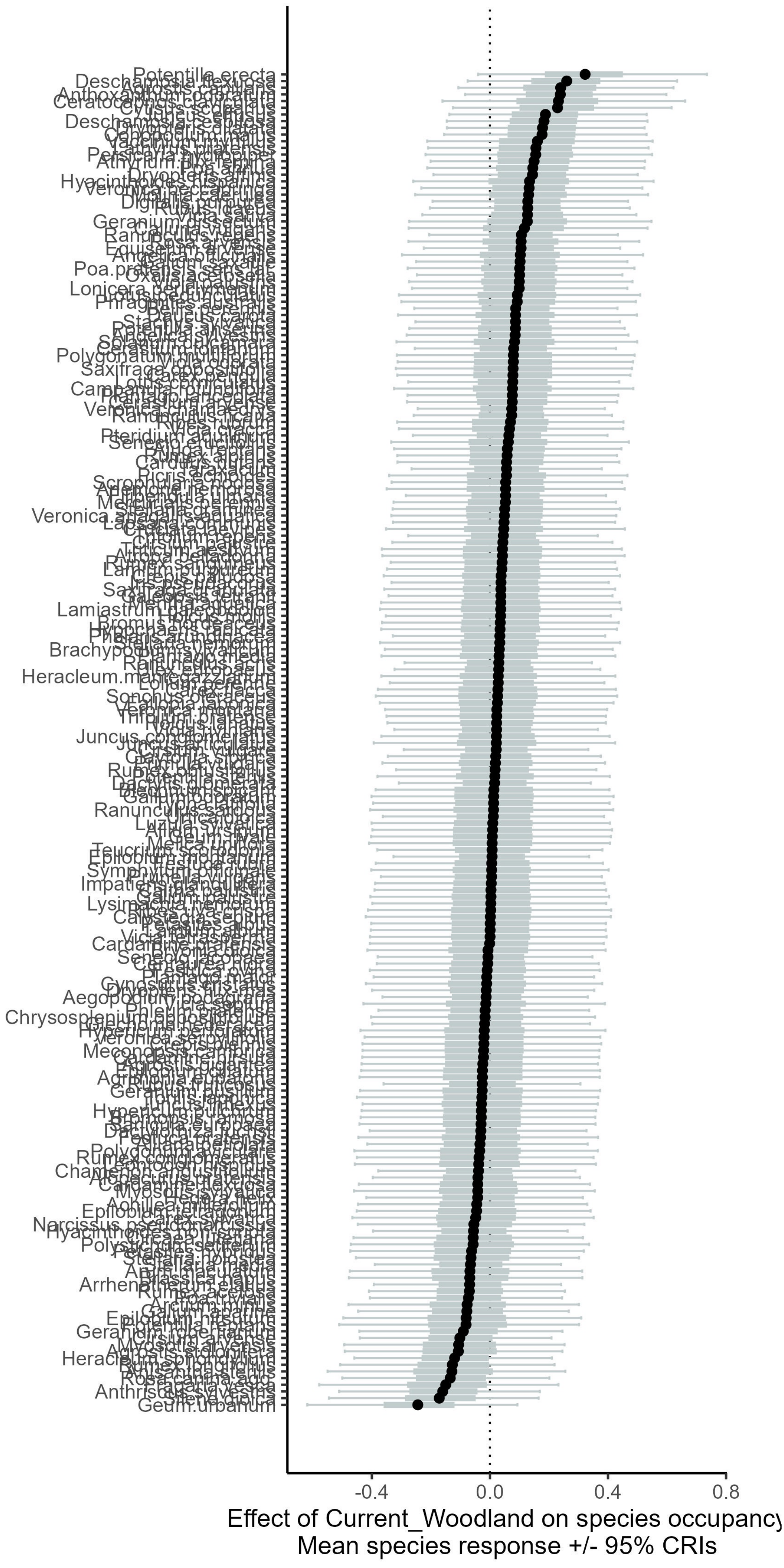

Potentilla.erecta
Geum.urbanum
-0.4
0.0
0.4
0.8
Effect of Current_Woodland on species occupancy
Mean species response +/- 95% CRIs

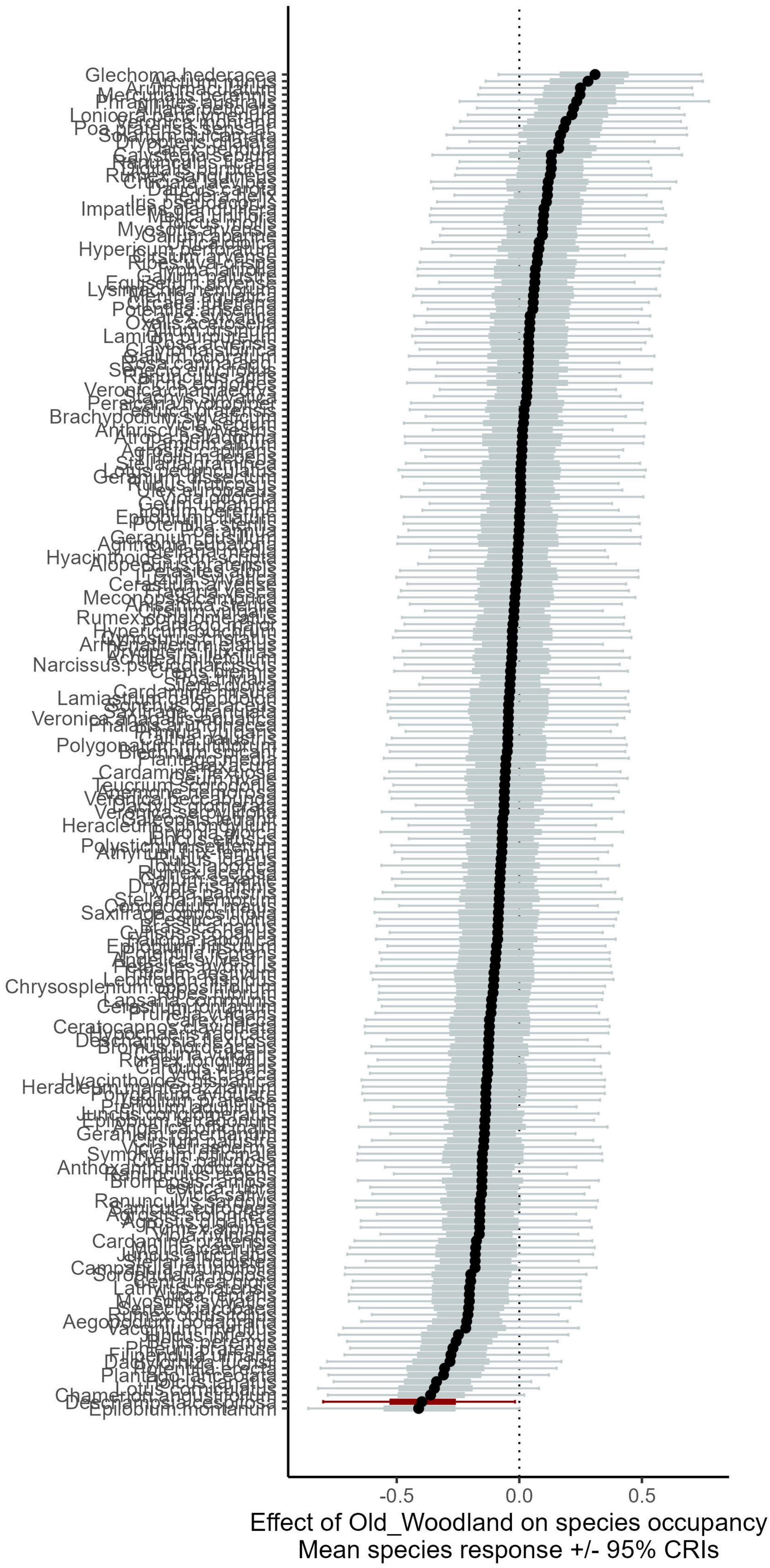
Glechoma.hederacea
Narcissus.pseudonarcissus
Chrysosplenium.oppositifolium
Epilobium.montanum
-0.5
0.0
0.5
Effect of Old_Woodland on species occupancy
Mean species response +/- 95% CRIs

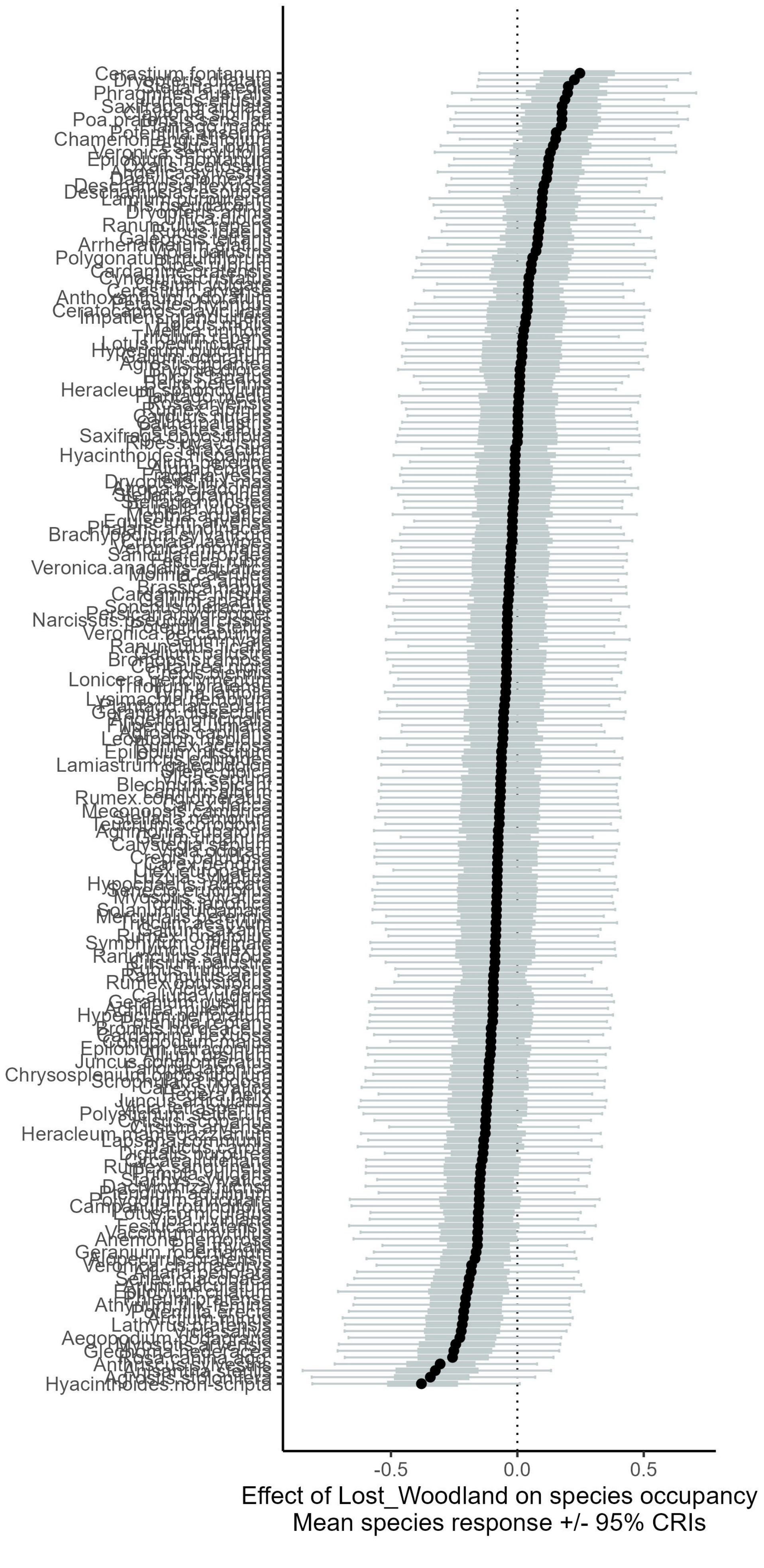

Cerastium.fontanum
Hyacinthoides.non-scripta
-0.5
0.0
0.5
Effect of Lost_Woodland on species occupancy
Mean species response +/- 95% CRIs

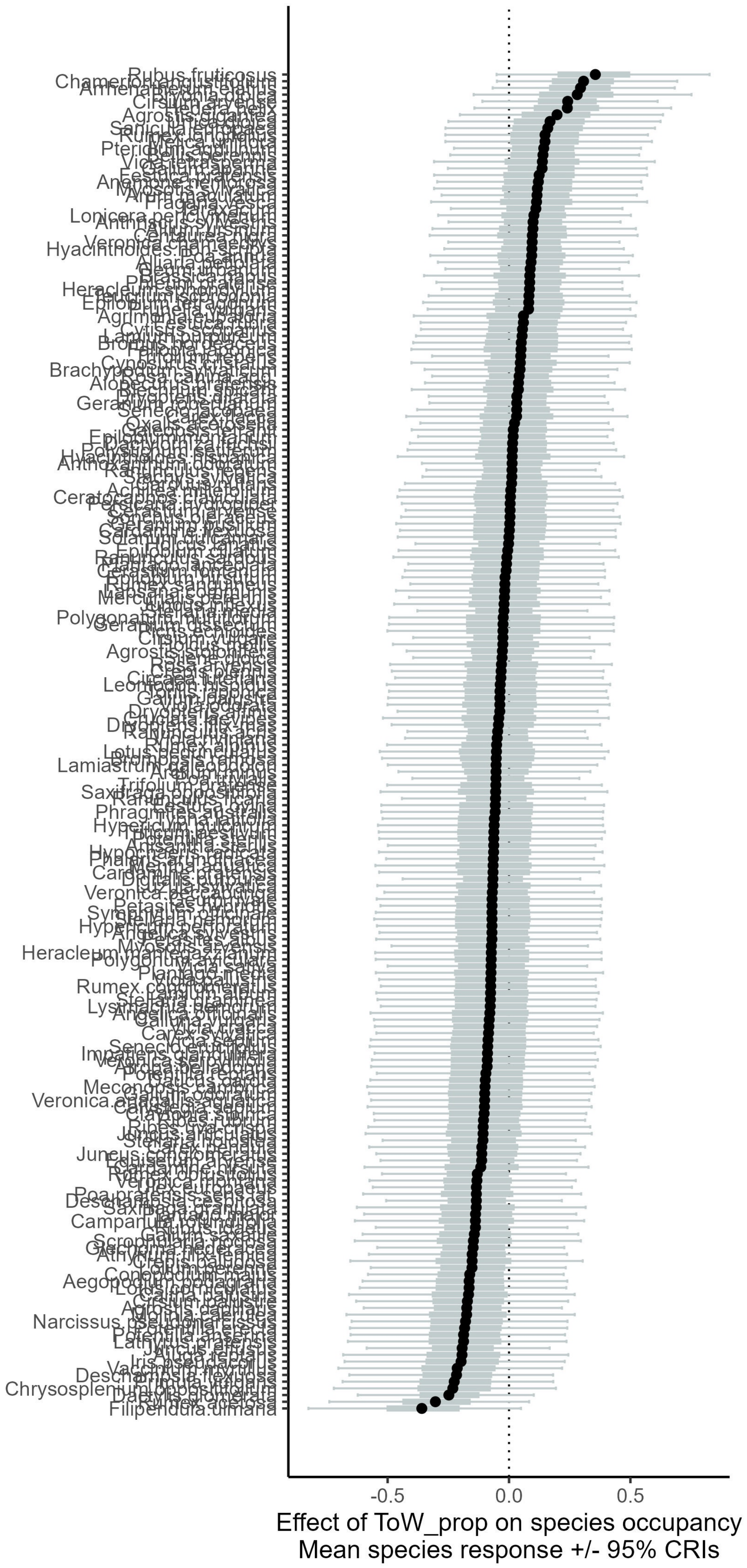
Rubus.fruticosus
Filipendula.ulmaria
-0.5
0.0
0.5
Effect of ToW_prop on species occupancy
Mean species response +/- 95% CRIs

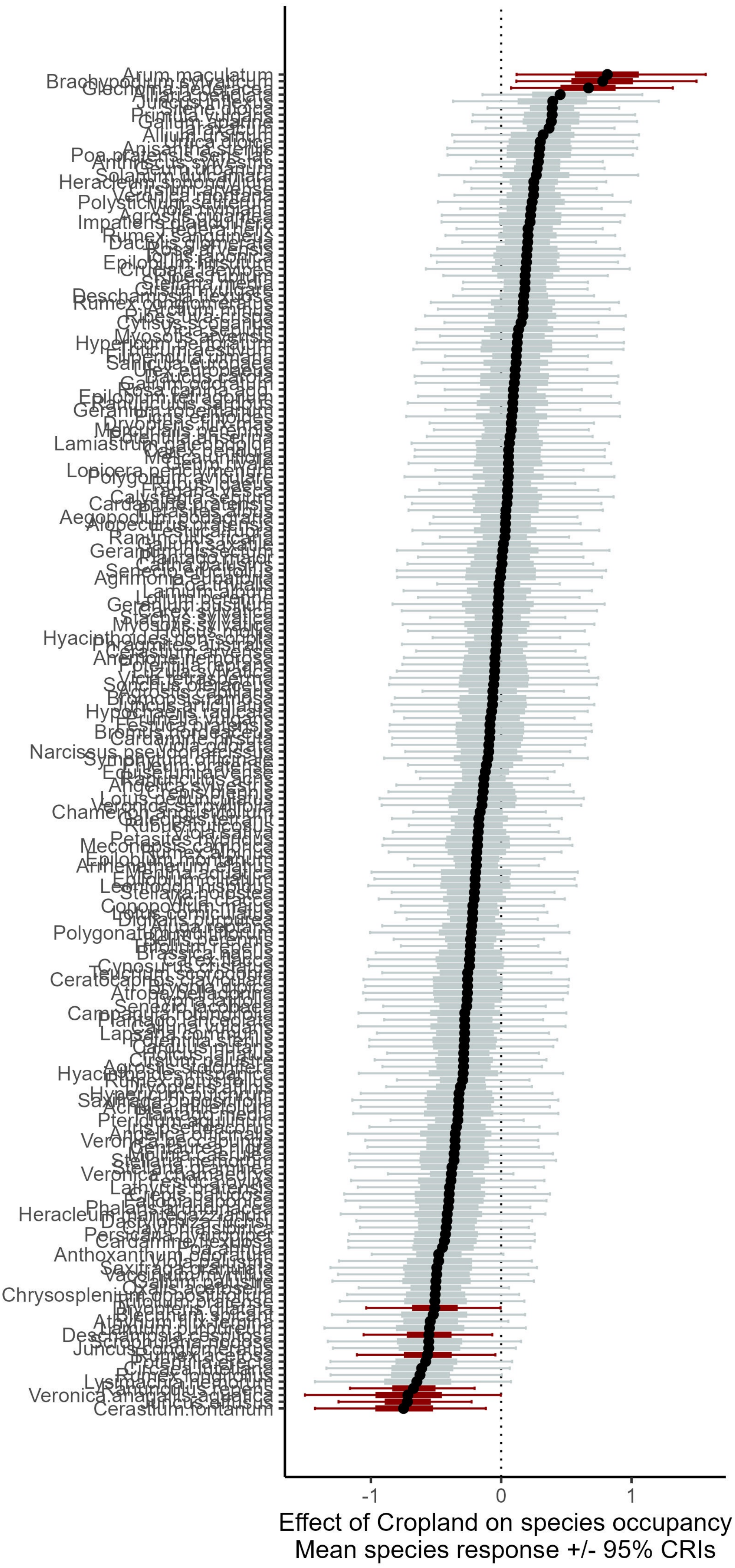

Arum maculatum
Cerastium fontanum
-1
0
1
Effect of Cropland on species occupancy
Mean species response +/- 95% CRIs

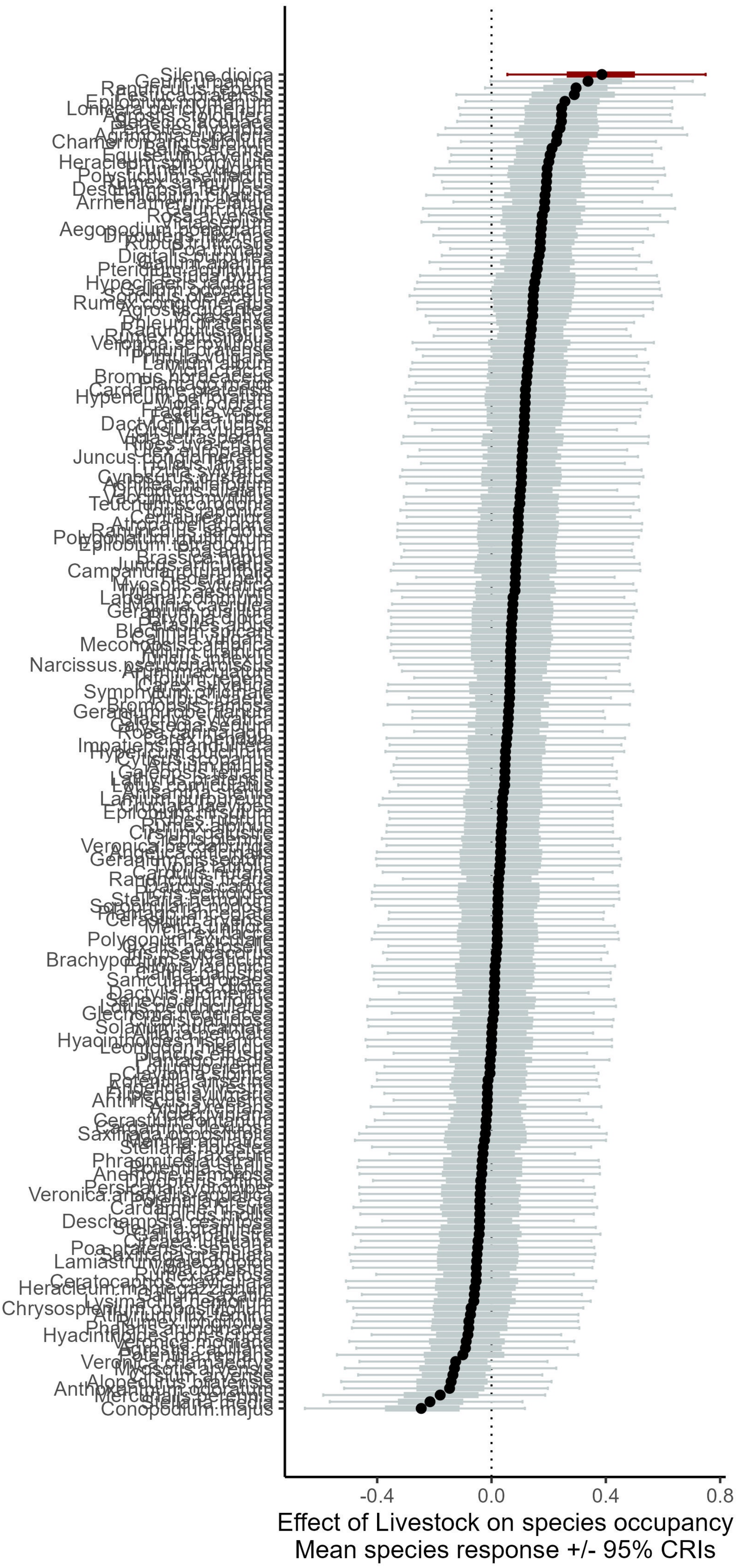

Silene.dioica
Narcissus.pseudonarcissus
Conopodium.majus
-0.4
0.0
0.4
0.8
Effect of Livestock on species occupancy
Mean species response +/- 95% CRIs

Supplementary Information References

Benítez-López, A., Alkemade, R., & Verweij, P. A. (2010). The impacts of roads and other infrastructure on mammal and bird populations: A meta-analysis. Biological Conservation, 143(6), 1307-1316. https://doi.org/10.1016/j.biocon.2010.02.009

Chamberlain, D. E., Fuller, R. J., Bunce, R. G. H., Duckworth, J. C., & Shrubb, M. (2000). Changes in the abundance of farmland birds in relation to the timing of agricultural intensification in England and Wales. Journal of Applied Ecology, 37, 771-788.

Cooke, S. C., Balmford, A., Donald, P. F., Newson, S. E., & Johnston, A. (2020). Roads as a contributor to landscape-scale variation in bird communities. Nature Communications, 11(1), 3125. https://doi.org/10.1038/s41467-020-16899-x

Cooke, S. C., Balmford, A., Johnston, A., Newson, S. E., Donald, P. F., & Villard, M. A. (2020). Variation in abundances of common bird species associated with roads. Journal of Applied Ecology, 57(7), 1271-1282. https://doi.org/10.1111/1365-2664.13614

DEFRA (2024). UK Biodiversity Indicators 2024. Department for Environment, Food and Rural Affairs, UK. https://hub.jncc.gov.uk/assets/7162735c-9fa7-4962-aee7-709d242173f1#UK-BDI-2024-birds-wider-countryside.ods

Doser, J. W., Finley, A. O., Kéry, M., & Zipkin, E. F. (2022). spOccupancy: An R package for single-species, multi-species, and integrated spatial occupancy models. Methods in Ecology and Evolution, 13(8), 1670-1678. https://doi.org/10.1111/2041-210x.13897

EDINA (2023). AgCensus. In: © University of Edinburgh Derived from DEFRA/DAA/RESAS agricultural census surveys.

Forestry Commission (2018). National Forest Inventory. In Available under Open Government Licence. Contains, or is based on information supplied by the Forestry Commission. © Crown copyright and database right 2019 Ordnance Survey [100021242].

Forest Research (2021). Mapping Trees Outside Woodlands and Hedgerows.

Frampton, G. K., & Dorne, J. L. C. M. (2007). The effects on terrestrial invertebrates of reducing pesticide inputs in arable crop edges: a meta-analysis. Journal of Applied Ecology, 44(2), 362-373. https://doi.org/10.1111/j.1365-2664.2007.01277.x

Fuentes-Montemayor, E., Ferryman, M., Watts, K., Macgregor, N. A., Hambly, N., Brennan, S., Coxon, R., Langridge, H., & Park, K. J. (2020). Small mammal responses to long-term

large-scale woodland creation: the influence of local and landscape-level attributes. Ecological Applications, 30(2), e02028. https://doi.org/10.1002/eap.2028

Fuentes-Montemayor, E., Park, K. J., Cordts, K., & Watts, K. (2022). The long-term development of temperate woodland creation sites: from tree saplings to mature woodlands. Forestry: An International Journal of Forest Research, 95(1), 28-37. https://doi.org/10.1093/forestry/cpab027

Hinsley, S. A., & Bellamy, P. E. (2000). The influence of hedge structure, management and landscape context on the value of hedgerows to birds: A review. Journal of Environmental Management, 60(1), 33-49. https://doi.org/10.1006/jema.2000.0360

Jackson, H. B. & Fahrig, L. (2014). Are ecologists conducting research at the optimal scale? Global Ecology and Biogeography 24, 52-63 https://doi.org:10.1111/geb.12233

Kirby, K. J., Pyatt, D. G., & Rodwell, J. (2012). Characterization of the woodland flora and woodland communities in Britain using Ellenberg values and functional analysis. In I. D. Rotherham, M. Jones, & C. Handley (Eds.), Working and walking in the footsteps of ghosts: volume 1 the wooded landscape' (pp. 66–86). Sheffield.

Kudsk, P., Jørgensen, L. N., & Ørum, J. E. (2018). Pesticide Load—A new Danish pesticide risk indicator with multiple applications. Land Use Policy, 70, 384-393. https://doi.org/10.1016/j.landusepol.2017.11.010

Lewis, K. A., Tzilivakis, J., Warner, D. J., & Green, A. (2016). An international database for pesticide risk assessments and management. Human and Ecological Risk Assessment: An International Journal, 22(4), 1050-1064. https://doi.org/10.1080/10807039.2015.1133242

Li, Y., Miao, R., & Khanna, M. (2020). Neonicotinoids and decline in bird biodiversity in the United States. Nature Sustainability, 3(12), 1027-1035. https://doi.org/10.1038/s41893-020-0582-x

Luff, M L. (1998). Provision atlas of the ground beetles (Coleoptera, Carabidae) of Britain. Huntingdon, Biological Records Centre.

Luff, M.L. (2007), The Carabidae (ground Beetles) of Britain and Ireland. RES Handbooks for the Identification of British Insects, Volume 4, Part 2.

Natural England (2023). Ancient woodland (England). In Available under Open Government Licence. Available from https://naturalengland-defra.opendata.arcgis.com/datasets/ancient-woodland-england/explore.

Rigal, S., Dakos, V., Alonso, H., Aunins, A., Benko, Z., Brotons, L., Chodkiewicz, T., Chylarecki, P., de Carli, E., Del Moral, J. C., Domsa, C., Escandell, V., Fontaine, B., Foppen, R., Gregory, R., Harris, S., Herrando, S., Husby, M., Ieronymidou, C., . . . Devictor, V. (2023). Farmland practices are driving bird population decline across Europe. Proc Natl Acad Sci U S A, 120(21), e2216573120. https://doi.org/10.1073/pnas.2216573120

Robinson, R. A., & Sutherland, W. J. (2002). Post-war changes in arable farming and biodiversity in Great Britain. Journal of Applied Ecology, 39(1), 157-176. https://doi.org/10.1046/j.1365-2664.2002.00695.x

Rowland, C. S., Morton, R. D., Carrasco, L., O'Neil, A. W., & Wood, C. M. (2017). Land cover map 2015. NERC Environmental Informaion Data Centre.

Scottish Government (2023). Ancient Woodland Inventory (Scotland). In Available from https://opendata.nature.scot/datasets/ancient-woodland-inventory/explore.

Smith, S., Gilbert, J., Bull, G., Gillam, S., & Whitton, E. (2010). National inventory of woodland and trees (1995–99): methodology.

Vanneste, T., Govaert, S., De Kesel, W., Van Den Berge, S., Vangansbeke, P., Meeussen, C., Brunet, J., Cousins, S. A. O., Decocq, G., Diekmann, M., Graae, B. J., Hedwall, P. O., Heinken, T., Helsen, K., Kapás, R. E., Lenoir, J., Liira, J., Lindmo, S., Litza, K., . . . Biswas, S. (2020). Plant diversity in hedgerows and road verges across Europe. Journal of Applied Ecology, 57(7), 1244-1257. https://doi.org/10.1111/1365-2664.13620

Verheyen, K., Vellend, M., Van Calster, H., Peterken, G., & Hermy, M. (2004). Metapopulation Dynamics in Changing Landscapes: A New Spatially Realistic Model for Forest Plants. Ecology, 85(12), 3302-3312. https://doi.org/10.1890/04-0395

Waddell, E. H., Fuentes-Montemayor, E., Park, K. J., Carey, P., Guy, M., Macgregor, N. A., & Watts, K. (2024). Larger and structurally complex woodland creation sites provide greater benefits for woodland plants. Ecological Solutions and Evidence, 5, e12339. https://doi.org/10.1002/2688-8319.12339

Watts, K., Fuentes-Montemayor, E., Macgregor, N. A., Peredo-Alvarez, V., Ferryman, M., Bellamy, C., Brown, N., & Park, K. J. (2016). Using historical woodland creation to construct a long-term, large-scale natural experiment: the WrEN project. Ecol Evol, 6(9), 3012-3025. https://doi.org/10.1002/ece3.2066

Whytock, R. C., Fuentes-Montemayor, E., Watts, K., Barbosa De Andrade, P., Whytock, R. T., French, P., Macgregor, N. A., & Park, K. J. (2018). Bird-community responses to habitat creation in a long-term, large-scale natural experiment. Conserv Biol, 32(2), 345-354. https://doi.org/10.1111/cobi.12983
