# Supplementary material for: Spillovers and legacies of land management on temperate woodland biodiversity
Source: Nat Ecol Evol. 2025 Apr 23;9(6):1009–20. doi: 10.1038/s41559-025-02688-6 (PMC12148928; doi:10.1038/s41559-025-02688-6)
Supplement: Supplementary file 2 — Reporting Summary [file 41559_2025_2688_MOESM2_ESM.pdf]

nature portfolio

Corresponding author(s): Tom Bradfer-Lawrence

Last updated by author(s): Feb 27, 2025

Reporting Summary

Nature Portfolio wishes to improve the reproducibility of the work that we publish. This form provides structure for consistency and transparency in reporting. For further information on Nature Portfolio policies, see our Editorial Policies and the Editorial Policy Checklist.

Statistics

For all statistical analyses, confirm that the following items are present in the figure legend, table legend, main text, or Methods section.

n/a	Confirmed	
☐	☒	The exact sample size (n) for each experimental group/condition, given as a discrete number and unit of measurement
☐	☒	A statement on whether measurements were taken from distinct samples or whether the same sample was measured repeatedly
☐	☒	The statistical test(s) used AND whether they are one- or two-sided<br>*Only common tests should be described solely by name; describe more complex techniques in the Methods section.*
☐	☒	A description of all covariates tested
☐	☒	A description of any assumptions or corrections, such as tests of normality and adjustment for multiple comparisons
☐	☒	A full description of the statistical parameters including central tendency (e.g. means) or other basic estimates (e.g. regression coefficient) AND variation (e.g. standard deviation) or associated estimates of uncertainty (e.g. confidence intervals)
☒	☐	For null hypothesis testing, the test statistic (e.g. F, t, r) with confidence intervals, effect sizes, degrees of freedom and P value noted<br>*Give P values as exact values whenever suitable.*
☐	☒	For Bayesian analysis, information on the choice of priors and Markov chain Monte Carlo settings
☒	☐	For hierarchical and complex designs, identification of the appropriate level for tests and full reporting of outcomes
☒	☐	Estimates of effect sizes (e.g. Cohen's d, Pearson's r), indicating how they were calculated

Our web collection on statistics for biologists contains articles on many of the points above.

Software and code

Policy information about availability of computer code

Data collection	No software used in data collection.
Data analysis	Modelling was conducted in R software (v.4.2.3) using the package “spOccupancy” (v0.7.6). Variance Inflation Factors were checked with the “usdm” R package (v2.1.7). Code is available from Zenodo: https://doi.org/10.5281/zenodo.14946190

For manuscripts utilizing custom algorithms or software that are central to the research but not yet described in published literature, software must be made available to editors and reviewers. We strongly encourage code deposition in a community repository (e.g. GitHub). See the Nature Portfolio guidelines for submitting code & software for further information.

Data

Policy information about availability of data

All manuscripts must include a data availability statement. This statement should provide the following information, where applicable:

- Accession codes, unique identifiers, or web links for publicly available datasets
- A description of any restrictions on data availability
- For clinical datasets or third party data, please ensure that the statement adheres to our policy

All data used in this study are available from Zenodo: https://doi.org/10.5281/zenodo.14946190

Research involving human participants, their data, or biological material

Policy information about studies with human participants or human data. See also policy information about sex, gender (identity/presentation), and sexual orientation and race, ethnicity and racism.

Reporting on sex and gender	NA
Reporting on race, ethnicity, or other socially relevant groupings	NA
Population characteristics	NA
Recruitment	NA
Ethics oversight	NA

Note that full information on the approval of the study protocol must also be provided in the manuscript.

Field-specific reporting

Please select the one below that is the best fit for your research. If you are not sure, read the appropriate sections before making your selection.

☐ Life sciences ☐ Behavioural & social sciences ☒ Ecological, evolutionary & environmental sciences

For a reference copy of the document with all sections, see nature.com/documents/nr-reporting-summary-flat.pdf

Ecological, evolutionary & environmental sciences study design

All studies must disclose on these points even when the disclosure is negative.

Study description	We explore the combined effects of spatial spillovers and temporal legacies of temperate agricultural land management using Bayesian hierarchical occupancy models.
Research sample	Data from 373 species across 4 contrasting taxa (ground beetles, birds, small terrestrial mammals, vascular plants). Collected from a chronosequence of 134 woodlands in the UK. The sites are part of a long-running experiment (the WrEN project) and all data had already been collected prior to this study.
Sampling strategy	Sampling methods differed across the 4 taxa, but used standardised methods from the literature. While not exhaustive, they were comprehensive.
Data collection	Data collected either by field assistants with species identification by experts in the lab (beetles), or by expert ecologists in the field birds, mammals, plants). Beetles (Coleoptera) were sampled using pitfall traps; birds were surveyed using Common Bird Census Methodology; small mammals were live-trapped and then released unharmed; plants using a comprehensive walk over.
Timing and spatial scale	Beetles: 20 sites in July and August 2013, and 40 sites between June and August 2014. Birds: 79 sites were visited once per month in April, May and June in 2015, and 46 sites were surveyed twice over the same period in 2017. Small terrestrial mammals: 31 sites in 2013 and 69 sites in 2014; Vascular plants: 132 sites in 2015 and 2016.
Data exclusions	Singleton species (i.e., those only recorded once) were removed from all taxa. We removed bird species that flew over sites or whose presence was due to water features rather than woodland (e.g., Grey Heron, Dipper). We excluded tree species, as the woodland sites are planted, so presence of a particular tree species does not necessarily reflect site conditions or context.
Reproducibility	Observational data rather than experimental
Randomization	Not relevant, no experiments conducted.
Blinding	Not relevant, no experiments conducted.

Did the study involve field work? ☒ Yes ☐ No

Field work, collection and transport

Field conditions	Sites span two regions of the UK, one in England, one in Scotland. These have a temperate climate, some rainfall occurred during sampling, but not cold weather. Bird and mammal surveys were not conducted during inclement weather.

Location	Woodland sites are in two lowland landscapes in the UK. Full descriptions are in Watts et al (2016) Ecology & Evolution and Fuentes-Montemayor et al (2022) Forestry.
Access & import/export	NA
Disturbance	Disturbance was minimal.

Reporting for specific materials, systems and methods

We require information from authors about some types of materials, experimental systems and methods used in many studies. Here, indicate whether each material, system or method listed is relevant to your study. If you are not sure if a list item applies to your research, read the appropriate section before selecting a response.

Materials & experimental systems

n/a	Involved in the study	
☒	☐	Antibodies
☒	☐	Eukaryotic cell lines
☒	☐	Palaeontology and archaeology
☐	☒	Animals and other organisms
☒	☐	Clinical data
☒	☐	Dual use research of concern
☒	☐	Plants

Methods

n/a	Involved in the study	
☒	☐	ChIP-seq
☒	☐	Flow cytometry
☒	☐	MRI-based neuroimaging

Animals and other research organisms

Policy information about studies involving animals; ARRIVE guidelines recommended for reporting animal research, and Sex and Gender in Research

Laboratory animals	Did not involve laboratory animals.
Wild animals	Only applicable to small terrestrial mammals. Ugglan traps #2 (multi-catch wire mesh traps with roof covers; Grahnab, Sweden) were deployed for four continuous nights at each site and checked every morning. Traps were baited with food and bedding material was provided. Four species were caught: Apodemus sylvaticus, Microtus agrestis, Myodes glareolus and Sorex araneus. Captured individuals were identified to species, temporarily marked by fur clipping to identify recaptures, and immediately released at the site of capture. Further description of protocol details can be found in Fuentes-Montemayor et al. (2021) Ecological Applications https://doi.org/10.1002/eap.2028
Reporting on sex	Not applicable.
Field-collected samples	No samples taken from the field.
Ethics oversight	Study approved by the University of Stirling ethics committee.

Note that full information on the approval of the study protocol must also be provided in the manuscript.

Plants

Seed stocks	*Report on the source of all seed stocks or other plant material used. If applicable, state the seed stock centre and catalogue number. If plant specimens were collected from the field, describe the collection location, date and sampling procedures.*
Novel plant genotypes	*Describe the methods by which all novel plant genotypes were produced. This includes those generated by transgenic approaches, gene editing, chemical/radiation-based mutagenesis and hybridization. For transgenic lines, describe the transformation method, the number of independent lines analyzed and the generation upon which experiments were performed. For gene-edited lines, describe the editor used, the endogenous sequence targeted for editing, the targeting guide RNA sequence (if applicable) and how the editor was applied.*
Authentication	*Describe any authentication procedures for each seed stock used or novel genotype generated. Describe any experiments used to assess the effect of a mutation and, where applicable, how potential secondary effects (e.g. second site T-DNA insertions, mosiacism, off-target gene editing) were examined.*
